# The DAG/PKC/CREB1/TGF-β1 axis drives shear-wave elastography stiffness and malignant progression in triple-negative breast cancer via lipid metabolic reprogramming

**DOI:** 10.1038/s41419-026-08625-0

**Published:** 2026-03-20

**Authors:** Shiyu Wang, Dongdong Zheng, Ziqi Wang, Ruoqing Hou, Zhiming Zhang, Zhanping You, Jin Zhou, Yunxia Huang, Mengyao Quan, Jian Zhou, Cai Chang, Shichong Zhou

**Affiliations:** Department of Ultrasound, Fudan University Shanghai Cancer Center; Department of Oncology, Shanghai Medical College, Fudan University, Shanghai, China

**Keywords:** Translational research, Preclinical research

## Abstract

In clinical practice, triple-negative breast cancer (TNBC) patients with varying levels of lipid metabolism exhibit differences in tumor shear-wave elastography (SWE) stiffness and prognosis, but this association with unclear mechanism. In this study, a clinical cohort from FUSCC (n = 147) demonstrated that both elevated BMI and higher SWE stiffness were significantly associated with poorer long-term prognosis in TNBC patients, and these associations were further validated in multi-TNBC animal models. Our findings emphasize the role of SWE stiffness in capturing BMI-related alterations in the tumor mechanical microenvironment. Based on integrated lipidomic and transcriptomic analyses, we demonstrated that diacylglycerol (DAG) serves as a critical lipid molecule promoting elevated SWE stiffness and malignant progression. Mechanistically, DAG upregulates TGF-β1 expression through PKC-mediated enhancement of CREB1 phosphorylation in multiple TNBC cell lines, directly promoting TNBC progression and activating cancer-associated fibroblasts. This creates a self-sustaining feedback loop that accelerates malignancy. Finally, we confirmed that the DAG/PKC/CREB1/TGF-β1 signaling axis profoundly regulates SWE imaging stiffness in TNBC models, with further validation in clinical samples. Our study establishes SWE stiffness as a non-invasive imaging biomarker for the activation of this specific pro-metastatic pathway, providing a mechanistic basis for interpreting SWE features through a biological lens and paving the way for its application in prognosis prediction and tailored therapeutic strategies for high-risk TNBC patients.

## Introduction

Triple-negative breast cancer (TNBC) is the most aggressive breast cancer subtype [1], with limited treatments and higher risks of early recurrence and distant metastasis, leading to poor prognosis [2, 3]. The growing global burden of breast cancer is exacerbated by obesity, with deaths and disability attributable to high body-mass index (BMI) increasing by 46% and 53%, respectively, between 2010 and 2019 [4]. This increase in mortality risk is largely attributable to weight gain-induced heightened intratumoral lipid metabolism, promoting tumor progression, thereby worsening the prognosis of obese TNBC patients. This underscores the urgent need for non-invasive methods to dynamically assess metabolic alterations within tumors, which could significantly improve prognostic accuracy and personalized treatment strategies.

SWE, as an advanced ultrasound imaging tool, offers unique advantages. It sensitively and in real time detects tissue stiffness changes caused by dysregulated lipid metabolism. This aids in breast cancer diagnosis and metabolic subtyping [5, 6]. Although the strong association between obesity and TNBC aggressiveness is well-established [7], a critical yet underrecognized clinical opportunity lies in the use of ultrasound-based assessments like SWE to quantitatively capture and predict this relationship. This imaging modality is therefore of particular interest in TNBC, where obesity is a potent risk factor for tumor aggression.

Obesity promotes tumor progression through metabolic reprogramming. A key aspect is aberrant lipid metabolism. It supports cancer aggressiveness by supplying energy and biomolecules for growth and by producing signaling lipids like diacylglycerol (DAG) [8]. DAG acts as a critical second messenger that activates multiple oncogenic pathways, notably through protein kinase C (PKC), which regulates genes involved in proliferation, survival, and invasion [8–10]. Furthermore, lipid reprogramming extends beyond cancer cells to the tumor microenvironment (TME), where it activates cancer-associated fibroblasts (CAFs). These activated CAFs secrete excess collagen, stiffening the stroma and facilitating invasion, metastasis, and angiogenesis [11–15]. This establishes a feedforward loop in which metabolic changes drive mechanical changes in the TME, which in turn promote malignant phenotypes.

SWE offers a unique window into this process by imaging tissue stiffness. We have previously demonstrated that SWE can quantify CAFs distribution and predict treatment response in TNBC [16–18], with stiffer tissues typifying malignant lesions and TNBC specifically [19, 20]. Importantly, SWE may detect subtle lipid-mediated stromal alterations even before overt weight gain or metabolic symptoms occur—a critical advantage since obese TNBC patients face approximately 30% higher recurrence and mortality [21]. However, the molecular mechanisms linking obesity-driven lipid metabolism to stromal stiffening and SWE-detectable changes remain poorly defined, limiting its clinical translation for prognostic imaging.

In this study, we aimed to bridge this mechanistic gap by integrating clinical imaging, multi-omics, and functional experiments. We identified a positive correlation between BMI, SWE stiffness, and poor prognosis in a TNBC cohort. We further demonstrated that obesity-induced DAG accumulation sustains activation of the PKC/CREB1/TGF-β1 axis, which enhances tumor cell aggressiveness and initiates a TGF-β1-mediated feedback loop between tumor cells and CAFs. This process dynamically increases stromal stiffness measurable by SWE and accelerates metastatic progression. Our findings elucidate a novel signaling pathway connecting lipid reprogramming to biomechanical remodeling in TNBC and establish SWE stiffness as a functional imaging biomarker, paving the way for image-guided precision therapy.

## Results

### High BMI promotes TNBC proliferation and modulates ultrasonic imaging features via lipid metabolic reprogramming

**Fig. 1:**
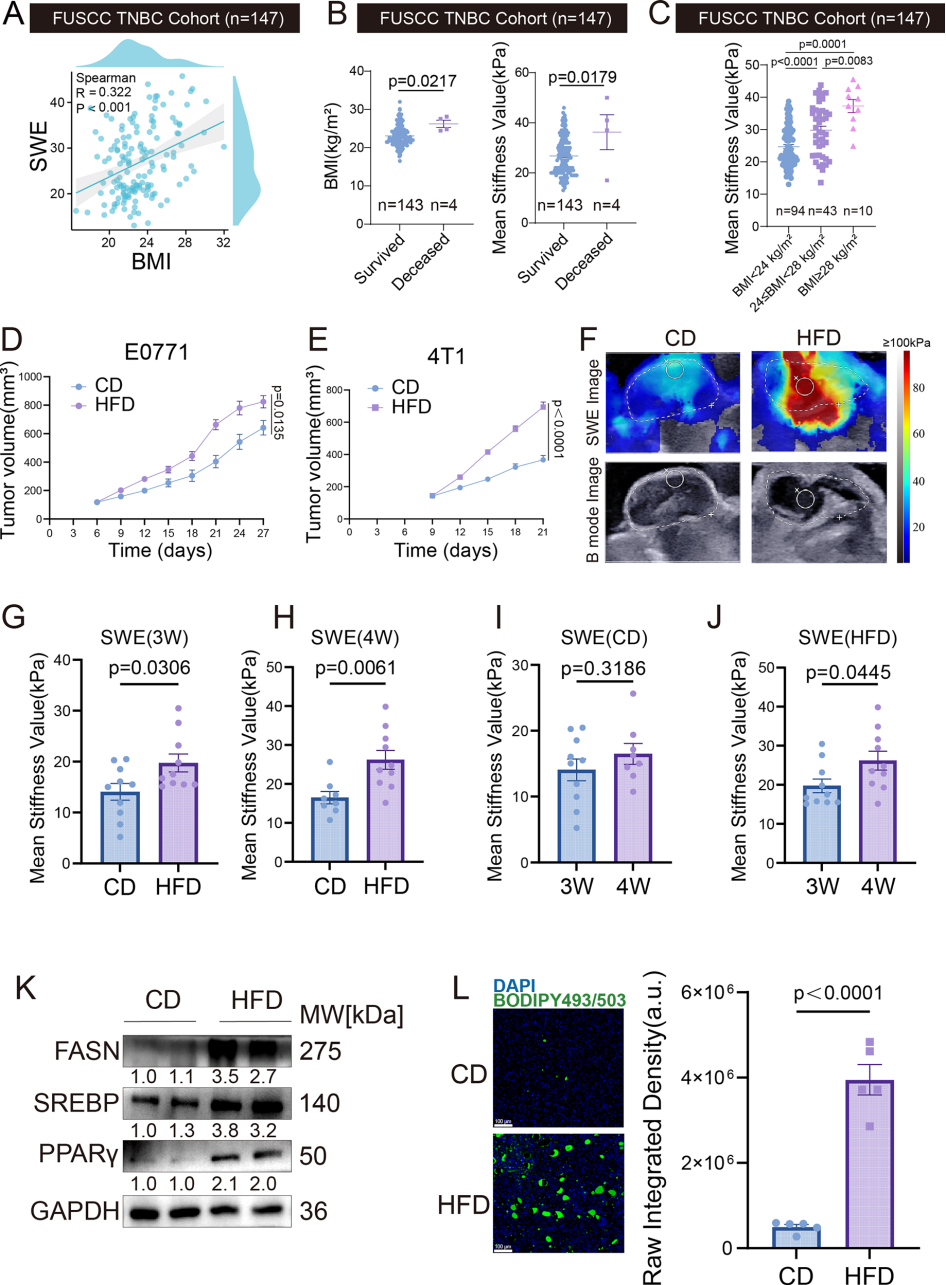
High BMI promotes TNBC proliferation and modulates ultrasonic imaging features via lipid metabolic reprogramming. Analyses based on the FUSCC TNBC cohort(*n* = 147): **A** Correlation between BMI and SWE values. **B** Comparison of BMI (left) and SWE values (right) between deceased and surviving patients. **C** Comparison of SWE values between patients with BMI < 24 kg/m², 28 > BMI ≥ 24 kg/m² and BMI ≥ 28 kg/m². Female C57BL/6J mice were fed a high-fat diet (HFD) or a control diet (CD) for 8 weeks to establish obesity and control models, respectively. Then E0771 cells (**D**) and 4T1 cells (**E**) were orthotopically injected into the mammary fat pad. Tumor growth curves in CD and HFD mice (*n* = 10). The tumor volume was measured every 3 days. Analyses based on the E0771 mouse model: **F** SWE and B-mode ultrasound images of tumors from CD and HFD mice. Comparison of SWE-based tumor stiffness between CD and HFD groups at 3 weeks (**G**) and 4 weeks (**H**) post-inoculation. Comparison of SWE values in CD mice (**I**) and HFD mice (**J**) between 3 and 4 weeks. **K** Western blot analysis of lipid metabolism-related proteins (FASN, SREBP, PPARγ) in E0771 tumors from CD and HFD mice. **L** BODIPY staining and quantitative analysis of neutral lipid droplets in tumors from CD and HFD mice. Scale bar, 100 μm. Data are presented as mean ± SEM.

Based on the clinical cohort system of Fudan University Shanghai Cancer Center (FUSCC), this study evaluated the clinical value of BMI. Multivariate COX proportional hazard regression models indicated a statistically significant correlation between BMI levels and long-term prognosis in TNBC patients (Table 1). Nevertheless, the mechanisms linking obesity to TNBC progression are poorly understood. Given that obesity is known to remodel the TME and alter tissue mechanical properties, we hypothesized that BMI might be associated with measurable changes in tumor stiffness. To test this hypothesis, we employed SWE to quantitatively assess tissue stiffness non-invasively. Spearman correlation tests revealed certain correlations between BMI and SWE stiffness, as well as grayscale values under B-mode ultrasound (Figs. 1A and S1A). Further comparisons showed that BMI and SWE stiffness values were significantly higher in the deceased patient group than in the survival group (Fig. 1B). Although grayscale values were also lower in the deceased group, the difference was not statistically significant (Fig. S1B). Considering that our FUSCC TNBC cohort consists exclusively of Chinese patients, we classified BMI according to Chinese criteria as normal weight (BMI < 24 kg/m²), overweight (24 ≤ BMI < 28 kg/m²), and obese (BMI ≥ 28 kg/m²) [22, 23]. We first analyzed the cohort using a two-group comparison (BMI ≥ 24 kg/m² vs. BMI < 24 kg/m²). Notably, the SWE stiffness value was significantly higher in the higher BMI group (Fig. S1C), while the grayscale value was significantly lower (Fig. S1D). To obtain a more precise assessment, we further stratified patients into the three BMI categories defined above. This refined analysis revealed that SWE stiffness values showed a progressive increase across the three groups with statistically significant differences (Fig. 1C). In contrast, although grayscale values tended to be lower in higher BMI groups, not all pairwise comparisons reached statistical significance (Fig. S1E). To further validate the association between BMI and tumor progression at the histological level, we performed double immunofluorescence (IF) staining for COL1A1 and Ki67 on tumor samples from the three patient groups (normal, overweight, and obese). Our results demonstrated a stepwise increase in both COL1A1 deposition and Ki67-positive proliferating cells with ascending BMI levels (Fig. S1F, G), providing direct pathological evidence linking obesity to enhanced tumor fibrosis and proliferation. These results show that BMI is an independent prognostic factor. It also has significant clinical links to ultrasonic features, like SWE stiffness and grayscale values.

**Table 1:** Multivariate Cox regression analysis of prognostic factors in the FUSCC TNBC cohort.

Characteristics	Total(N)	Univariate analysis		Multivariate analysis	
		Hazard ratio (95% CI)	*P* value	Hazard ratio (95% CI)	*P* value
Age (year)	147	1.041 (0.946–1.145)	0.408		
Palpable ALN	147				
No	119	Reference		Reference	
Yes	28	4.749 (0.668–33.771)	0.120	7.404 (0.927–59.154)	0.059
Breast cancer family history	147				
No	140	Reference		Reference	
Yes	7	6.661 (0.691–64.254)	0.101	5.920 (0.534–65.690)	0.148
Menopausal status	147				
Premenopausal	82	Reference			
Postmenopausal	65	1.405 (0.197–9.997)	0.734		
BMI (kg/m²)	147	1.344 (1.011–1.785)	0.041	1.434 (1.031–1.995)	0.032
Gray-scale(a.u.)	147	0.993 (0.971–1.015)	0.517		
SWE(kPa)	147	1.100 (0.965–1.253)	0.155	1.023 (0.891–1.176)	0.744
Ki-67 expression (%)	147	1.014 (0.979–1.051)	0.437		
Ki-67 expression (binary classification)	147				
High	105	Reference		Reference	
Low	42	0.000 (0.000–Inf)	0.999	0.000 (0.000–Inf)	0.998

*BMI* body mass index, *ALN* axillary lymph node, *SWE* shear wave elastography.

To simulate the variation in BMI, we employed a dietary intervention model wherein C57BL/6 mice were fed either a high-fat diet (HFD) or a control diet (CD) (Fig. S2A). The body weight of the HFD group was significantly higher than that of the CD group (Fig. S2B). After inoculation with E0771 tumor cells, the tumor volume in the HFD group was significantly larger than that in the CD group (Figs. 1D and S2C). We performed ultrasound examinations at the 3rd and 4th weeks after tumor inoculation (Figs. 1F & S2D). Tumors in the HFD group showed significantly lower grayscale values than the CD group at both time points (Fig. S2E, F). However, neither group exhibited significant changes in grayscale values over time (Fig. S2G, H).

In contrast, the SWE stiffness in the HFD group was significantly higher than that in the CD group at both examination points (Fig. 1G, H). SWE stiffness increased significantly over time in the HFD group but showed no significant change in the CD group (Fig. 1I, J). To investigate the underlying ECM alterations responsible for the increased stiffness, we performed Sirius Red staining, IF for COL1A1 and α-SMA. Given its critical role as the enzyme that catalyzes collagen cross-linking to stabilize and stiffen the ECM, we also quantified Lysyl Oxidase (LOX) expression levels in tumors from both groups [24]. Consistent with the mechanical measurements, the HFD group exhibited significantly higher levels of collagen deposition, COL1A1 and α-SMA expression, and LOX activity compared to the CD group (Fig. S2I–M). Therefore, we focused subsequent research on SWE stiffness. This phenomenon was also validated in the 4T1 tumor model (Figs. 1E and S3A–I). The protein expression levels of three key lipid metabolism markers—FASN, SREBP1, and PPARγ—were significantly elevated in the tumor tissues of the HFD group (Figs. 1K and S3J). Concurrently, Bodipy fluorescence confirmed a significant increase in lipid droplet density in the HFD group tumor tissues (Fig. 1L), indicating enhanced lipid metabolism in the HFD group. Overall, our findings indicate that high BMI promotes tumor progression by activating lipid metabolic reprogramming. Additionally, it dynamically alters ultrasonic imaging characteristics, most notably by increasing SWE stiffness.

### Integrated lipidomics-transcriptomics analysis reveals DAG/PKC mediates fibrosis in TNBC induced by lipid metabolic reprogramming

**Fig. 2:**
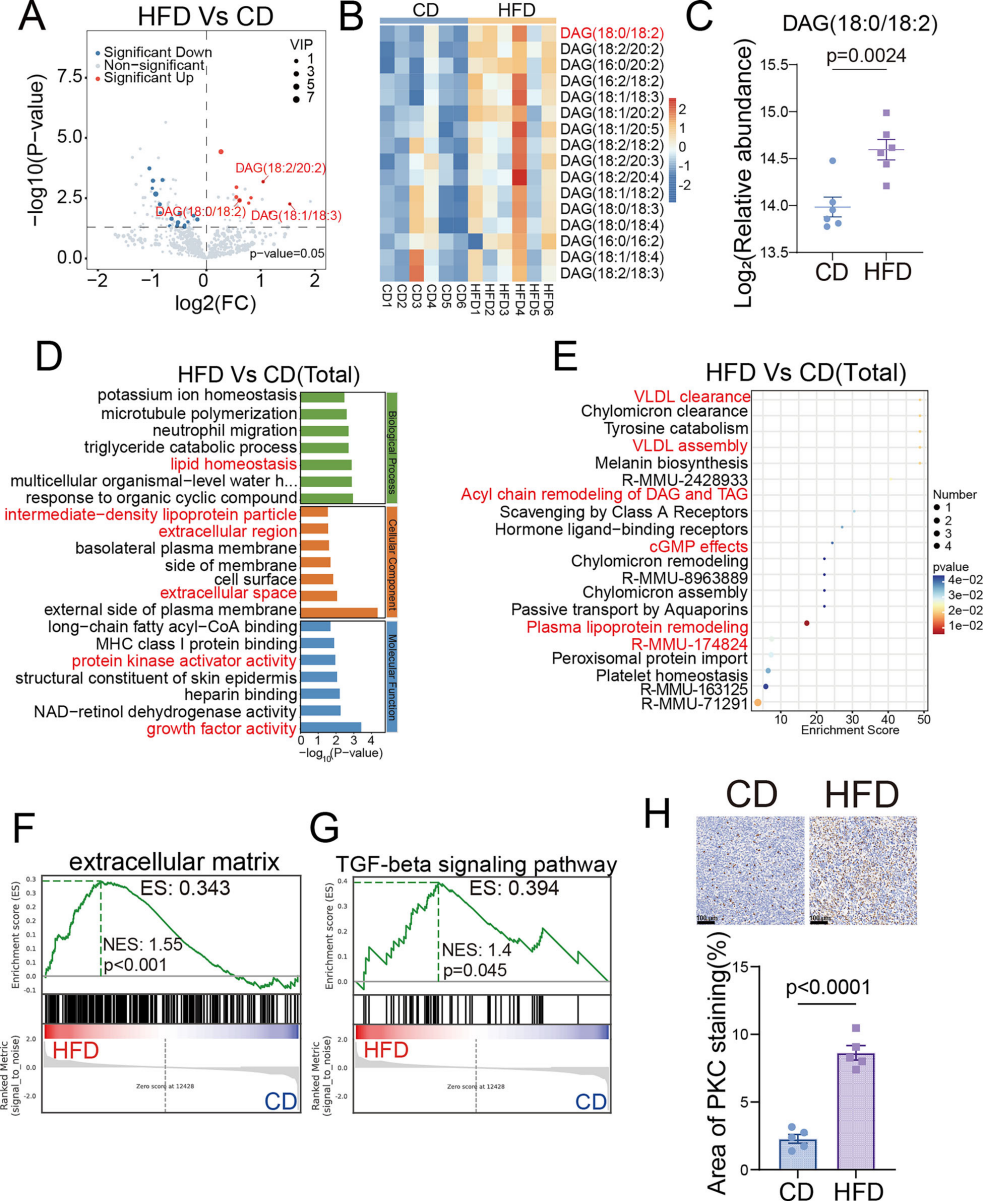
Integrated lipidomics-transcriptomics analysis reveals DAG/PKC Mediates fibrosis in TNBC induced by lipid metabolic reprogramming. **A** Volcano plots of differentially expressed metabolites between the CD and HFD groups (*n* = 6 per group). **B** Heatmap depicting the relative abundance of differentially altered DAG species by LC-MS/MS-based lipidomics in CD and HFD groups (*n* = 6 per group). Rows represent individual DAG species, and columns represent individual biological samples. **C** The log₂-transformed relative abundance of DAG (18:0/18:2) was determined by LC-MS/MS-based lipidomics across CD and HFD groups. **D** GO analysis of DEGs enrichment in RNA-seq data from the CD and HFD groups; the pathways of interest are marked in red. **E** Reactome enrichment analysis of DEGs enrichment in RNA-seq data from the CD and HFD groups; the pathways of interest are marked in red. **F**, **G** Individual GSEA plots of the extracellular matrix and TGF-beta signaling pathways in RNA-seq data from the CD and HFD groups. **H** Representative IHC images and quantitative analysis of PKC expression in tumors from CD and HFD mice. Scale bar, 100 μm. Data are presented as mean ± SEM.

To identify the potential molecular mechanisms through which lipid metabolism regulates SWE stiffness in TNBC, this study performed integrated lipidomics and transcriptomics analyses on tumor tissues from both mouse groups. Lipidomics results showed clear separation between groups via principal component analysis (PCA) of 756 identified lipids (Fig. S4A). Compared to the CD group, multiple DAG levels were significantly elevated in the HFD group, with DAG (18:0/18:2) being the differential lipid with the highest variable importance in projection (VIP) score (Fig. 2A–C). Gene Ontology (GO) enrichment analysis of transcriptomics data revealed significant activation of lipid homeostasis, extracellular region, protein kinase activity, and growth factor activity (Fig. 2D). Reactome enrichment analysis showed significant enrichment in lipoprotein assembly, cyclic guanosine monophosphate (cGMP) effects, and DAG/triglyceride (TAG) acyl chain remodeling (Fig. 2E). Gene Set Enrichment Analysis (GSEA) indicated that obesity-driven hyperactivation of lipid metabolism significantly activated invasion-related pathways such as extracellular matrix, cell adhesion molecules, and synthesis of IP3 and IP4 in the cytoplasm (Figs. 2F and S4B, C). These showed aberrant lipid metabolic reprogramming as a key characteristic of the obese TME. Notably, the significant enrichment of DAG remodeling and protein kinase activity pathways, together with the established knowledge that protein kinase C (PKC) is a direct downstream target of DAG [25, 26], collectively indicate the potential activation of the DAG/PKC signaling axis. Furthermore, the robust enrichment of extracellular matrix and cell adhesion pathways provides a direct molecular link to stromal fibrosis. It also significantly upregulated fibrosis-related pathways, including TGF-β signaling, elastic fiber formation, and pulmonary fibrosis (Figs. 2G and S4D, E). We confirmed via immunohistochemical (IHC) staining that PKC expression was significantly higher in the HFD group than in the CD group (Fig. 2H). Our multi-omics analysis reveals obesity-linked DAG accumulation, specifically DAG (18:0/18:2). This occurs alongside activated fibrosis pathways and increased PKC expression. Together, these findings suggest the DAG/PKC axis may regulate tissue fibrosis in TNBC tumors. These findings provide promising avenues for future research into the mechanistic links between obesity and TME remodeling, although the exact molecular pathways and functional implications require further elucidation.

### PKC-dependent DAG signaling drives TNBC metastasis by activating EMT

**Fig. 3:**
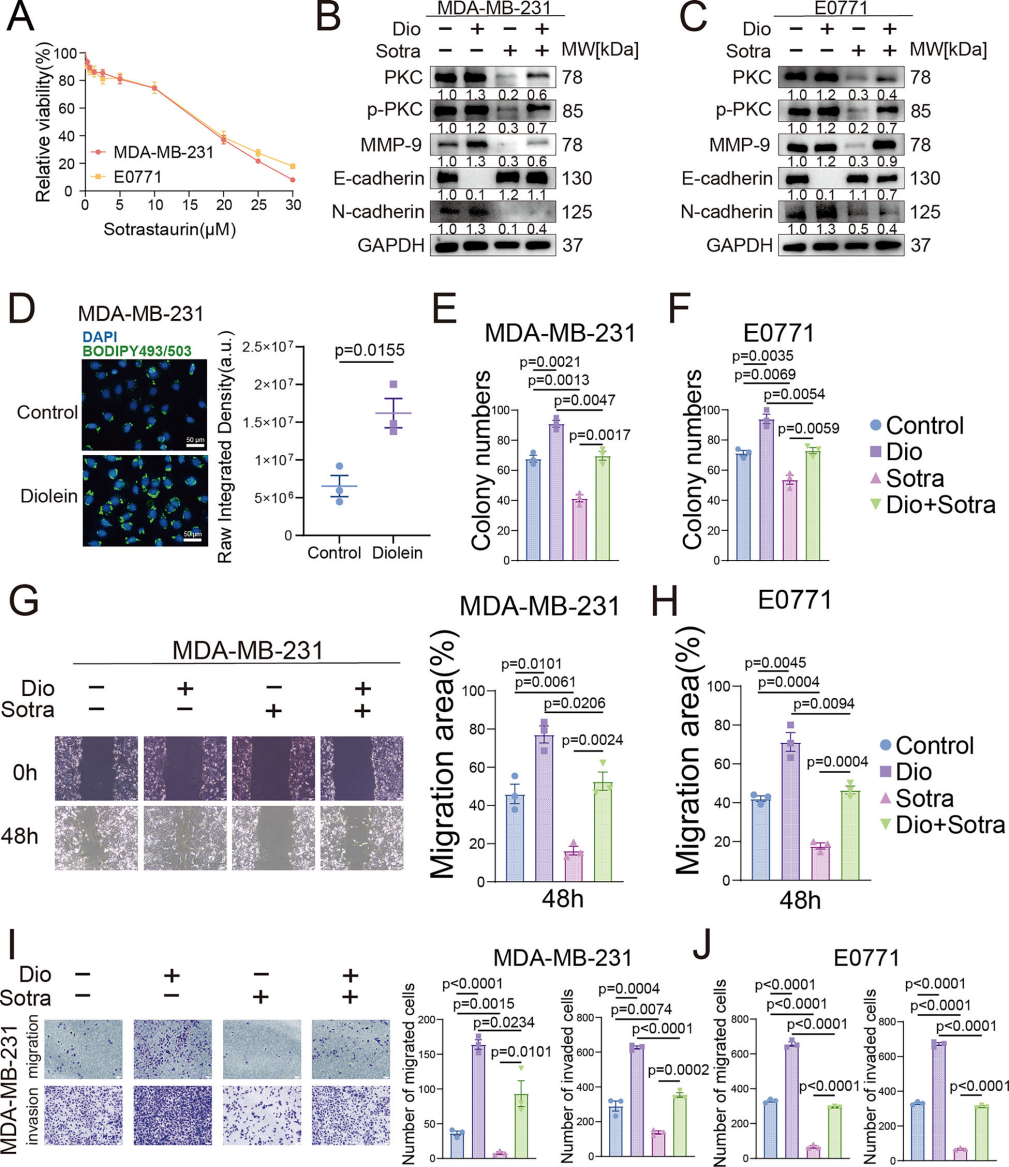
PKC-dependent DAG signaling drives TNBC metastasis by activating EMT. **A** Cell viability measured by CCK-8 assay in MDA-MB-231 and E0771 cells treated with increasing concentrations of Sotrastaurin. Western blot analysis of PKC, phosphorylated PKC (p-PKC), and epithelial–mesenchymal transition (EMT)-related markers in MDA-MB-231 (**B**) and E0771 (**C**) cells after treatment with Diolein and Sotrastaurin. **D** BODIPY staining and quantitative analysis of lipid droplets in MDA-MB-231 cells treated with Diolein. Scale bars, 50 μm. Quantitative results of colony formation assays in MDA-MB-231 (**E**) and E0771 (**F**) cells treated with Diolein and Sotrastaurin. Wound healing assay and quantitative analysis of migration in MDA-MB-231 (**G**) and E0771 (**H**) cells treated with Diolein and Sotrastaurin. Scale bars, 100 μm. Transwell invasion and migration assays with quantitative results in MDA-MB-231 (**I**) and E0771 (**J**) cells after treatment with Diolein and Sotrastaurin. Scale bars, 100 μm. Data are presented as mean ± SEM.

To better understand the intrinsic mechanisms by which DAG influences the ultrasonic characteristics of TNBC, we investigate the biological roles of DAG and PKC in TNBC. As a direct downstream target of DAG, PKC’s role was evaluated. This study first treated TNBC cell lines (MDA-MB-231 and E0771) with the PKC inhibitor Sotrastaurin. Results showed that Sotrastaurin significantly inhibited cell viability in a dose-dependent manner, leading to the selection of 15 μM for subsequent experiments (Fig. 3A). Western blot analysis showed that Diolein (DAG (18:0/18:2)) treatment activated PKC. It also up-regulated invasive molecule MMP-9 and the mesenchymal marker N-cadherin. In contrast, the epithelial marker E-cadherin was suppressed. These expression changes were completely reversed by Sotrastaurin intervention (Fig. 3B, C), suggesting the DAG/PKC axis enhances tumor cell invasiveness by inducing epithelial-mesenchymal transition (EMT). Further studies showed that BODIPY staining indicated DAG stimulation significantly increased cellular neutral lipid levels (Figs. 3D and S5A). Colony formation assays demonstrated that activation of the DAG/PKC significantly increased colony formation (Figs. 3E, F & S5B). Meanwhile, wound healing and transwell assays further confirmed that activation of the DAG/PKC significantly enhanced the invasion and migration capabilities of both cell lines (Figs. 3G–J and S5C, D). In summary, our results indicate that DAG promotes TNBC cell lipid metabolic reprogramming, EMT progression, and invasion/migration capabilities through a PKC-dependent mechanism, thereby driving malignant phenotypic development.

### CREB1 functions as a downstream effector of PKC in promoting TNBC malignant progression

To identify transcription factors functionally linked to the PKC-driven fibrotic processes underlying increased SWE stiffness, we employed a targeted strategy. We first performed analysis using online public databases such as JASPAR, which revealed that the promoter region of TGF-β1—a central mediator of fibrosis—contains highly conserved binding motifs for CREB1, providing a compelling predictive link (Fig. S6A). This rationale was further supported by previous studies demonstrating significant crosstalk between the PKC and CREB1 pathways, in which PKC phosphorylates CREB1 either directly or through upstream kinases to enhance its transcriptional activity [27–29]. Furthermore, this factor has been shown to promote proliferation, invasion, and migration in breast cancer [30, 31] and is closely linked to poor clinical prognosis [32]. We therefore specifically sought to investigate this recognized yet mechanistically underexplored axis within the unique pathological context of obesity-associated TNBC fibrogenesis.

**Fig. 4:**
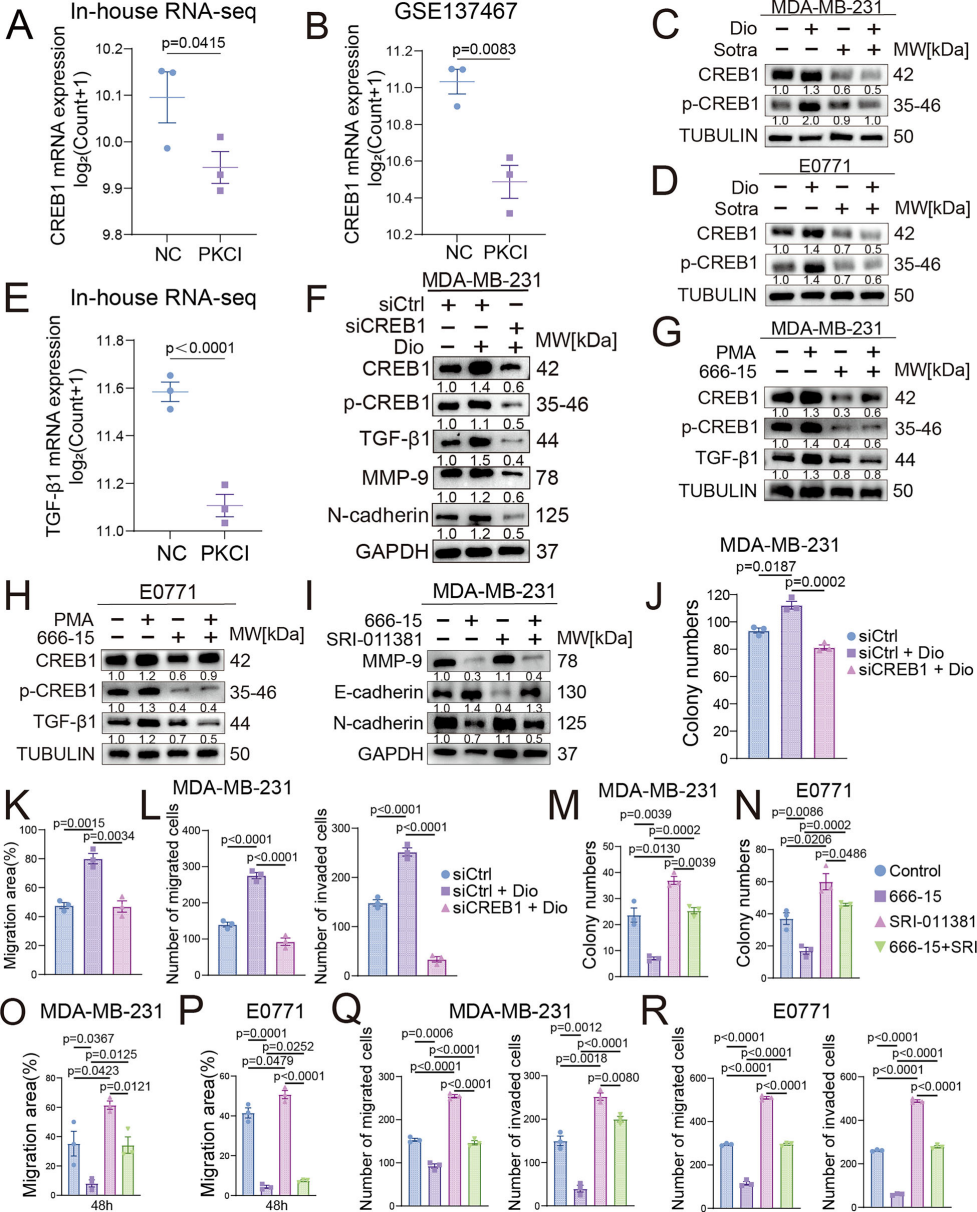
Sotrastaurin suppresses CREB1 expression and activity, disrupting the CREB1/TGF-β1 axis and impairing TNBC cell proliferation, migration, and invasion. **A** CREB1 mRNA expression was downregulated in Sotrastaurin-treated MDA-MB-231 cells compared to controls, as determined by RNA-seq. **B** Analysis of the GEO dataset GSE137467 confirmed that PKC inhibitor treatment also reduced CREB1 mRNA levels in this cell line. Western blot analysis of CREB1 and phosphorylated CREB1 (p-CREB1) in MDA-MB-231 (**C**) and E0771 (**D**) cells treated with Diolein and Sotrastaurin. **E** TGF-β1 mRNA expression was downregulated in Sotrastaurin-treated MDA-MB-231 cells compared to controls, as determined by RNA-seq. **F** Western blot analysis of CREB1, p-CREB1, TGF-β1, MMP9 and N-cadherin in MDA-MB-231 treated with siCREB1 and Diolein. **G** Western blot analysis of CREB1, p-CREB1, and TGF-β1 in MDA-MB-231 (**G**) and E0771 (**H**) cells treated with PMA (PKC activator) and 666-15 (CREB1 inhibitor). **I** Western blot analysis of EMT-related markers in MDA-MB-231 cells treated with 666-15 and SRI-011381 (TGF-β1 activator). Quantitative results of colony formation assays (**J**), wound healing assay (**K**) and transwell invasion and migration assays (**L**) in MDA-MB-231 cells treated with siCREB1 and Diolein. Scale bars 100 μm. Quantitative results of colony formation assays in MDA-MB-231 (**M**) and E0771 (**N**) cells treated with 666-15 and SRI-011381. Wound healing assay and quantitative analysis of migration in MDA-MB-231 (**O**) and E0771 (**P**) cells treated with 666-15 and SRI-011381. Scale bars, 100 μm. Transwell invasion and migration assays with quantitative results in MDA-MB-231 (**Q**) and E0771 (**R**) cells after treatment with 666-15 and SRI-011381. Scale bars, 100μm. Data are presented as mean ± SEM.

To experimentally validate this selection, we performed transcriptome sequencing on MDA-MB-231 cells treated with the PKC inhibitor Sotrastaurin, which confirmed the significant downregulation of CREB1 (Fig. 4A). This phenomenon was corroborated by analysis of the independent GEO dataset GSE137467 (Fig. 4B). Meanwhile, GSEA of our RNA-seq data indicated that PKC inhibition significantly suppressed CREB1 target genes (Fig. S6B). Most definitely, western blot analysis demonstrated that Diolein markedly upregulated CREB1 expression, whereas PKC inhibitor treatment conversely downregulated its levels (Fig. 4C, D). In a word, these findings demonstrate that CREB1 as a key downstream mediator of PKC signaling in promoting TNBC progression.

### CREB1 transcriptionally activates TGF-β1 to drive TNBC malignant progression

Having established the PKC-CREB1 link, we next investigated its functional consequences. Motivated by the initial JASPAR prediction linking CREB1 to the TGF-β1 promoter (Fig. S6A), we re-analyzed our transcriptomic results and found that Sotrastaurin treatment also significantly downregulated TGF-β1 expression (Fig. 4E). Concurrently, Reactome enrichment showed significant involvement in extracellular matrix-related pathways and collagen synthesis/degradation (Fig. S6C), and GSEA indicated that PKC inhibition downregulated the regulation of fibroblast migration (Fig. S6D).

To genetically validate that CREB1 directly regulates TGF-β1, we designed three distinct siRNAs targeting CREB1. Based on knockdown efficiency assessed by western blot, we selected the most effective sequence (siCREB1-1) for subsequent experiments (Fig. S6E). Silencing CREB1 significantly reduced the protein levels of TGF-β1, concomitant with downregulation of the invasive molecule MMP-9 and the mesenchymal marker N-cadherin (Fig. 4F), providing direct genetic evidence that CREB1 regulates TGF-β1 expression and downstream malignant pathways.

To further determine the regulatory relationship using pharmacological tools, we treated two TNBC cell lines with PMA (a PKC activator) and 666-15 (a CREB1 inhibitor). Activating PKC increased the protein expression levels of CREB1, phosphorylated CREB1, and TGF-β1, while inhibiting CREB1 decreased TGF-β1 protein expression (Fig. 4G, H). This functional data, consistent with the bioinformatic prediction, strongly suggests that CREB1 stimulates TGF-β1 transcription.

Functionally, we found that inhibiting CREB1 significantly downregulated MMP-9 and N-cadherin, while upregulating the epithelial marker E-cadherin. Activating TGF-β1 completely reversed these molecular expression changes (Figs. 4I and S6F), genetically placing TGF-β1 functionally downstream of CREB1. Colony formation, wound healing, and transwell assays demonstrated that activation of the CREB1/TGF-β1 axis significantly increased proliferation, migration, and invasion capabilities (Figs. 4J–R and S6G–N). Collectively, these findings delineate a coherent signaling cascade wherein PKC activates CREB1, which in turn transcriptionally upregulates TGF-β1, thereby promoting malignant progression in TNBC.

### TGF-β1-driven tumor cell-CAFs crosstalk promotes TNBC progression

Given previous research indicating that CAFs influence tumor cell SWE stiffness [16–18], together with our above findings that DAG activates the TGF-β1 pathway, we hypothesized that the DAG/PKC/CREB1/TGF-β1 signaling axis might also regulate the interaction between tumor cells and CAFs. To investigate the mechanism of DAG-mediated tumor cell-CAFs interaction, this study used conditioned medium (CM) from DAG-treated E0771 cells (designated as CM(D-7)) to stimulate an NIH-3T3 cell model. Subsequently, the CM from these activated fibroblasts (designated as CM(D-7-3)) was collected to treat tumor cells, establishing a bidirectional communication model. To directly exclude the possibility that the effects of CM(D-7-3) were primarily driven by residual DAG carried over from its induction process, we performed detailed lipidomic profiling, which revealed that levels of multiple DAG species in CM(D-7-3) were quantitatively comparable to the vehicle control and significantly lower than those in CM(D-7) (Fig. S7A, B). Based on relative abundance ratios calibrated against the 3 μg/ml DAG spike in CM(D-7), we estimated the Diolein concentration in CM(D-7-3) to be approximately 0.74 μg/ml. We then designed an exogenous DAG supplementation experiment using 0, 0.5, 1, and 3 μg/ml Diolein concentrations to functionally benchmark its effect (Fig. S7C–E). While 3 μg/ml DAG promoted proliferation and invasion as expected, the lower concentrations (0.5 and 1 μg/ml) failed to do so, mimicking the vehicle control. Strikingly, CM(D-7-3) containing only approximately 0.74 μg/ml DAG robustly induced pro-tumorigenic phenotypes. These results demonstrate that the biological activities of CM(D-7-3) are not mediated by DAG but are attributable to other soluble factors secreted by activated fibroblasts.

**Fig. 5:**
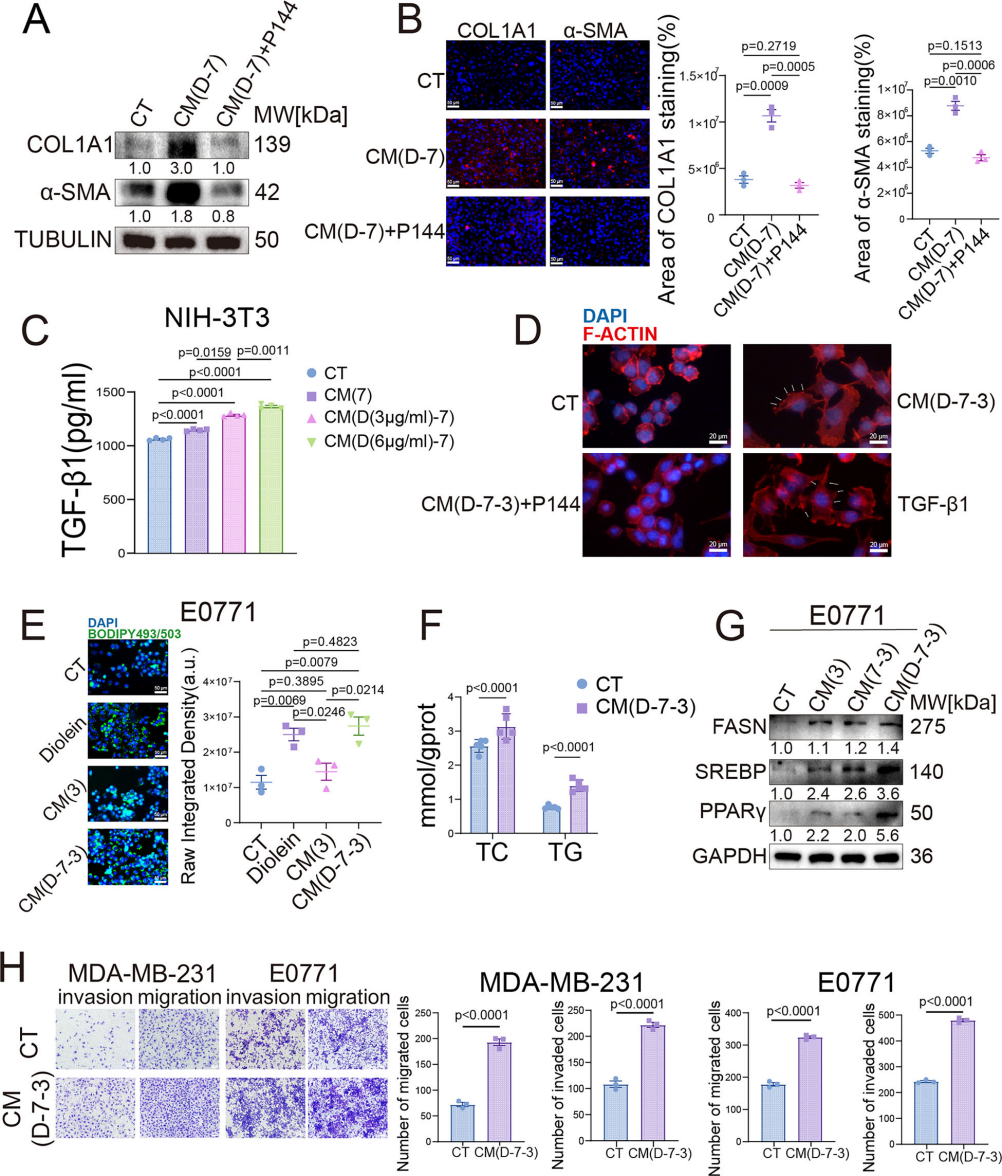
TGF-β1-driven tumor cell-CAFs crosstalk promotes TNBC progression. **A** Western blot analysis of COL1A1 and α-SMA expression in NIH-3T3 cells treated with control, conditioned medium from Diolein-treated E0771 cells (CM(D-7)), or CM(D-7) supplemented with P144 (TGF-β1 inhibitor). **B** Immunofluorescence staining and quantitative analysis of COL1A1 and α-SMA in NIH-3T3 cells under the same treatment conditions as in (**A**). Scale bar, 50μm. **C** TGF-β1 secretion levels measured by ELISA in NIH-3T3 cells cultured with different conditioned media. **D** Phalloidin staining of E0771 cells treated with control, conditioned medium from NIH-3T3 exposed to CM(D-7) (CM(D-7-3)), CM(D-7-3) + P144, or recombinant TGF-β1. Scale bar, 20μm. **E** BODIPY staining and quantitative analysis of lipid droplets in E0771 cells treated with control, Diolein, conditioned medium from NIH-3T3 (CM(3)), or CM(D-7-3). Scale bar, 50 μm. **F** Measurement of triglyceride and cholesterol levels in E0771 cells treated with control or CM(D-7-3). **G** Western blot analysis of lipid metabolism markers (FASN, SREBP, PPARγ) in E0771 cells treated with control, CM(3), conditioned medium from E0771-exposed NIH-3T3 (CM(7-3)), or CM(D-7-3). **H** Transwell invasion and migration assays with quantitative results in MDA-MB-231 and E0771 cells treated with control or CM(D-7-3). Scale bar, 100 μm. Data are presented as mean ± SEM.

Next, we sought to identify the specific soluble factor responsible for CAFs activation. Based on the critical role of TGF-β1 in fibroblast activation and stroma remodeling, we focused on this cytokine as the prime candidate mediating this intercellular crosstalk. Western blot analysis showed significantly increased expression of CAFs markers COL1A1 and α-SMA, an effect completely reversed by the TGF-β1 inhibitor P144 diammonium (Fig. 5A). IF staining further confirmed this phenomenon (Fig. 5B). ELISA revealed that DAG stimulates TGF-β1 secretion from tumor cells in a concentration-dependent manner (Fig. 5C). Functionally, TGF-β1 directly induced the formation of invadopodia in TNBC cells. CM from DAG-stimulated tumor cells was applied to pre-treat CAFs. Subsequently, the resulting CAFs-CM triggered invadopodia formation—a process blocked by TGF-β1 inhibitor (Figs. 5D, and S8A, B). This evidence suggests that DAG drives CAFs activation by regulating TGF-β1 secretion from tumor cells.

Previous studies have reported that CAFs can positively regulate tumor lipid metabolism [33, 34]. Consistently, BODIPY neutral lipid staining showed that CM(D-7-3) elevated neutral lipid levels in E0771 cells, mimicking the effect of DAG itself (Fig. 5E and S8C). Biochemical quantification further confirmed a concomitant increase in intracellular TAG and cholesterol content (Fig. 5F). Moreover, CM(D-7-3) significantly upregulated the protein levels of core lipid metabolism regulators, including FASN, SREBP, and PPARγ (Fig. 5G). Functionally, transwell assays demonstrated that CM(D-7-3) also markedly enhanced the migration and invasion capabilities of E0771 cells (Fig. 5H). These findings suggest that activated CAFs feedback-regulate tumor cells via factors such as TGF-β1, driving lipid metabolic reprogramming and synergistically promoting malignant phenotypic evolution.

### The DAG/PKC/CREB1/TGF-β1 axis drives TNBC tumor SWE stiffness and malignant progression via lipid metabolic reprogramming in vivo

**Fig. 6:**
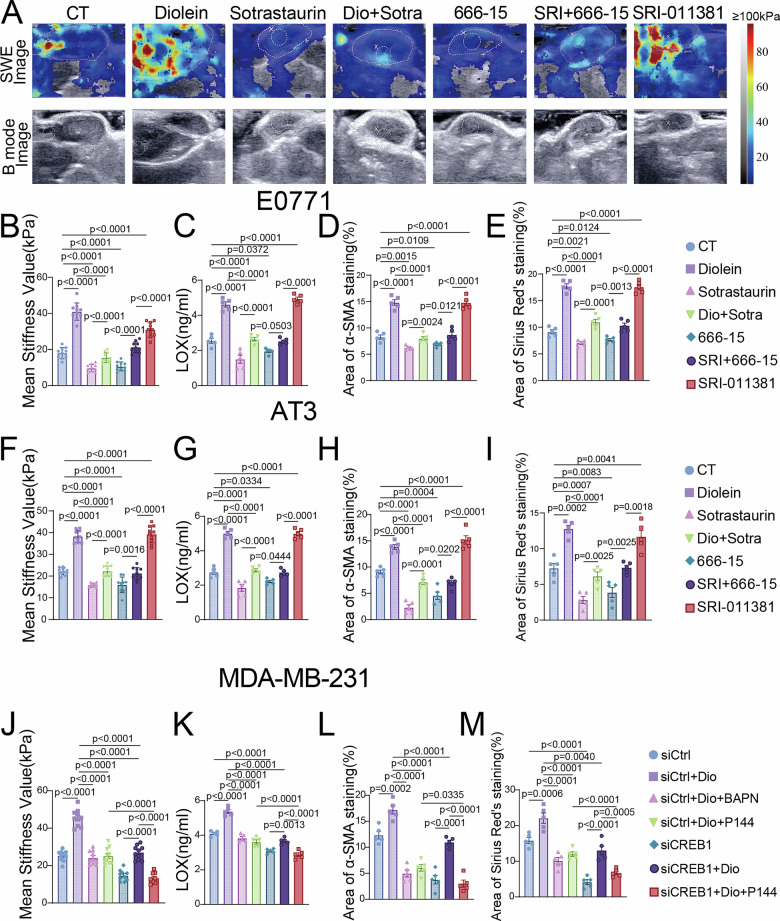
The DAG/PKC/CREB1/TGF-β1 axis drives TNBC tumor SWE stiffness and malignant progression via lipid metabolic reprogramming in vivo. **A** SWE and B-mode ultrasound images of tumors from the E0771 model. (*n* = 10 per group). Quantitative analysis of SWE-based tumor stiffness (**B**), LOX levels (measured by ELISA) (**C**), α-SMA IHC (**D**) and Sirius Red staining (**E**) in tumor tissues from the E0771 model. Quantitative analysis of SWE-based tumor stiffness (**F**), LOX levels (measured by ELISA) (**G**), α-SMA IHC (**H**) and Sirius Red staining (**I**) in tumor tissues from the AT3 model. Quantitative analysis of SWE-based tumor stiffness (**J**), LOX levels (measured by ELISA) (**K**), α-SMA IHC (**L**) and Sirius Red staining (**M**) in tumor tissues from the MDA-MB-231 model. Data are presented as mean ± SEM.

Next, we evaluated the effectiveness of the DAG/PKC/CREB1/TGF-β1 axis in altering TNBC SWE stiffness and malignant progression in vivo using two orthotopic TNBC models (E0771 and AT3). In the E0771 model, Diolein treatment or TGF-β1 activation significantly increased tumor SWE stiffness (Fig. 6A, B). Moreover, these interventions promoted tumor growth, as evidenced by increased tumor volume (Fig. S9A, B). Multiple histological and ELISA analyses were employed to assess TME fibrosis. Sirius Red and Masson’s etrichrom staining, combined with IHC for α-SMA, IF for COL1A1, and quantitative measurement of LOX expression, collectively revealed that Diolein or TGF-β1 activation markedly enhanced collagen deposition, fibroblast activation, and overall fibrosis within tumors (Figs. 6C–E and S9C–E). Conversely, treatment with the PKC inhibitor or CREB1 inhibitor resulted in a reduction of these fibrotic features, as well as in SWE stiffness and tumor cell proliferation. Similar trends were also observed in the AT3 orthotopic model (Figs. 6F–I and S10A–C).

To further validate the axis and assess the therapeutic potential of targeting key downstream nodes, we established an orthotopic model using MDA-MB-231 cells treated with a combination of CREB1 knockdown and pharmacologic inhibitors. We utilized ISC-modified siCREB1 (ISC-siCREB1) to achieve stable and efficient gene knockdown. Three distinct ISC-siCREB1 sequences were designed for in vivo use. Based on ImageJ-mediated quantification, ISC-siCREB1-3 demonstrated the highest knockdown efficiency and was therefore selected for subsequent studies (Fig. S11A). CREB1 knockdown effectively reversed Diolein-induced malignant phenotypes, including tumor growth (Fig. S11B), increased SWE stiffness (Figs. 6J and S11C), and extracellular matrix remodeling—as evaluated by LOX expression and collagen deposition (Figs. 6K–M and S111D). Furthermore, siCREB1 attenuated the activation of key downstream signaling molecules (Fig. S11E). Lipidomics sequencing further demonstrated that Diolein treatment specifically elevated intratumoral DAG and TAG content and significantly enriched the adipocytokine signaling pathway (Fig. S12A–C). Collectively, these data substantiate that the DAG/PKC/CREB1/TGF-β1 axis serves as a central mechanism driving TNBC tumor stiffness and progression by reprogramming lipid metabolism.

### Tumor cell-CAFs feedforward circuit drives TNBC stiffness, fibrosis and metastasis in vivo

**Fig. 7:**
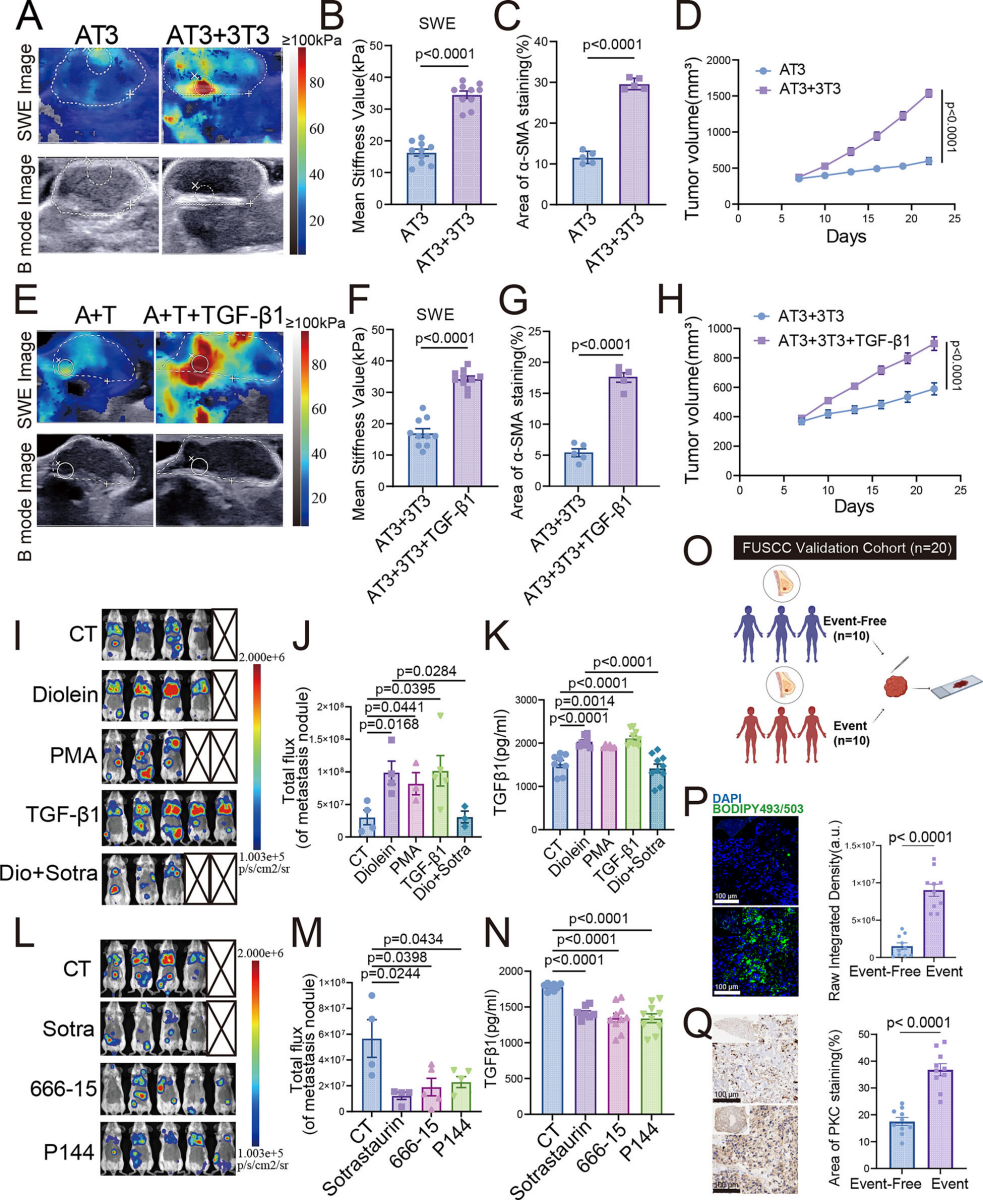
Tumor cell-CAFs feedforward circuit drives TNBC stiffness, fibrosis and metastasis In vivo. Nude mice were orthotopically injected into the mammary fat pad with AT3 cells alone or together with NIH-3T3 cells: **A** SWE and B-mode ultrasound images of tumors. **B** Quantitative analysis of SWE-based tumor stiffness. **C** Quantitative analysis of α-SMA IF in tumor tissues. **D** Tumor growth curves. Nude mice were injected with AT3 + NIH-3T3 cells with or without recombinant TGF-β1 cytokine: **E** SWE and B-mode ultrasound images of tumors. **F** Quantitative analysis of SWE stiffness. **G** Quantitative analysis of α-SMA IF. **H** Tumor growth curves. Mice were intravenously injected with 4T1-luc cells, subsequently treated with control, Diolein, PMA, TGF-β1, or Diolein + sotrastaurin: **I** Thermal imaging 10 days post-injection. **J** Quantification of metastatic signal by thermal imaging. **K** Serum TGF-β1 levels measured by ELISA. Mice were intravenously injected with 4T1-luc cells and subsequently treated with control, sotrastaurin, 666-15, or P144: **L** Thermal imaging 10 days post-injection. **M** Quantification of metastatic burden. **N** Serum TGF-β1 levels measured by ELISA. Comparative analysis of tissue samples from event(recurrent/deceased) TNBC patients versus event-free(non-recurrent) TNBC patients (*n* = 10 per group) in the FUSCC validation cohort: **O** Schematic illustration of the experimental design: TNBC tumor tissues from both patient groups were collected for subsequent staining. **P** BODIPY staining and quantitative analysis of lipid droplets. Scale bar, 100 μm. **Q** Representative IHC images and quantitative analysis of PKC expression. Scale bar, 100 μm. Data are presented as mean ± SEM.

In the TNBC orthotopic model, combining NIH-3T3 stromal cells with AT3 tumor cells increased tumor SWE stiffness versus AT3 cells alone (Fig. 7A, B). It also enhanced collagen deposition, fibroblast activation, and overall fibrosis (Figs. 7C and S13A–E). Concurrently, significant increases in tumor volume and weight were observed (Figs. 7D and S13F, G). Further investigation revealed that adding recombinant TGF-β1 cytokine to the AT3/NIH-3T3 co-implantation model elevated tumor SWE stiffness (Fig. 7E, F), intensified fibrotic deposition (Figs. 7G and S14A–E), and enhanced tumor growth (Figs. 7H and S14F, G).

To quantitatively link the observed mechanical changes to the fibrotic microenvironment, we performed correlation analyses across all in vivo samples. SWE stiffness values showed significant positive correlations with quantitative assessments of Sirius Red staining, COL1A1 IF intensity, and LOX expression levels (Fig. S15A–C), providing direct statistical evidence that increased tumor stiffness is closely associated with a fibrotic stromal response.

In conclusion, tumor cells and CAFs form a TGF-β1-mediated positive feedback loop, synergistically amplifying the effects of the DAG/PKC/CREB1/TGF-β1 pathway and collectively driving increased SWE stiffness and malignant progression in TNBC.

### Validation of the DAG/PKC/CREB1/TGF-β1 axis in promoting metastasis In vivo

To clarify the regulatory role of the DAG/PKC/CREB1/TGF-β1 axis on metastasis, we established a 4T1-luc-GFP metastasis model. Bioluminescence imaging (BLI) showed that DAG treatment, PKC activation, and TGF-β1 intervention all significantly promoted the formation of metastatic foci (Fig. 7I, J) and were accompanied by increased serum TGF-β1 levels (Fig. 7K). Conversely, inhibiting PKC, CREB1, or TGF-β1 effectively suppressed metastatic spread (Fig. 7L, M) and concurrently reduced serum TGF-β1 concentration (Fig. 7N). Finally, we performed staining on TNBC tissues from recurrent or deceased patients in the FUSCC validation cohort (Fig. 7O). It revealed significantly higher lipid levels by BODIPY staining (Fig. 7P), along with elevated expression of PKC activation and fibrosis (Figs. 7Q and S16A, B), compared to tissues from non-recurrent survivors. Collectively, these in vivo and clinical findings demonstrate that the DAG/PKC/CREB1/TGF-β1 axis drives metastatic progression, and its activation in human TNBC tissues is associated with poor clinical outcomes.

## Discussion

**Fig. 8:**
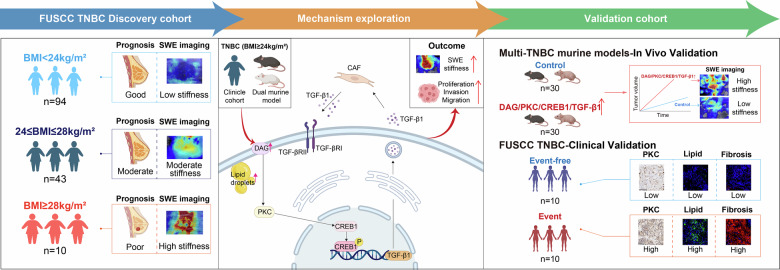
Schematic overview of the study design and key findings. Part 1: FUSCC TNBC Discovery Cohort. Illustrates the association between body mass index (BMI), patient prognosis, and shear wave elastography (SWE) stiffness in the FUSCC TNBC cohort. Higher BMI correlates with higher SWE stiffness and poorer prognosis. Part 2: Mechanism Exploration. In tumor cells of obese TNBC patients, elevated intracellular diacylglycerol (DAG) levels activate the PKC/CREB1 signaling pathway, resulting in TGF-β1 upregulation and secretion. Secreted TGF-β1 activates cancer-associated fibroblasts (CAFs), which further secrete TGF-β1, forming a positive feedback loop. This bidirectional crosstalk between tumor cells and CAFs enhances stromal stiffness (quantified by SWE) and drives tumor proliferation, invasion, and migration. Part 3: Validation Cohort. Validation of the proposed mechanism through in vivo mouse models and clinical confirmation in the FUSCC TNBC validation cohort. The mouse model demonstrates the role of the DAG/PKC/CREB1/TGF-β1 axis in promoting stromal stiffness and tumor progression, while clinical data reinforce the association between obesity, stromal stiffness, and aggressive TNBC phenotypes.

In this study, we demonstrate that high BMI in TNBC patients promotes lipid metabolic reprogramming characterized by DAG accumulation, activating the DAG/PKC/CREB1 signaling axis. This upregulates TGF-β1 expression, driving increased SWE stiffness, tissue fibrosis, and tumor proliferation/invasion. Notably, elevated TGF-β1 secretion into the extracellular matrix significantly activates CAFs. Activated CAFs further secrete TGF-β1 to stimulate tumor cells, establishing a self-sustaining positive feedback loop that maintains SWE stiffness elevation, fibrosis, and malignant progression (Fig. 8). This study illuminates a novel and mechanistically detailed pathway—the DAG/PKC/CREB1/TGF-β1 signaling axis—that functionally connects obese lipid metabolism, SWE stiffness, and TNBC malignant progression. Crucially, we identified a TGF-β1-mediated positive feedback loop between tumor cells and CAFs that sustains and amplifies this vicious cycle, offering a new biological rationale for the poor prognosis of obese TNBC patients and presenting a compelling target for therapeutic intervention.

The high mortality of TNBC arises from limited therapeutic options and lack of effective targets [27]. Obesity increasingly influences incidence and mortality across multiple malignancies. Clinical observations indicate obese patients often present with larger tumors, higher lymph node metastasis rates, and reduced survival. The obesity-cancer link is particularly pronounced due to adipose-rich mammary microenvironments in BC. Excessive adipose tissue triggers local changes (e.g., dysregulated lipid metabolism, hormonal imbalances, chronic inflammation) and systemic alterations that fuel tumor progression [9]. Studies elucidating the relationship between lipid metabolism and ultrasonic imaging characteristics remain limited. For BI-RADS 4a breast lesions, incorporating the fat-to-lesion ratio measured by SWE into standard B-mode ultrasound reduced benign biopsies by nearly half [35]. Our clinical cohort analysis further confirmed that BMI is significantly associated with long-term prognosis in TNBC patients, while also demonstrating a clear clinical correlation between BMI and ultrasonic features (e.g., SWE stiffness).

Linking imaging features to histopathology carries clinical significance. Current research correlates SWE parameters with neoadjuvant therapy response [36], residual tumor assessment [37], and axillary lymph node staging [38]. Tumor invasiveness and histopathological subtypes independently influence SWE values [39]. Despite confirmed imaging-pathology relationships, underlying mechanisms remain elusive. Our data reveals that high BMI TNBC patients exhibit significantly elevated SWE stiffness and reduced grayscale values. Crucially, longitudinal murine studies demonstrated progressively diverging SWE stiffness between HFD and CD groups without grayscale changes—suggesting that dysregulated lipid metabolism preferentially modulates mechanical properties (e.g., stiffness). Based on this key evidence, we targeted the regulatory mechanisms linking hyperactivated lipid metabolism to SWE stiffness for subsequent investigation. Integrated lipidomics-transcriptomics analysis identified DAG and its downstream target PKC as mediators of tissue fibrosis, providing critical mechanistic insight into SWE stiffness elevation.

As a ubiquitous membrane component, DAG facilitates vital biological processes, including signal transduction. The DAG/PKC signaling axis critically drives BC malignancy: PKC induces EMT and enhances invasiveness [40–42], while PKC-overexpressing patients may benefit from Sotrastaurin [43]. Our functional experiments confirm that PKC-dependent DAG signaling remodels lipid metabolism, drives EMT, and accelerates invasion/migration—supporting PKC targeting as a viable TNBC treatment approach.

PKC phosphorylates CREB to promote TNBC proliferation, invasion, and tumor growth, while disrupting CREB-coactivator interactions suppresses bone metastasis [44]. As an oncogenic transcription factor, aberrant CREB1 activation drives proliferation and invasion in multiple malignancies [45–47], though its role in TNBC remains underexplored. This study reveals that CREB1, as the core downstream effector of the DAG/PKC signaling axis, remodels TNBC malignant phenotypic networks (e.g., EMT and metabolic reprogramming), providing novel experimental evidence for its therapeutic targeting in TNBC and deepening mechanistic insights into this signaling network.

The TGF-β signaling pathway exhibits sustained activation within the TNBC microenvironment, and its dysregulation is closely associated with TNBC development and progression [48]. Integrated omics revealed TGF-β pathway enrichment during lipid metabolism-driven metastasis. This pleiotropic cytokine promotes angiogenesis, immunosuppression, and EMT-driven malignancy in TNBC [49]. Previous studies have established that TGF-β1 significantly regulates morphological changes, migratory capacity, and EMT core marker expression in MCF-7 cells, while also modulating invasive properties and cytokine secretion profiles in MDA-MB-231 cells [50]. Importantly, PKC inhibition reduces TGF-β1 secretion [51], aligning with our proposed axis. Through JASPAR-based predictions and experimental validation, we established TGF-β1 as the terminal effector downstream of DAG/PKC/CREB1. Functional studies confirmed TGF-β1 remodels extracellular matrices to drive fibrosis and metastasis. This study delineates the mechanism by which TGF-β1, as the core downstream effector of the DAG/PKC/CREB1 signaling axis, drives malignant progression in TNBC.

Prior work shows CAFs proportion and functional states co-regulate TNBC SWE stiffness [16–18]. Our multi-omics analysis confirmed that hyperactivated lipid metabolism significantly enriches fibrosis-associated signaling pathways, while PKC inhibition directly downregulates TGF-β1 expression and suppresses CAFs migration. Therefore, we investigated the impact of the DAG/PKC/CREB1/TGF-β1 axis on SWE stiffness while simultaneously dissecting tumor cell-CAFs crosstalk in TNBC. Given the established role of CAFs in microenvironment deterioration [52] and metastasis promotion [53], we investigated reciprocal tumor-CAFs interactions. Studies have also shown that TGF-β1 can maintain the state of CAFs [54], which is consistent with our research results. We discovered that TGF-β1, secreted by tumor cells under conditions of high lipid metabolism, acts in a paracrine manner to activate CAFs. Once activated, these CAFs not only contribute to ECM remodeling but also secrete TGF-β1 themselves. This creates a self-sustaining positive feedback loop: CAF-derived TGF-β1 reciprocally stimulates tumor cells, further amplifying the entire DAG/PKC/CREB1/TGF-β1 axis and its oncogenic outputs. This loop provides a mechanistic explanation for how the obese microenvironment can initiate a persistent and escalating cycle of fibrosis and progression that may continue even after the initial metabolic insult. Furthermore, our data suggest that activated CAFs can feed back to enhance tumor cell lipid metabolism, although the exact mediators of this effect warrant future investigation. This bidirectional crosstalk, rather than unidirectional stimulation, establishes a co-evolutionary niche that is central to maintaining the stiff, fibrotic, and aggressive TNBC phenotype.

This study elucidated the complete signaling pathway from metabolic perturbation to the radiomic phenotype. Our work transcends conventional mechanistic exploration by offering dual layers of innovation: (1) Theoretical innovation: We uncover a novel signaling axis that functionally links metabolic reprogramming to the biomechanical properties of the TME, providing a new biological rationale for interpreting SWE images. (2) Translational innovation: More importantly, we elevate SWE stiffness from a diagnostic marker to a potential prognostic indicator capable of inferring real-time oncogenic pathway activity. This provides a novel strategy for assessing metastatic propensity and lays the groundwork for “image-guided” combination therapy. For instance, high-BMI patients with elevated SWE stiffness could be prioritized for clinical trials involving PKC inhibitors (e.g., Sotrastaurin [43]), TGF-β inhibitors, or their combination with standard chemotherapy.

In addition, our study has some limitations. The relatively small cohort size necessitates larger follow-up studies to validate these findings. Additionally, the specific mechanisms underlying CAFs-induced lipid metabolic reprogramming in tumor cells remain unexplored, warranting further investigation in future research.

In summary, our work illuminates how DAG-driven metabolic reprogramming dynamically regulates TNBC SWE stiffness. We mechanistically link lipid metabolism, imaging biomarkers, and pathology to the activation of the DAG/PKC/CREB1/TGF-β1 signaling axis—driving stiffness, fibrosis, proliferation, and invasion. Crucially, we identify bidirectional TGF-β1-mediated tumor-CAFs crosstalk (not unidirectional stimulation) as the engine sustaining fibrosis. This axis represents a novel therapeutic target for precision breast cancer therapy. Clinically, high BMI patients could be stratified using serum DAG levels combined with SWE stiffness screening for prioritized targeted interventions.

## Materials and methods

### Cell lines and reagents

#### Cell lines and culture conditions

Human BC cell line MDA-MB-231 and murine BC cell lines E0771, 4T1, and AT3 were purchased from the American Type Culture Collection (ATCC). 4T1-luc and NIH-3T3 cells were obtained from QuiCell (Shanghai, China). MDA-MB-231, E0771, AT3, and NIH-3T3 cells were cultured in DMEM supplemented with 10% FBS. 4T1 and 4T1-luc cells were maintained in RPMI-1640 supplemented with 10% FBS. All media contained 1% penicillin-streptomycin. All cells were incubated at 37 °C in a 5% CO₂ incubator and were passaged at 80–90% confluence using 0.25% trypsin-EDTA. All incubations were performed with cells at passages 1–5.

#### Chemical reagents and agonists/inhibitors

The following compounds were used in this study: Diolein (GC45432, GLPBIO, Montclair, CA, USA), Sotrastaurin (HY-10343, MCE, New Jersey, USA), 666-15 (HY-101120, MCE, New Jersey, USA), SRI-011381 hydrochloride (HY-100347A, MCE, New Jersey, USA), PMA (HY-18739, MCE, New Jersey, USA), P144 (HY-P0118, MCE, New Jersey, USA), BAPN (HY-Y1750, MCE, New Jersey, USA).

**Lipid metabolomics detection**

To analyze the effect of hyperactivated lipid metabolism on lipid accumulation, mammary tumors from HFD and CD mice (*n* = 6 per group) were used.

#### Sample preparation

Lipids were extracted using a modified protocol. An appropriate amount of sample was homogenized with two steel beads in 300 µL methanol‑water (1:1, v/v) containing internal standards (GCA‑C13, CA‑D4) after precooling at –20 °C for 2 min, using a tissue grinder (60 Hz, 2 min). Subsequently, 300 µL chloroform was added; the mixture was vortexed for 30 s, ultrasonicated for 10 min, and held at –20 °C for 20 min. After centrifugation (10 min, 13,000 rpm, 4 °C), about 200 µL of the lower chloroform layer was collected. The residue was re‑extracted with 300 µL chloroform‑methanol (2:1, v/v) containing BHT, followed by vortexing (30 s), ice‑water‑bath ultrasonication (10 min), incubation (–20 °C, 20 min) and centrifugation under the same conditions; about 100 µL of the lower layer was combined with the first extract and dried. The residue was reconstituted in 200 µL isopropanol‑methanol (1:1, v/v) by vortexing (30 s) and ultrasonication (3 min), mixed with 20 µL of a mixed isotope‑labeled internal standard mix (see Appendix), transferred to a 1.5 mL tube, and stored at –20 °C for 2 h. After a final centrifugation (10 min, 13,000 rpm, 4 °C), 150 µL of supernatant was taken for LC‑MS analysis. Quality control (QC) samples were prepared by pooling equal volumes of all sample extracts.

#### LC-MS/MS analysis

The metabolomic data analysis was performed by Shanghai Luming Biological Technology Co., LTD (Shanghai, China). The LC system was performed using an ExionLC™ System consisting of a binary high-pressure mixing gradient pump with degasser, a thermostated autosampler, and a column oven, and the MS was QTRAP 6500+ (AB Sciex, USA) equipped with an IonDrive™ Turbo V source. Finally, the optimization conditions are as follows: The temperature of the autosampler and oven were set at 4 °C and 55 °C, respectively. The sample injection volume was 5 μl. Eluents consisted of 0.1% formic acid and 10 mM ammonium formate in 6:4 acetonitrile/water (eluent A) and 1:9 acetonitrile/isopropanol contained 0.1% formic acid and 10 mM ammonium formate (eluent B). The flow rate was set at 0.35 mL/min. A 20 min elution gradient with an UPLC HSS T3 (1.8um, 2.1x100mm, Waters) column was performed as follows: during the first 1.5 min, eluent composition was set at 0% B, which was linearly changed to 55% B at 5 min, then 60%B at 10 min, 70% at 13 min and 90% at 15 min, in the next 16 min B was increased to 100% and kept for 2 min. Finally, the initial conditions were recovered and maintained for 2 min for column conditioning. The MS method was performed in the negative/positive-ion mode, working in the time-scheduled MRM method, respectively. The source condition was as follows: Curtain gas was 40 psi, CAD was medium, the IS was -4500V/ + 5500 V and the Gas1 and Gas2 was 50 psi and 55 psi.

#### Data processing and statistical analysis

Raw LC-MS data were processed using Progenesis QI software (v2.3, Nonlinear Dynamics, UK) for baseline correction, peak detection, peak area integration, retention time alignment, and normalization. Specifically, normalization was performed by median scaling to the total ion intensity of all samples. Mass accuracy tolerances were set to 5 ppm for precursors and 10 ppm for products, with a product ion threshold of 5%. Metabolite identification was performed by matching precise m/z values, MS/MS fragments, and isotopic patterns against the Human Metabolome Database (HMDB), Lipidmaps (v2.3), Metlin, and a custom in-house database.

Prior to statistical analysis, features with more than 50% missing values (zero intensity) within any experimental group were excluded. Remaining zero values were imputed as half of the minimum positive value for the corresponding variable. Further quality filtering was performed by removing compounds with an identification confidence score below 36 (out of 60). A combined data matrix was constructed using features detected in both positive and negative ionization modes. Multivariate statistical analyses were performed in the R environment. Principal Component Analysis (PCA) was first applied to assess overall data structure, detect outliers, and evaluate process stability. To improve group separation and identify metabolites contributing most to class discrimination, Orthogonal Partial Least Squares-Discriminant Analysis (O-PLS-DA) and Partial Least Squares-Discriminant Analysis (PLS-DA) were employed. Model robustness was evaluated through 7-fold cross-validation and 200 rounds of response permutation testing. Metabolites with a Variable Importance in Projection (VIP) score exceeding 1.0 from the O-PLS-DA model were considered relevant for group separation. Significance was further verified using a two-tailed Student’s *t* test, with a threshold of *p* < 0.05. This combined criterion (VIP > 1.0 and *p* < 0.05) was applied to balance both the contribution of variables to group discrimination and their statistical significance, thereby controlling for false positive discoveries. Differentially abundant metabolites meeting both criteria were subsequently subjected to KEGG pathway enrichment analysis (http://www.genome.jp/kegg/).

### RNA sequencing

#### RNA sequencing and differential expression analysis

Library sequencing was carried out on the Illumina NovaSeq 6000 platform, producing 150 bp paired-end reads. An average of 49.207 million raw reads per sample was generated. Raw FASTQ files were quality-filtered using fastp to remove low-quality sequences, yielding approximately 45.679 million clean reads, which were aligned to the NCBI_GRCm39 using HISAT2. Gene expression levels were quantified as FPKM, and raw read counts were obtained with HTSeq-count. Following alignment and quantification, protein-coding genes with zero counts across all samples were filtered out prior to downstream analysis. Differential gene expression analysis was performed using DESeq2, which internally normalizes counts using the median of ratios method. Genes with |log₂(fold change)| > 1 and P-value (Benjamini-Hochberg) < 0.05 were defined as significantly differentially expressed genes (DEGs). No batch correction was applied as all samples were processed and sequenced simultaneously. Principal Component Analysis (PCA) was conducted in R (v 4.3.0) to assess sample reproducibility and group segregation. A heatmap of hierarchical clustering of DEGs was generated to visualize expression patterns across groups and samples.

Functional enrichment analysis of DEGs was performed based on the hypergeometric distribution algorithm. GO, KEGG pathway, Reactome, and WikiPathways analyses were conducted to identify significantly enriched terms, with *P* value < 0.05 set as the significance threshold. Results were visualized via bar plots, chord diagrams, and bubble plots using R (v 4.3.0). Gene Set Enrichment Analysis (GSEA) was implemented using GSEA software against predefined gene sets derived from GO and KEGG pathway databases. Predefined gene sets were evaluated by ranking all genes based on differential expression metrics between groups, followed by 1000 permutations to test whether specific gene sets were enriched at the extreme ends of the ranked list. Gene sets with fewer than 5 or more than 500 genes were excluded from the GSEA to avoid overly narrow or broad categories. Significantly enriched gene sets were identified using a combination of *p*-value < 0.05 and |normalized enrichment score (NES)| > 1.0.

### RNA interference in vitro and in vivo

#### In vitro knockdown in cell lines

RNA interference to knock down CREB1 in cells was performed using jetPRIME® transfection reagent (101000046, Polyplus, Illkirch, France) according to the manufacturer’s instructions. Briefly, the transfection reagent and siRNA (A01001, GenePharma, Suzhou, China) were mixed in the provided buffer, incubated for 10 min at room temperature, and then added to cells in complete culture medium. The siRNA sequences used were as follows: siCtrl: Sense 5‘-UUCUCCGAACGUGUCACGUTT-3’, Antisense 5‘-ACGUGACACGUUCGGAGAATT-3’; siCREB1-1: Sense 5‘-CUGCCACAAAUCAGAUUAATT-3’, Antisense 5‘-UUAAUCUGAUUUGUGGCAGTT-3’; siCREB1-2: Sense 5‘-CCAGCAACCAAGUUGUUGUTT-3’, Antisense 5‘-ACAACAACUUGGUUGCUGGTT-3’; siCREB1-3: Sense 5‘-GUCUCCACAAGUCCAAACATT-3’, Antisense 5‘-UGUUUGGACUUGUGGAGACTT-3’ cells were harvested 48 h post-transfection for subsequent functional assays (CCK-8, colony formation, transwell) or total protein extraction.

#### In vivo knockdown in mouse models

For in vivo knockdown, the CREB1-targeting siRNA (ISC-siCREB1, A17064, GenePharma, Suzhou, China) or a non-targeting control siRNA (ISC-NC) was complexed with the in vivo transfection reagent invivo siRNA-mate (G04029, GenePharma, Suzhou, China) according to the manufacturer’s protocol. The siRNA/polyplex mixture was administered to mice via intraperitoneal injection at a dose of 5 µg siRNA per gram of mouse body weight. Injections were performed once per week. The ISC-siRNA sequences used were as follows: ISC-siCtrl: Sense 5‘-CACUUACGCUGAGUACUUCGA-3’, Antisense 5‘-UCGAAGUACUCAGCGUAAGUGAU-3’; ISC-siCREB1-1: Sense 5‘-ACAGGGCCUGCAAACAUUA-3’, Antisense 5‘-UAAUGUUUGCAGGCCCUGUAC-3’; ISC-siCREB1-2: Sense 5‘-GCCACAGAUUGCCACAUUA-3’, Antisense 5‘-UAAUGUGGCAAUCUGUGGCUG-3’; ISC-siCREB1-3: Sense 5‘-AGCACCCACUAGCACUAUU-3’, Antisense 5‘-AAUAGUGCUAGUGGGUGCUGU-3’. Tumors or relevant tissues were harvested for downstream analysis at the indicated experimental endpoints.

### Tumor SWE imaging

#### Tumor model standardization & SWE image acquisition

Tumor SWE imaging was performed one day before the endpoint using an ultrasonic diagnostic apparatus equipped with a linear array probe (model: Pen/Med) in SWE mode (M 1/Med). All tumors were generated under a standardized protocol to ensure consistent size and depth from the skin surface. During imaging, parameters were set as follows: sampling frequency S 5/O 75%, gain G 70%, penetration mode, velocity scale 0–100 kPa, with the focus fixed at the horizontal plane of the tumor center. All scans were conducted by experienced ultrasonographers (å 5 years) to ensure acquisition consistency.

#### SWE stiffness quantification

Quantitative analysis was performed by placing three spherical 1-mm diameter regions of interest (ROIs) within the stiffest tumor areas on the elastography map; the average value was calculated. To control for depth-related artifacts, ROIs of identical size were also placed in adjacent normal tissue at the same depth as an internal reference (these reference values were not included in the final statistical analysis).

### Statistical analysis

Data are presented as mean ± standard error of the mean (SEM), as stated in the figure legends. The SEM is used here to visualize the precision of the mean estimate and to aid in the visual assessment of differences and trends between group means. The normality of all data distributions was assessed using the Shapiro-Wilk test, and the equality of variances was verified using the Brown-Forsythe test. Based on these assessments, the appropriate statistical tests were selected. For comparisons between two groups of normally distributed data, an unpaired or paired Student’s t-test was used. For non-normally distributed data, the Mann-Whitney U test (unpaired) or Wilcoxon signed-rank test (paired) was applied. For comparisons among three or more groups, one-way or two-way analysis of variance (ANOVA) was used for parametric data, followed by an appropriate post-hoc test for multiple comparisons: Tukey’s test was used for all pairwise comparisons; Dunnett’s test was used for comparisons against a single control group. The non-parametric Kruskal-Wallis test with Dunn’s post-hoc correction was used if the data did not meet ANOVA assumptions. Correlation analysis was performed using Pearson’s test for normally distributed data or Spearman’s rank correlation test for non-normally distributed data. Comparisons of categorical variables were performed using the Chi-square test or Fisher’s exact test, as appropriate.

All statistical analyses were performed using GraphPad Prism (v 9.0) and R (v 4.3.0). A two-tailed *P* value < 0.05 was considered statistically significant. All experiments were repeated at least three times to ensure reproducibility.

### Ethics approval and consent to participate

Tissue samples of TNBC patients were obtained from the tissue bank of FUSCC. All samples were pathologically diagnosed as TNBC. The use of clinical information and tissues was approved by the Ethics Committee of Fudan University Shanghai Cancer Center (approval number: 2509-ZZK-153). The animal studies have been approved by the Ethics Committee of Fudan University Shanghai Cancer Center (202503FD0012).

## Supplementary information

Supplementary Material Legends

Supplementary Materials and Methods

Original Western blot images

Checklist

Supplementary Figure S1

Supplementary Figure S2

Supplementary Figure S3

Supplementary Figure S4

Supplementary Figure S5

Supplementary Figure S6

Supplementary Figure S7

Supplementary Figure S8

Supplementary Figure S9

Supplementary Figure S10

Supplementary Figure S11

Supplementary Figure S12

Supplementary Figure S13

Supplementary Figure S14

Supplementary Figure S15

Supplementary Figure S16

Supplementary Table S1

## Data Availability

The RNA sequencing data for our study have been deposited into the Genome Sequence Archive (GSA) database under accession codes PRJNA1308990 (https://www.ncbi.nlm.nih.gov/bioproject/PRJNA1308990) and PRJNA1310775 (https://www.ncbi.nlm.nih.gov/bioproject/PRJNA1310775). The raw LCMS data generated in this study have been deposited in the OMIX database (https://ngdc.cncb.ac.cn/omix/) under accession numbers OMIX011620, OMIX015284 and OMIX011628. These data are publicly accessible and available for download to facilitate further analysis and replication of our findings. All data generated or analysed during this study are included in this published article and its supplementary information files.
