# Supplementary material for: The DAG/PKC/CREB1/TGF-β1 axis drives shear-wave elastography stiffness and malignant progression in triple-negative breast cancer via lipid metabolic reprogramming
Source: Cell Death Dis. 2026 Mar 20;17(1):327. doi: 10.1038/s41419-026-08625-0 (PMC13039978; doi:10.1038/s41419-026-08625-0)
Supplement: Supplementary file 1 — Supplementary Material Legends [file 41419_2026_8625_MOESM1_ESM.docx]

**Supplementary Table 1. Complete antibody information**

**Supplementary Fig. 1 Association between BMI and ultrasonic grayscale values in the FUSCC TNBC cohort (n=147). A–C** Analyses based on the FUSCC TNBC cohort(n=147): **A** Correlation between BMI and grayscale values under B-mode ultrasound. **B** Comparison of grayscale values between deceased and surviving patients. **C** Comparison of SWE values between patients BMI≥24 kg/m² and <24 kg/m² based on the FUSCC TNBC cohort(n=147). **D** Comparison of grayscale values between patients with BMI≥24 kg/m² and <24 kg/m²(n=147). **E** Comparison of grayscale values between patients with BMI<24 kg/m², 28>BMI≥24 kg/m² and BMI≥28 kg/m² based on the FUSCC TNBC cohort(n=147). **F, G** Representative images(**F**) and quantitative analysis(**G**) of dual IF for COL1A1 (red) and Ki67 (green) in tumor tissues from patients with normal weight, overweight, and obesity (n=3 per group). Scale bar, 50 μm. Data are presented as mean ± SEM.

**Supplementary Fig. 2 Obesity promotes tumor progression and modulates ultrasonic characteristics in the E0771 TNBC mouse model. A** Schematic of the CD and HFD mouse models(n=10 per group). After 8 weeks of dietary induction, E0771 cells were injected orthotopically. Tumors were analyzed by ultrasound at 3 and 4 weeks post-injection, followed by WB, staining, and sequencing. **B** Body weight curves of CD and HFD mice. **C** Representative images of resected tumors. **D** SWE and grayscale ultrasound images of tumors at 3 weeks. **E, F** Comparison of grayscale values between CD and HFD groups at 3 (**E**) and 4 (**F**) weeks. **G, H** Grayscale value comparison in CD mice (**G**) and HFD mice (**H**) between 3 and 4 weeks. **I-M** Representative images(**I**) and quantitative analysis of Sirius Red staining (**J**) (scale bar, 100 μm), COL1A1 (**K**), α-SMA(**L**) IF (scale bar, 50 μm) and LOX levels (measured by ELISA) (**M**) in E0771 tumors between CD and HFD groups. Data are presented as mean ± SEM.

**Supplementary Fig. 3 Obesity promotes tumor progression and modulates ultrasonic characteristics in the 4T1 TNBC mouse model. A** Body weight curves of CD and HFD mice. **B** SWE and grayscale ultrasound images of tumors. **C, D** Comparison of SWE-based tumor stiffness (**C**) and grayscale values (**D**) between CD and HFD groups. **E-I** Representative images(**E**) and quantitative analysis of Sirius Red staining (**F**) (scale bar, 100 μm), COL1A1 (**G**) and α-SMA(**H**) IF (scale bar, 50 μm) and LOX levels (measured by ELISA) (**I**) in 4T1 tumors between CD and HFD groups. **J** Western blot analysis of lipid metabolism-related proteins (FASN, SREBP, PPARγ) in 4T1 tumors from CD and HFD mice. Data are presented as mean ± SEM.

**Supplementary Fig. 4 Lipidomic and transcriptomic profiling and pathway enrichment analysis of tumors from CD and HFD E0771 mice.** **A** PCA score plot of LC-MS/MS-based lipidomics data from tumors in CD and HFD groups. **B–E** GSEA enrichment plots showing significantly altered pathways: **B** Cell adhesion molecules, **C** Synthesis of IP3 and IP4 in the cytosol, **D**Elastic fibre formation, **E** Lung fibrosis.

**Supplementary Fig. 5 PKC-Dependent DAG Signaling Drives TNBC Metastasis.** **A** Representative BODIPY staining (green) of MDA-MB-231 cells treated with Diolein, with nuclei counterstained by DAPI (blue). Scale bar, 50 μm. **B** Colony formation assay of MDA-MB-231 and E0771 cells treated with Diolein and Sotrastaurin. **C** Wound healing assay of E0771 cells under Diolein and Sotrastaurin treatment. Scale bar, 100 μm. **D** Transwell migration and invasion assays of E0771 cells following Diolein and Sotrastaurin treatment. Scale bar, 100 μm.

**Supplementary Fig. 6 PKC inhibition suppresses TNBC cell malignant phenotypes potentially through regulating CREB1-mediated TGF-β1 transcription and fibroblast migration-related pathways. A** Schematic diagram predicting potential CREB1 binding sites (YBS) within the TGF-β1 promoter using the JASPAR database. **B** GSEA plot of the CREB1 target gene set from RNA-seq data of Sotrastaurin-treated MDA-MB-231 cells. **C** Reactome enrichment analysis of DEGs enrichment in RNA-seq data from Sotrastaurin-treated MDA-MB-231 cells; the pathways of interest are marked in red. **D**GSEA plot of the REGULATION OF FIBROBLAST MIGRATION pathway from RNA-seq data of Sotrastaurin-treated MDA-MB-231 cells. **E** Western blot analysis of CREB1 and phosphorylated CREB1 (p-CREB1) in MDA-MB-231 treated with siCREB1. **F**Western blot analysis of EMT-related markers in E0771 cells treated with 666-15 and SRI-011381 (TGF-β1 activator). **G-I** Colony formation assay(**G**), wound healing assay(**H**) and transwell invasion and migration assays(**I**) in MDA-MB-231 cells treated with siCREB1 and Diolein. Scale bars (**H, I**), 100μm. **J** Colony formation assay of MDA-MB-231 and E0771 cells treated with 666-15 or SRI-011381. **K, L**Wound healing assay of MDA-MB-231 cells(**K**) and E0771 cells(**L**) following treatment with 666-15 or SRI-011381. Scale bars, 100 μm. **M, N**Transwell migration and invasion assays of MDA-MB-231 cells(**M**) and E0771 cells(**N**) under 666-15 or SRI-011381 treatment. Scale bars, 100 μm.

**Supplementary Fig. 7 Lipidomics analysis excludes residual DAG as the cause of CM(D-7-3)-induced proliferation and invasion. A** Heatmap depicting the relative abundance of differentially altered DAG species by LC-MS/MS-based lipidomics across the Vehicle, CM(D-7-3), and CM(D-7) groups (n = 4 per group). Rows represent individual DAG species, and columns represent individual biological samples. **B** The log₂-transformed relative abundance of DAG (18:0/18:2) was determined by LC-MS/MS-based lipidomics across the Vehicle, CM(D-7-3), and CM(D-7) groups (n = 4 per group). **C-E** CCK-8 cell viability assay(**C**), colony formation assay(**D**) and wound healing assay(**E**) with quantitative results in MDA-MB-231 cells treated with vehicle, Diolein at various concentrations, or CM(D-7-3). Scale bar(**E**), 100μm. Data are presented as mean ± SEM.

**Supplementary Fig. 8 TGF-β1 signaling mediates stromal remodeling and lipid accumulation in TNBC cells under DAG-related stimulation. A**Phalloidin staining showing actin organization in MDA-MB-231 cells treated with recombinant TGF-β1. Scale bar, 20 μm. **B**Phalloidin staining of E0771 cells treated with TGF-β1, CM(D-7-3), or CM(D-7-3) plus the TGF-β inhibitor P144. Scale bar, 20 μm. **C**BODIPY staining of E0771 cells treated with Diolein, CM(3), or CM(D-7-3) to evaluate lipid droplet formation. Scale bar, 50 μm. CM(D-7-3): conditioned medium from NIH-3T3 cells activated by CM of DAG-pre-treated E0771 cells; CM(3): conditioned medium from untreated NIH-3T3 cells.

**Supplementary Fig. 9 Functional validation of the DAG/PKC/CREB1/TGF-β1 axis in the E0771 model. A** Tumor growth curves of E0771 mice in each treatment group. **B** Representative images of resected tumors. **C-E** Representative images and quantitative analysis of α-SMA IHC, Sirius Red staining, Masson’s trichrome staining(**D**) (scale bars, 100 μm) and COL1A1 IF(**E**) (scale bar, 50 μm) in E0771 tumors across treatment groups. Data are presented as mean ± SEM.

**Supplementary Fig. 10 Functional validation of the DAG/PKC/CREB1/TGF-β1 axis in the AT3 model. A** Tumor growth curves of AT3 mice in each treatment group. **B** Representative images of SWE, grayscale ultrasound images, α-SMA IHC, Sirius Red staining, Masson’s trichrome staining (scale bars, 100 μm) and COL1A1 IF (scale bar, 50 μm) in AT3 tumors across treatment groups. **C**Quantitative analysis of COL1A1 IF in AT3 tumors across treatment groups. Data are presented as mean ± SEM.

**Supplementary Fig. 11 Functional validation of the DAG/PKC/CREB1/TGF-β1 axis in the MDA-MB-231 model. A** Western blot analysis of CREB1 and p-CREB1 in MDA-MB-231 treated with ISC-siCREB1. **B** Tumor growth curves of MDA-MB-231 mice in each treatment group. **C** Representative images of SWE, grayscale ultrasound images, Sirius Red staining (scale bar, 100 μm), COL1A1 and α-SMA IF (scale bars, 50 μm) in MDA-MB-231 tumors across treatment groups. **D** Quantitative analysis of COL1A1 IF in MDA-MB-231 tumors across treatment groups. **E** Western blot analysis of CREB1, p-CREB1, TGF-β1, MMP9, N-cadherin and COL1A1 in MDA-MB-231 tumors across treatment groups. Data are presented as mean ± SEM.

**Supplementary Fig. 12 Regulation of adipokine signaling pathway by DAG/PKC axis in the E0771 allograft model.** **A** Glycerolipid levels (diacylglycerols and triacylglycerols) measured by LC–MS/MS in tumor tissues from E0771 mice subjected to control, Diolein, Diolein + Sotrastaurin, or Sotrastaurin treatment. **B, C**KEGG enrichment analysis of lipidomics data from LC–MS/MS, showing significant enrichment of the adipokine signaling pathway in both (**B**) Diolein vs. Diolein + Sotrastaurin and (**C**) Diolein vs. Control comparisons.

**Supplementary Fig. 13 CAFs enhances stromal activation, collagen deposition, and tumor growth in the AT3 model. A-E**Representative images (**A**) and quantitative analysis of α-SMA IHC, Sirius Red staining (**B**) (scale bars, 100 μm), α-SMA(**C**) IF, COL1A1(**D**) IF (scale bars, 50 μm) and LOX levels (**E**) (measured by ELISA) in AT3 alone versus AT3 + NIH-3T3 mixed tumors. **F**Representative images of resected tumors. **G** Tumor weight comparison between AT3 alone and AT3 + NIH-3T3 groups. Data are presented as mean ± SEM.

**Supplementary Fig. 14 Exogenous TGF-β1 further enhances stromal activation and tumor growth in the AT3 and NIH/3T3 co-injection model. A-E**Representative images (**A**) and quantitative analysis of α-SMA IHC, Sirius Red staining (**B**) (scale bars, 100 μm), α-SMA (**C**) IF, COL1A1 (**D**) IF (scale bars, 50 μm) and LOX levels (**E**) (measured by ELISA) comparing AT3 + NIH-3T3 mixed tumors with versus without recombinant TGF-β1 treatment. **F**Representative images of resected tumors. **G**Tumor weight comparison between AT3 + NIH-3T3 mixed groups with and without TGF-β1 treatment. Data are presented as mean ± SEM.

**Supplementary Fig. 15 Correlation of tissue stiffness with collagen deposition. A-C** Correlation between SWE values and area of Sirius Red’s staining (**A**), area of COL1A1 staining (**B**) and LOX content (**C**).

**Supplementary Fig. 16 Comparative analysis of tissue samples from event(recurrent/deceased) TNBC patients (n=10) versus event-free(non-recurrent) TNBC patients (n=10) in the FUSCC validation cohort.** **A, B** IF staining and quantitative analysis of COL1A1(**A**) and α-SMA(**B**). Scale bars, 100μm. Data are presented as mean ± SEM.
