# Supplementary material for: The DAG/PKC/CREB1/TGF-β1 axis drives shear-wave elastography stiffness and malignant progression in triple-negative breast cancer via lipid metabolic reprogramming
Source: Cell Death Dis. 2026 Mar 20;17(1):327. doi: 10.1038/s41419-026-08625-0 (PMC13039978; doi:10.1038/s41419-026-08625-0)
Supplement: Supplementary file 2 — Supplementary Materials and Methods [file 41419_2026_8625_MOESM2_ESM.docx]

**Supplementary Materials and Methods**

**Study population**

This study enrolled TNBC patients who underwent preoperative breast ultrasound examinations between September 2017 and August 2020 from FUSCC. Included patients had: (1) clearly visible lesions on ultrasound images with multiple images of breast tumors; (2) BC diagnosis confirmed by core needle biopsy or surgical pathology. (3) no history of any anticancer treatment prior to surgery. Excluded patients exhibited: (1) invisible or ambiguous lesions on ultrasound; (2) incomplete clinical data. This retrospective study was approved by our institutional ethics committee. Informed consent was waived due to the retrospective nature of the research.

Tissue samples were obtained from the tissue bank of FUSCC. All samples were pathologically diagnosed as TNBC. The use of clinical information and tissues was approved by the Ethics Committee of FUSCC (approval number: 2509-ZZK-153). All patients received chemotherapy and/or immunotherapy before surgical resection of tumor specimens. Treatment response was classified according to the pathological Miller-Payne classification system. HALO imaging analysis was performed using the Visiopharm AI-powered platform, with strict normalization of staining intensity across batches.

**Ultrasound image evaluation**

Most examinations were performed using a SuperSonic Aixplorer US scanner (SuperSonic Imagine S.A., Aix-en-Provence, France) equipped with a 7-15 MHz linear array transducer. Additional breast US images were acquired using a Mindray Resona 5S US scanner (Shenzhen Mindray Bio-Medical Electronics Co., Ltd., Shenzhen, China) with a 5-14 MHz linear array transducer. The image acquisition protocol was as follows: starting from the tumor's largest cross-section, 12 conventional ultrasound images were collected at equal intervals within a 180° clockwise range.

**Lipid metabolomics detection**

To directly exclude the possibility that the effects of CM(D-7-3) were primarily driven by residual DAG carried over from its induction process, we quantified the DAG concentration in the liquid samples of vehicle, CM(D-7), and CM(D-7-3) (n=4 per group).

*Sample preparation:* For lipid extraction, 1 mL of each sample was first lyophilized and then reconstituted in 600 μl methanol–water (1:1, v/v) containing internal standards (cholic acid-2,2,4,4‑d4 and glycocholic acid-(glycine-1-13C)) and 0.1 mM BHT, followed by the addition of 600 μl chloroform. The mixture was sonicated in an ice-water bath (500 W, 3 min, cycle: 6 s on/4 s off) for cell disruption. All liquids were transferred to a 1.5 mL microcentrifuge tube and further extracted by ice-water bath sonication for 10 min. After standing at −20 °C for 30 min, the sample was centrifuged at 13,000 rpm for 10 min (4 °C). A total of 400 μl of the lower chloroform phase was carefully collected, transferred to a high-recovery LC MS vial, and dried under vacuum. The lipid residue was reconstituted in 200 μl isopropanol–methanol (1:1, v/v) with vortexing for 30 s and sonication in an ice-water bath for 3 min, followed by the addition of 20 μl mixed isotope internal standards (details in Appendix). The solution was transferred to a 1.5 mL EP tube, centrifuged again (13,000 rpm, 10 min, 4 °C), and the supernatant was injected into an LC MS vial with an insert for analysis. Quality control (QC) samples were prepared by pooling equal volumes of extracts from all samples.

*LC-MS/MS analysis:* LC-MS/MS analysis was carried out according to the previously established and described method.

*Data processing and statistical analysis*: Data processing and statistical analysis were performed consistently with the methodology detailed in prior sections.

To validate the promotive effect of the entire signaling pathway on lipid metabolism in breast cancer, mammary tumor tissues were collected from four experimental groups of mice: control (CT), Diolein-treated, sotrastaurin-treated, and Diolein plus sotrastaurin co-treated groups (n = 6 per group).

*Sample preparation*: Frozen samples were thawed at room temperature. 30 mg of tissue was weighed and mixed with 400 μl of ice‑cold methanol‑water (1:1, v/v) containing a mixed internal standard (4 μg/mL) and two steel beads. After pre‑cooling at –20 °C for 2 min, the mixture was homogenized using a tissue grinder (45 Hz, 2 min). Subsequently, 400 μl of chloroform was added, followed by vortexing for 30 s, ultrasonic extraction for 10 min in an ice‑water bath, and overnight incubation at –20 °C. The extract was centrifuged (10 min, 12,000 rpm, 4 °C), and approximately 300 μl of the lower chloroform layer was transferred to an LC‑MS vial and dried under a gentle nitrogen stream.The dried lipid residue was reconstituted in 200 μl of isopropanol‑methanol (1:1, v/v) by vortexing for 30 s and ultrasonic extraction for 3 min in an ice‑water bath. The solution was then transferred to a 1.5 mL centrifuge tube, centrifuged again under the same conditions, and 150 μl of the supernatant was collected, passed through a 0.22 μm microfilter, and placed into an LC‑MS vial with an insert for analysis.Quality control (QC) samples were prepared by pooling equal volumes of extract from all individual samples, with each QC volume matching that of the experimental samples. All extraction solvents were pre‑cooled to –20 °C before use.

*LC-MS/MS analysis*: Metabolic profiling was performed on an ACQUITY UPLC I-Class Plus system (Waters Corporation, Milford, USA) interfaced with a Q-Exactive mass spectrometer (Thermo Fisher Scientific, Waltham, MA, USA) fitted with a heated electrospray ionization (ESI) source. The analysis was conducted in both positive and negative ionization modes. Chromatographic separation was accomplished using an ACQUITY UPLC BEH C8 column (1.7 μm, 2.1 mm × 100 mm) kept at a constant temperature of 45°C.The mobile phase consisted of (A) water with 0.1% formic acid (v/v) and (B) acetonitrile with 0.1% formic acid (v/v), with a flow rate of 0.35 mL/min. The gradient program was as follows: 0.01 min, 5% B; 2 min, 5% B; 4 min, 30% B; 8 min, 50% B; 10 min, 80% B; 14 min, 100% B; 15 min, 100% B; 15.1 min, 5% B; 16 min, 5% B. The injection volume was 3 μl, and samples were maintained at 10°C during analysis. Mass spectrometry detection was performed across a mass range of m/z 100–1000. Full MS scans were acquired at a resolution of 70,000, and MS/MS scans (HCD) at 17,500. Collision energies were set to 10, 20, and 40 eV. Key MS parameters were configured as follows: spray voltage: 3800 V (positive mode) and 3200 V (negative mode); sheath gas flow rate: 35 arb; auxiliary gas flow rate: 8 arb; capillary temperature: 320°C; auxiliary gas heater temperature: 350°C; S-lens RF level: 50.

*Data preprocessing and statistical analysis*: To compare the abundance of specific lipids across the four experimental groups (n=6 per group), a stepwise statistical approach was employed. First, the normality of distribution and homogeneity of variance for each lipid were assessed using the Shapiro-Wilk test and Levene's test, respectively.Based on these assumptions, the global differences among all four groups were evaluated using an omnibus test. For data meeting both parametric assumptions, a one-way analysis of variance (ANOVA) was performed. For data violating these assumptions, the non-parametric Kruskal-Wallis H test was used instead.Only when a significant overall difference was indicated by the omnibus test (p < 0.05), post-hoc pairwise comparisons were conducted to identify which specific groups differed. For parametric data, Tukey's Honestly Significant Difference (HSD) test was applied to control for Type I error across all comparisons. For non-parametric data, Dunn's test with a Bonferroni adjustment was used. Lipids with p-value < 0.05 in any post-hoc pairwise comparison were considered statistically significantly different and defined as differentially abundant lipids.

**RNA sequencing**

*RNA extraction and sequencing library preparation：*RNA was extracted from mammary tumor tissues of HFD and CD mice, as well as from Sotrastaurin-treated and control MDA-MB-231 cells. Total RNA was extracted using Trizol reagent (15596026, Invitrogen, CA, USA). The purity and concentration of RNA were measured on a NanoDrop 2000 spectrophotometer (Thermo Scientific, USA), and integrity was verified using an Agilent 2100 Bioanalyzer (Agilent Technologies, Santa Clara, CA, USA). Sequencing libraries were prepared with the VAHTS Universal V10 RNA-seq Library Prep Kit (Premixed Version) in accordance with the manufacturer's guidelines. Transcriptome sequencing and subsequent bioinformatic analyses were performed by OE Biotech Co., Ltd. (Shanghai, China).

**Western blot assay**

*Cellular samples:* MDA-MB-231 and E0771 cells were seeded into 6-well plates. When cells reached approximately 60% confluence, cells were treated with Diolein (3μg/ml) and Sotrastaurin (15μmol/L). Cellular proteins were extracted 48 hours later.

MDA-MB-231 cells were seeded into 6-well plates. When cells reached approximately 50% confluence, separate transfection complexes were prepared by respectively mixing the NC siRNA or the CREB1-targeting siRNA (A01001, GenePharma, Suzhou, China) with the transfection reagent in the provided buffer. The mixtures were incubated for 10 minutes at room temperature to form complexes, and then added to the culture medium of their respective groups. Cellular proteins were extracted after 48 hours of transfection.

MDA-MB-231 and E0771 cells were seeded into 6-well plates. When cells reached approximately 60% confluence, cells were treated with PMA (100nmol/L) and 666-15 (500nmol/L). Cellular proteins were extracted 48 hours later.

MDA-MB-231 and E0771 cells were seeded into 6-well plates. When cells reached approximately 60% confluence, cells were treated with SRI-011381 (30μmol/L) and 666-15 (500nmol/L). Cellular proteins were extracted 48 hours later.

NIH-3T3 cells were seeded into 6-well plates. When cells reached approximately 60% confluence, cells were treated with E0771 conditioned medium (medium collected 72 hours after treating tumor cells with Diolein (3μg/ml)) and P144 (10μmol/L). Cellular proteins were extracted 48 hours later.

E0771 cells were seeded into 6-well plates. When cells reached approximately 60% confluence, cells were treated with NIH-3T3 CM (medium from NIH-3T3 cells cultured for 72 hours), NIH-3T3 CM treated with E0771 CM, and D-7-3CM. Cellular proteins were extracted 48 hours later.

*Tissue samples:* Tumor tissues from HFD and CD mice were mixed with NP-40 and two steel balls. The homogenization was performed at a frequency of 25-30 Hz, in cycles of 30 seconds on / 30 seconds off, for a total of 3-5 cycles, while the sample chamber was kept at 4°C. After grinding with a tissue homogenizer (12000rpm，15min，4℃) , the supernatant was collected by centrifugation for BCA quantification.

*Protein extraction and quantification*：Total protein was extracted from adherent cells using direct lysis. Briefly, after reaching appropriate confluence, the culture medium was completely removed. Cells were then gently rinsed twice with phosphate-buffered saline (PBS) to remove residual medium. An appropriate volume of RIPA lysis buffer (P0013B, Beyotime Institute of Biotechnology, Shanghai, China) supplemented with 50× protease inhibitor cocktail and 50× phosphatase inhibitor cocktail (P1045, Beyotime Institute of Biotechnology, Shanghai, China) was directly added to the culture dish. Cells were lysed on ice for 15 minutes with occasional gentle agitation. Subsequently, the adherent lysate was thoroughly scraped using a cell scraper and transferred to a pre-cooled microcentrifuge tube. The lysate was clarified by centrifugation at 12,000 × g for 15 minutes at 4°C to remove insoluble debris. The supernatant (total protein extract) was collected, and the protein concentration was determined using a BCA Protein Assay Kit (P0010, Beyotime Institute of Biotechnology, Shanghai, China) according to the manufacturer‘s instructions. For Western blot analysis, protein extracts were mixed with 5× SDS-PAGE loading buffer (P0015, Beyotime Institute of Biotechnology, Shanghai, China) at a ratio of 4:1 (protein extract : loading buffer), followed by denaturation at 95°C for 5-10 minutes. Denatured protein samples were aliquoted and stored at -80°C or used immediately for electrophoresis. Subsequently, protein samples were separated by SDS-PAGE and transferred to PVDF membranes. Membranes were incubated with specific primary antibodies against target proteins. All primary antibodies used in the [western blot analysis](https://www.sciencedirect.com/topics/medicine-and-dentistry/western-blot) are listed in Table S1.

**CCK-8 assay**

The CCK-8 assay was used to evaluate the experimental concentration of Sotrastaurin, using CCK-8 reagent (C0037, Beyotime Institute of Biotechnology, Shanghai, China). Initially, MDA-MB-231 and E0771 cells were seeded in 96-well plates (2000 cells/well) and incubated for 24 hours. Subsequently, cells were treated with Sotrastaurin (15μmol/L). After 48 hours, CCK-8 reagent was added to the plates (mixed with DMEM 1:9), followed by further incubation at 37°C for 1 hour. Cell proliferation was measured by recording the absorbance at 450 nm, and growth curves were visualized.

To directly exclude the possibility that the effects of CM(D-7-3) were primarily driven by residual DAG carried over from its induction process, we assessed the effect of diolein at a concentration gradient on tumor cell proliferation using the CCK-8 assay.

MDA-MB-231 cells were seeded in 96-well plates (2000 cells/well). After 24 hours of incubation, cells were treated with treated with diolein (0μg/ml, 0.5μg/ml, 1μg/ml, 3 μg/ml) and CM(D-7-3). Cell proliferation was monitored for 7 consecutive days. Each day, CCK-8 reagent (mixed with DMEM at a 1:9 ratio) was added to the plates, followed by incubation at 37°C for 1 hour. The absorbance at 450 nm was recorded daily, and growth curves were visualized to compare proliferation dynamics.

**Colony formation assay**

MDA-MB-231 and E0771 cells were seeded in 12-well plates at a density of 700 cells per well. Cells were treated with Diolein (3 μg/ml) or Sotrastaurin (15 μmol/L). The culture medium was replaced every three days. After 10 days, cells were washed twice with PBS and fixed with formaldehyde for 30 minutes. Each well was then incubated with 500 μl of crystal violet solution for 30 minutes. Following two additional PBS washes, the plates were air-dried and photographed. Results were analyzed using ImageJ software. Groups treated with SRI-011381 and 666-15 were processed using the same protocol. Groups treated with SRI-011381 and 666-15, groups transfected with CREB1-targeting siRNA, and groups treated with diolein at a concentration gradient or CM(D-7-3) were all processed using the same protocol.

**Wound healing assay**

MDA-MB-231 and E0771 cells were seeded in 6-well plates at 2×10^5 cells per well. When cells reached 90%-95% confluency, wounds were created using a 200-μl pipette tip. Cells were then cultured in medium containing 1% FBS and treated with Diolein (3 μg/ml) or Sotrastaurin (15 μmol/L). Photographs were taken at 0 and 48 hours, and analysis was performed using ImageJ. Groups treated with SRI-011381 and 666-15, groups transfected with CREB1-targeting siRNA, and groups treated with diolein at a concentration gradient or CM(D-7-3) all followed the same experimental procedure.

**Transwell assay**

For invasion assays, 60 μl of a Matrigel/PBS mixture (8:1 ratio, 356234, Corning, NY, USA) was added to the upper chamber and allowed to solidify at 37°C for 3 hours. The chambers were then hydrated with 200 μl of serum-free medium for 1 hour. Migration assays were performed without Matrigel coating or hydration. MDA-MB-231 and E0771 cells (pretreated with Diolein (3 μg/ml) or Sotrastaurin (15 μmol/L) for 48 hours) were seeded in 24-well plates at 1×10^5 cells per well. After 48 hours, the upper chambers were removed and cells were fixed with formaldehyde for 30 minutes. Cells were stained with crystal violet for 30 minutes, washed with PBS, air-dried, and photographed. Groups treated with SRI-011381, 666-15, groups transfected with CREB1-targeting siRNA, and groups treated with NIH-3T3 CM were all processed using the same protocol.

**Quantification of total TAG, cholesterol, and neutral lipids**

TAG levels were quantified using a triglyceride quantification kit (A110-1-1, Nanjing Jiancheng Bioengineering Institute, Nanjing, China). Cholesterol levels were measured using a cholesterol assay kit (A111-1-1, Nanjing Jiancheng Bioengineering Institute, Nanjing, China). For neutral lipid quantification, 5×10^3 TNBC cells were seeded in 24-well plates. After seeding, cells were fixed with 4% paraformaldehyde for 30 minutes. Cells were then incubated with 0.2 μg/ml BODIPY 493/503 (GC42959, GLPBIO, Montclair, CA, USA) in the dark at 37°C for 30 minutes. Cell images were captured using microscopy, and fluorescence intensity of neutral lipids was analyzed using ImageJ software.

**Bioinformatics analysis**

Bioinformatics analysis was performed using the external validation dataset GSE137467 from the GEO database, which included 3 normal samples and 3 PKCi-treated samples. Gene expression differences between the two groups were analyzed using R software with the GEOquery, limma, ggplot2, and ggpubr packages, focusing specifically on comparing CREB1 mRNA expression levels and generating group comparison plots. Additionally, a CREB1 target gene set was obtained from the DoRothEA database and used to perform Gene Set Enrichment Analysis (GSEA) to further investigate CREB1-related signaling pathways.

**Immunofluorescence staining (IF)**

*Cell samples:* MDA-MB-231 and E0771 cells were treated with NIH-3T3 CM with or without P144 (10 μmol/L) and recombinant TGF-β1 cytokine (10 ng/ml) for 48 hours. Subsequently, the cells were fixed with 4% paraformaldehyde at room temperature for 10-15 minutes, followed by three washes with PBS (10 minutes each).

*Tissue samples:* Tissues were fixed in tissue fixative for over 48 hours. All subsequent procedures were performed by professional personnel who were blinded to the group allocation of the specimens. IF was performed on formalin-fixed paraffin-embedded (FFPE) sections from mouse models. Primary antibodies for IF staining included COL1A1 antibody (GB114197, Servicebio, Wuhan, China), α-SMA antibody (GB111364, Servicebio, Wuhan, China), Ki67 antibody (27309-1-AP, Proteintech, Wuhan, China), and Actin-Tracker Red-Rhodamine (C2207S, Beyotime Institute of Biotechnology, Shanghai, China). All procedures were performed by skilled laboratory personnel who were blinded to tissue sample groups throughout the process, focusing only on technical execution without participating in subsequent data analysis.

**Immunohistochemistry (IHC)**

IHC was performed on FFPE sections from mouse models. Primary antibodies for IHC included PKC antibody (12919-1-AP, Proteintech, Wuhan, China) and α-SMA antibody (GB111364, Servicebio, Wuhan, China). All procedures were performed by skilled laboratory personnel who were blinded to tissue sample groups throughout the process, focusing only on technical execution without participating in subsequent data analysis.

**Tissue collagen staining and quantification**

For Masson staining (G1340, Solarbio, Beijing, China), paraffin-embedded sections of breast cancer tissue samples were stained according to the manufacturer's instructions to examine collagen content and arrangement. After staining, fibrous collagen appeared green or blue, while muscle fibers appeared red.

For Sirius Red staining (G1472, Solarbio, Beijing, China), BC tissue paraffin sections were stained, with Sirius Red specifically binding to the helical structure of fibrous collagen and Fast Green binding to non-collagenous proteins in the tissue. Entire stained sections were scanned using a slide scanner (Leica) at 20× magnification. Three random fields were selected from each section for subsequent statistical analysis, and collagen content in breast cancer tissue was quantified using ImageJ software.

**ELISA assay**

The concentration of TGF-β1 in cell culture supernatants and serum samples was determined using a TGF-β1 ELISA kit(U96-1615E, YoYBiotech, Shanghai, China) according to the manufacturer's protocol. Cell culture supernatants were collected and centrifuged at 1000 × g for 10 minutes to remove cellular debris. Serum samples were obtained by allowing whole blood to clot at room temperature for 30 minutes, followed by centrifugation at 2000 × g for 15 minutes. All samples were stored at -80°C until analysis. For the assay, 50 µL of standard or prepared sample was added to each well of the pre-coated plate; the blank wells received 50 µL of the sample diluent provided. The plate was gently mixed and then incubated at 37°C for 50 minutes. After incubation, the plate was washed three times with 1× wash buffer (300 µL per well per wash) with 1 minute soak period between washes, followed by thorough blotting on absorbent paper. Subsequently, 100 µL of the prepared 1× biotinylated detection antibody working solution was added to all sample and standard wells, while 100 µL of the antibody diluent was added to the blank wells. The plate was incubated at 37°C for 50 minutes and then washed three times as described above. Next, 100 µL of the streptavidin-biotin-peroxidase complex (SABC) working solution was added to every well. The plate was incubated at 37°C for 30 minutes, followed by another three washes. For color development, 100 µL of the prepared TMB substrate solution was added to each well. The plate was incubated in the dark at 37°C for 10-20 minutes (the incubation time was adjusted based on the observed color intensity). The reaction was stopped by adding 50 µL of stop solution per well. The optical density (OD) at 450 nm was measured within 30 minutes using a microplate reader. The concentration of TGF-β1 in the samples was calculated by interpolating from the standard curve generated with the provided standards.

LOX protein levels in tumor tissues were quantified using LOX ELISA kit(H447-1, Nanjing Jiancheng Bioengineering Institute, Nanjing, China) according to the manufacturer's instructions. Briefly, frozen tissue samples were homogenized on ice in phosphate-buffered saline (PBS, pH 7.4) at a weight-to-volume ratio of 1:9 (e.g., 1 g tissue per 9 mL PBS) using a tissue homogenizer (12000rpm，15min，4℃). The homogenates were then centrifuged at 2000 × g for 20 minutes at 4°C, and the resulting supernatants were collected for analysis. For the assay, 50 µL of each prepared supernatant or standard was added to the antibody-precoated wells, followed by 50 µL of the biotinylated detection antibody working solution. The plate was incubated at 37°C for 30 minutes. After incubation, the plate was washed five times with the provided wash buffer. Subsequently, 50 µL of the avidin-horseradish peroxidase (HRP) conjugate working solution was added to each well, and the plate was incubated at 37°C for another 30 minutes, followed by another five washes. Next, 50 µL each of chromogen substrate solution A and B were added to the wells. The plate was incubated in the dark at 37°C for 10 minutes for color development. The reaction was then stopped by adding 50 µL of stop solution to each well. The optical density (OD) at 450 nm was measured within 10 minutes using a microplate reader. The LOX concentration in each sample was calculated based on the standard curve using the ELISAcalc software.

**Bioluminescence imaging**

4T1-Luc cells (2×10^5 per mouse) were injected into the tail vein of BALB/c female mice. Ten days later, after intraperitoneal injection of 2.5 mg luciferin (PerkinElmer), mice were imaged using an IVIS Lumina system and Living Image software 4.0 (PerkinElmer). Total signal in each defined region of interest was calculated as photons/s/cm² (total flux/area). Mice were anesthetized with isoflurane inhalation, and blood samples were collected from the abdominal aorta.

**Animal experiments**

Female BALB/c nude mice and BALB/c and C57BL/6J female mice (4-6 weeks old) were purchased from Gempharmatech (Nanjing, China) and maintained under SPF conditions. All animal experiments were approved by the Ethics Committee of Fudan University Cancer Hospital. Mice were housed under controlled circadian conditions (25°C, 55% humidity) with free access to food and water. Tumor studies were conducted only in female mice. Mice were randomly assigned to their respective experimental groups using a computer-generated randomization schedule. To minimize bias, the investigators responsible for outcome assessment (tumor measurement and SWE imaging) and data analysis were blinded to the group allocations.  Tumor volume was calculated using the formula (L × W²)/2, where L represents length and W represents width. To comply with ethical guidelines and ensure data quality, animals were humanely euthanized before tumors reached the maximum allowable size (2000 mm³). The group size (n=10) was chosen based on previous studies [15-17] and our own preliminary experiments to ensure sufficient statistical power.

To evaluate the effects of hyperactivated lipid metabolism on tumor growth in vivo, twenty 4-6 weeks old female C57BL/6J mice were divided into 2 groups of 10 mice each. Mice received either HFD (60% kcal fat; D12492, Research Diets, NJ, USA) or CD (10% kcal fat; D12450, Research Diets, NJ, USA). After 40 days, 1×10^6 E0771 cells in 100 μl PBS were implanted into the third mammary fat pad. The cell suspension was prepared in 100 μl PBS mixed with an equal volume of Matrigel (356234, Corning, NY, USA). Tumor volume was measured using calipers and calculated using the formula (L × W²)/2. SWE imaging was performed on day 21, with additional SWE imaging on day 28. Mice were euthanized by CO₂ inhalation, tumors were extracted and weighed, and tumor samples were photographed. The 4T1 cell group was treated using the same method, using BALB/c female mice, with SWE imaging performed only on day 28.

To evaluate the effects of DAG/PKC/CREB1/TGF-β1 on tumor growth in vivo, seventy 4-6 weeks old female C57BL/6J mice were divided into 7 groups of 10 mice each. Then, 1×10^6 E0771 cells in 100 μl PBS were implanted into the third mammary fat pad. The cell suspension was prepared in 100 μl PBS mixed with an equal volume of Matrigel (356234, Corning, NY, USA). Mice subsequently received intraperitoneal injections every three days of Diolein (30 mg/kg), Sotrastaurin (20 mg/kg), 666-15 (20 mg/kg), or SRI-011381 (30 mg/kg). Tumor volume was measured using calipers and calculated using the formula (L × W²)/2. On day 25, SWE imaging was performed, mice were euthanized by CO₂ inhalation, tumors were extracted and weighed, and tumor samples were photographed. The AT3 cell group was treated using the same method.

To further validate the axis and assess the therapeutic potential of targeting key downstream nodes, seventy 4-6 weeks old female BALB/c nude mice were divided into 7 groups of 10 mice each. Mice were randomly assigned to their respective experimental groups using a computer-generated randomization schedule. To minimize bias, the investigators responsible for outcome assessment (tumor measurement and SWE imaging) and data analysis were blinded to the group allocations. Then, 1.5×10^6 MDA-MB-231 cells in 100 μl PBS were implanted into the third mammary fat pad. The cell suspension was prepared in 100 μl PBS mixed with an equal volume of Matrigel (356234, Corning, NY, USA). Mice subsequently received intraperitoneal injections every three days of Diolein (30 mg/kg), Sotrastaurin (20 mg/kg), P144 diammonium (20 mg/kg), or BAPN (20 mg/kg). The siRNA/polyplex mixture of ISC-siCREB1 was administered to mice via intraperitoneal injection at a dose of 5 µg siRNA per gram of mouse body weight. Injections were performed once per week. Tumor volume was measured using calipers and calculated using the formula (L × W²)/2. On day 22, SWE imaging was performed, mice were euthanized by CO₂ inhalation, tumors were extracted and weighed, and tumor samples were photographed.

To evaluate the effects of tumor cell-CAFs interactions on tumor growth in vivo, twenty 4-6 weeks old female BALB/c nude mice were divided into 2 groups of 10 mice each. The control group received implantation of 1×10^6 AT3 cells in 100 μl PBS into the third mammary fat pad, while the experimental group received additional implantation of 5×10^5 NIH-3T3 cells. The cell suspension was prepared in 100 μl PBS mixed with an equal volume of Matrigel (356234, Corning, NY, USA). Tumor volume was measured using calipers and calculated using the formula (L × W²)/2. On day 25, SWE imaging was performed, mice were euthanized by CO₂ inhalation, tumors were extracted and weighed, and tumor samples were photographed. Mice treated with recombinant TGF-β1 cytokine (2 mg/kg) received intraperitoneal injections every three days, while other methods followed the above protocol.
