# Supplementary material for: The DAG/PKC/CREB1/TGF-β1 axis drives shear-wave elastography stiffness and malignant progression in triple-negative breast cancer via lipid metabolic reprogramming
Source: Cell Death Dis. 2026 Mar 20;17(1):327. doi: 10.1038/s41419-026-08625-0 (PMC13039978; doi:10.1038/s41419-026-08625-0)

Figure 1K

FASN (66591-1-Ig, Proteintech)

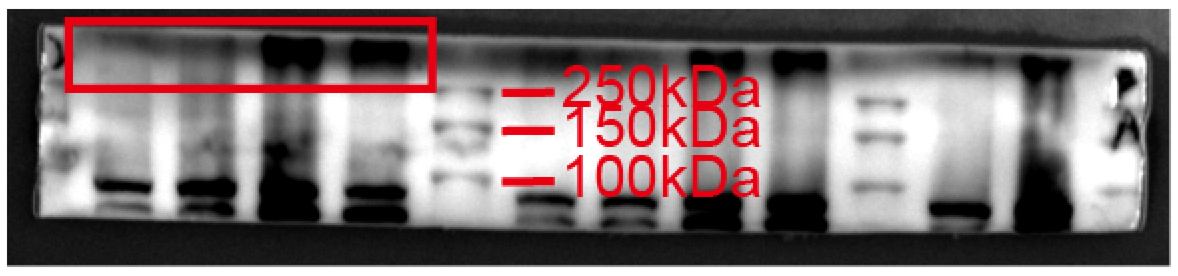

SREBP (A25305, abclonal)

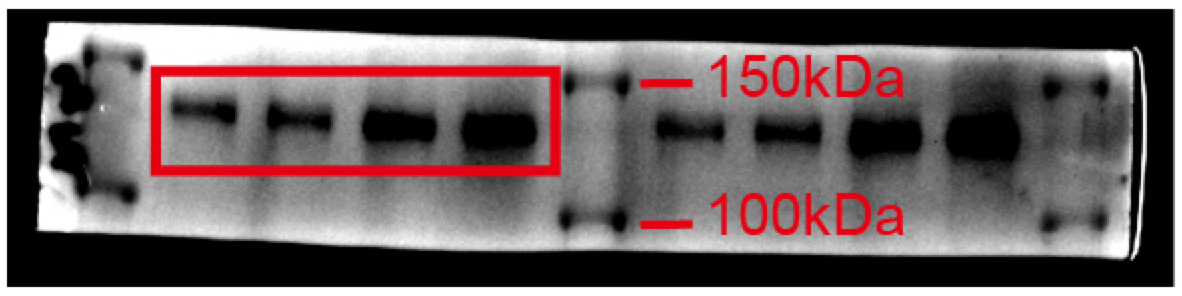

PPARγ (66936-1-Ig, Proteintech)

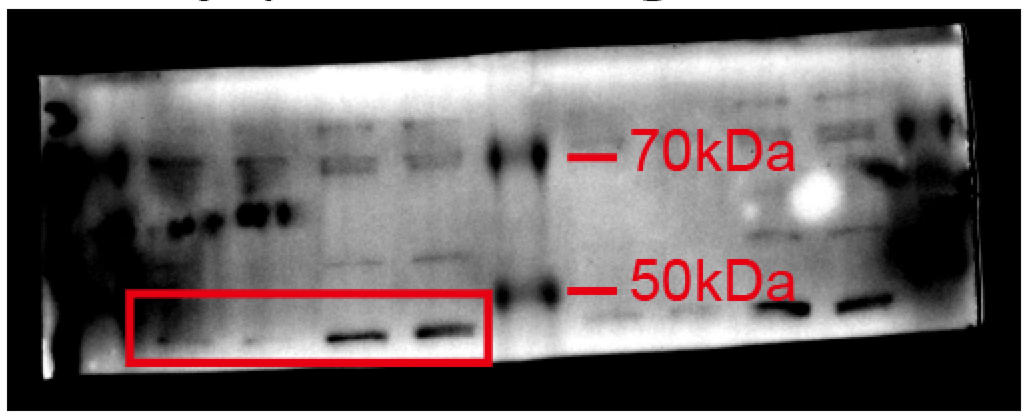

GAPDH (60004-1-Ig, Proteintech)

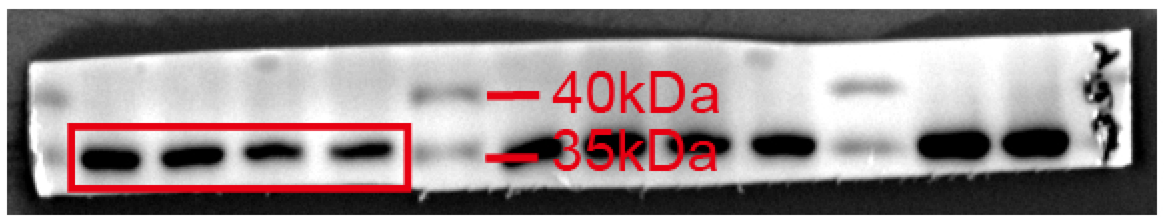

Figure 3B

PKC (A24003, abclonal)

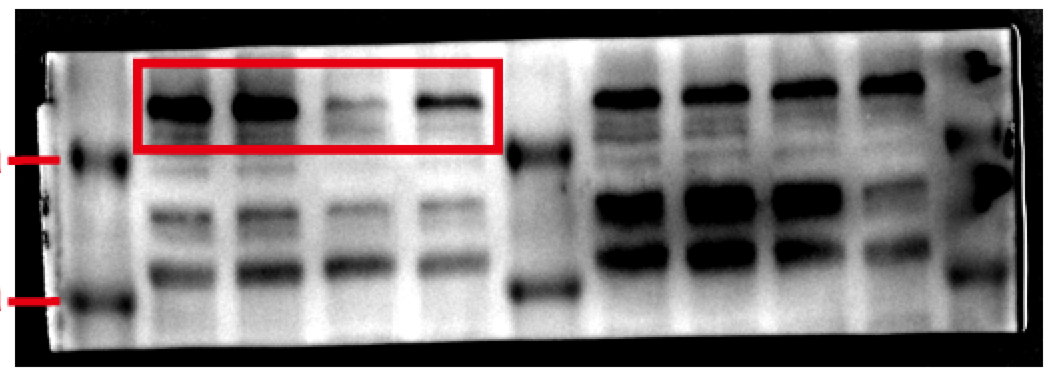

p-PKC (AP1414, abclonal)

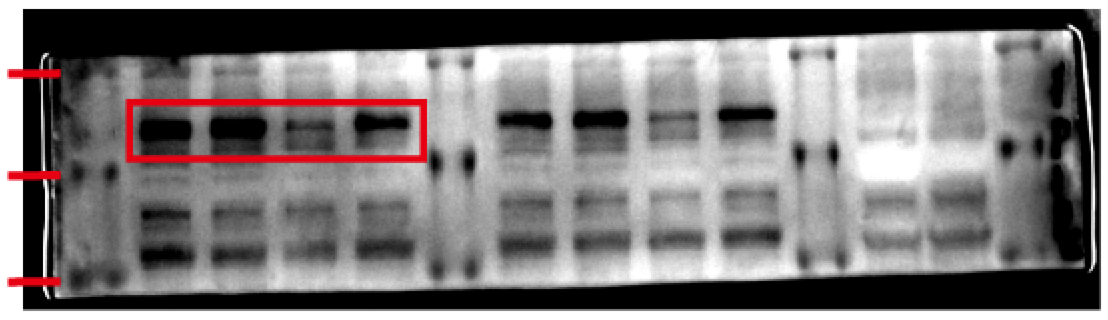

MMP-9 (10375-2-AP, Proteintech)

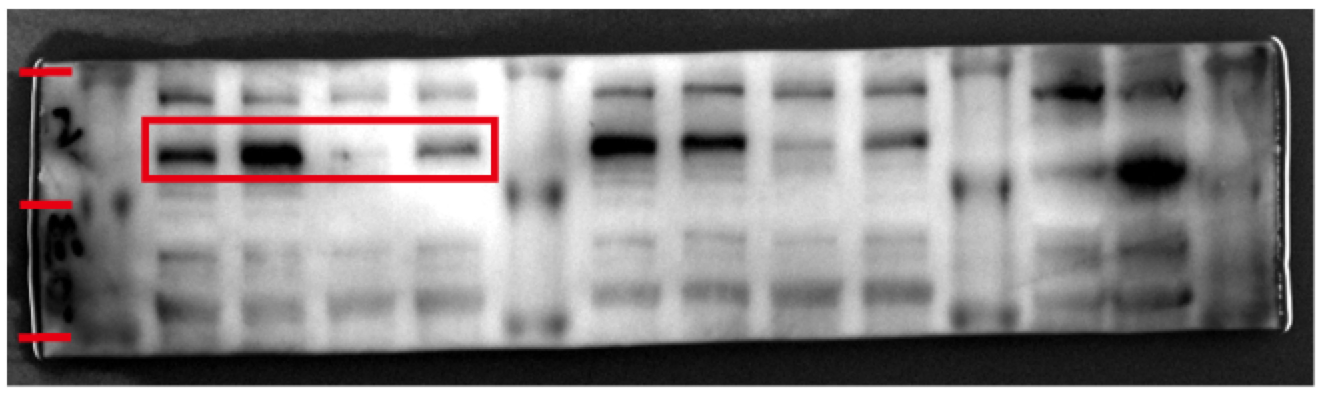

E-cadherin (GB11082, Servicebio)

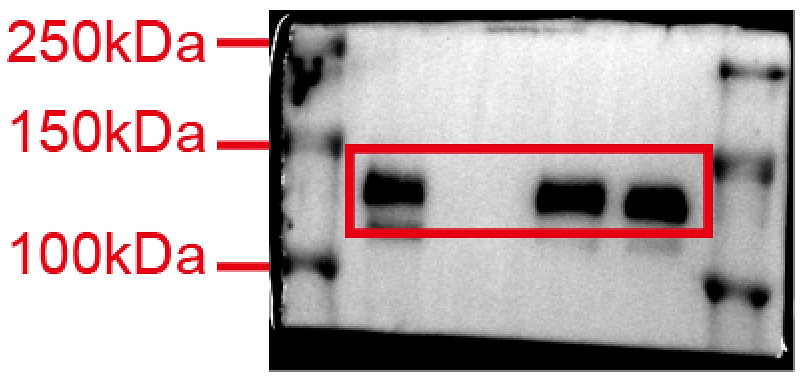

N-cadherin (GB12135, Servicebio)

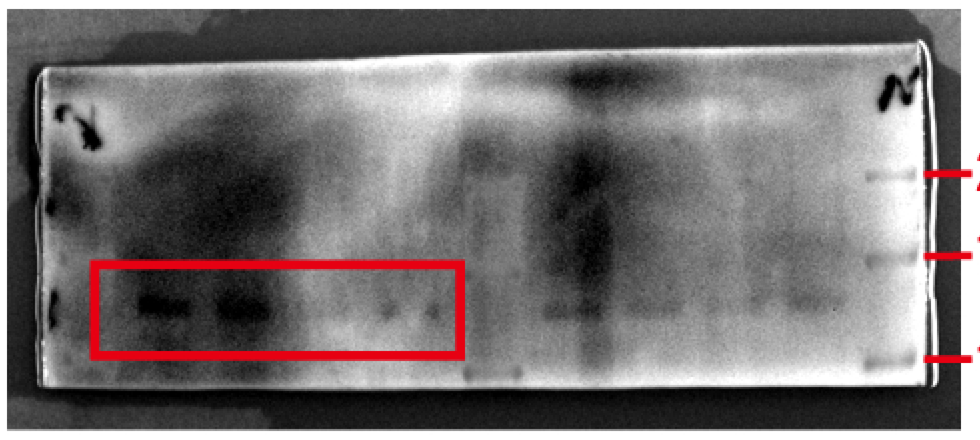

GAPDH (60004-1-Ig, Proteintech)

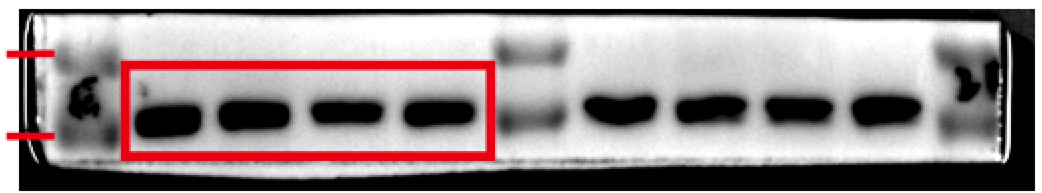

Figure 3C

PKC (A24003, abclonal)

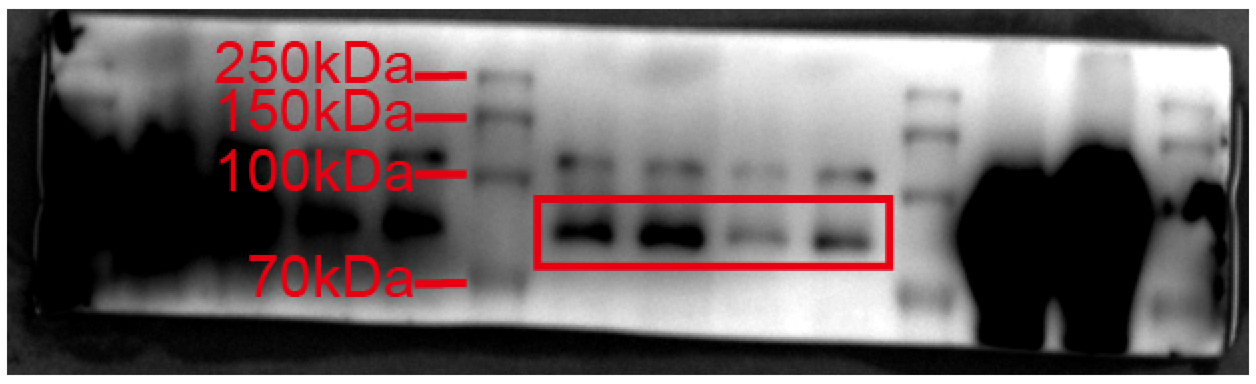

p-PKC (AP1414, abclonal)

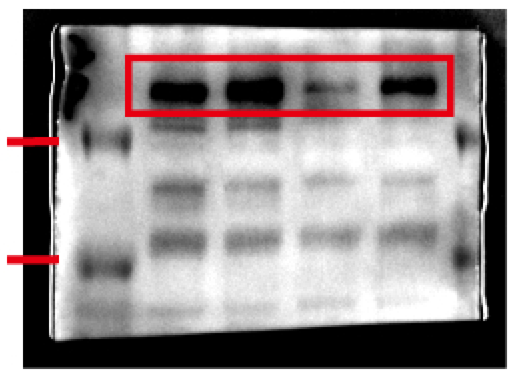

MMP-9 (10375-2-AP, Proteintech)

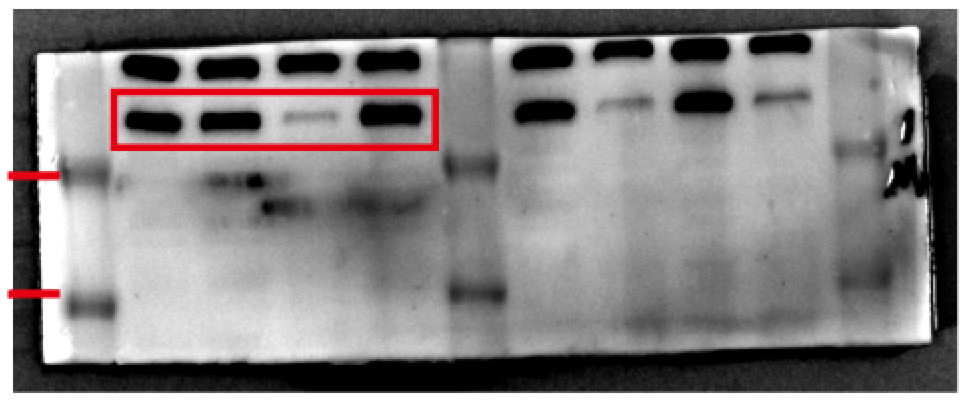

E-cadherin (GB11082, Servicebio)

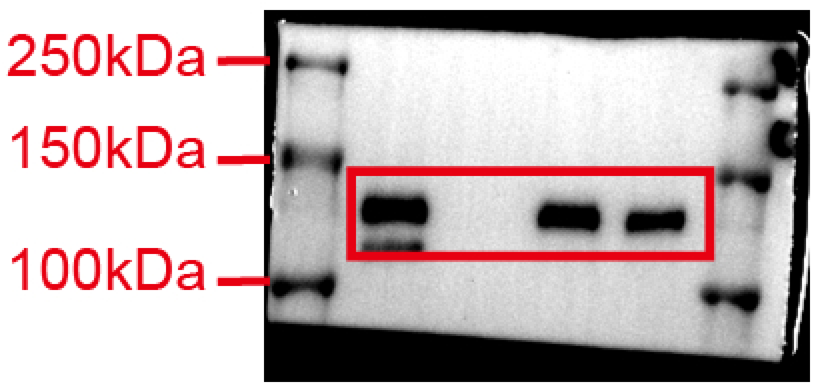

N-cadherin (GB12135, Servicebio)

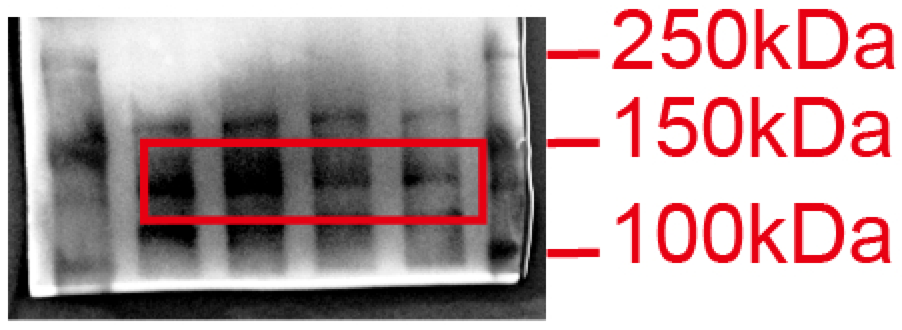

GAPDH (60004-1-Ig, Proteintech)

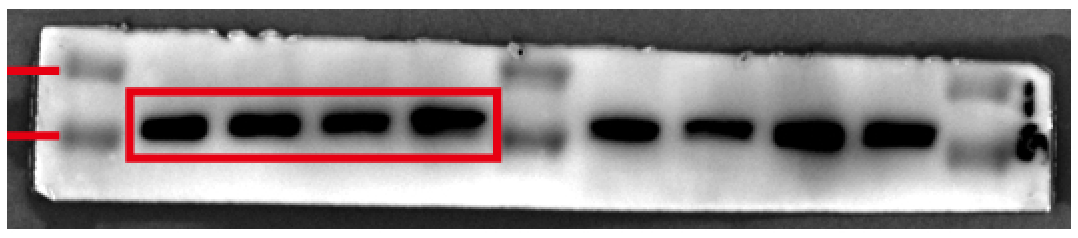

Figure 4C

CREB1 (12208-1-AP, Proteintech)

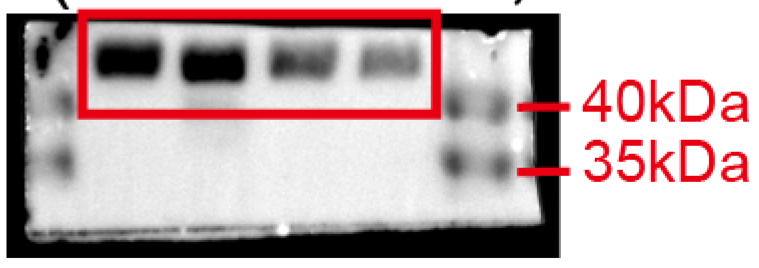

p-CREB1 (28792-1-AP, Proteintech)

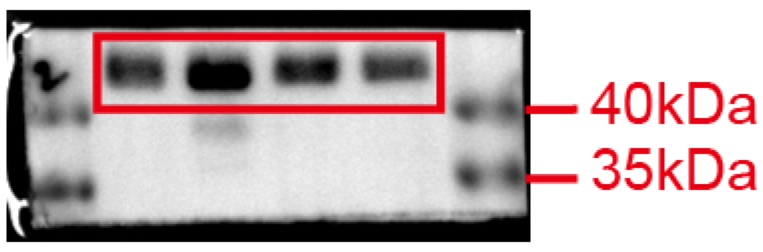

Tubulin (11224-1-AP, Proteintech)

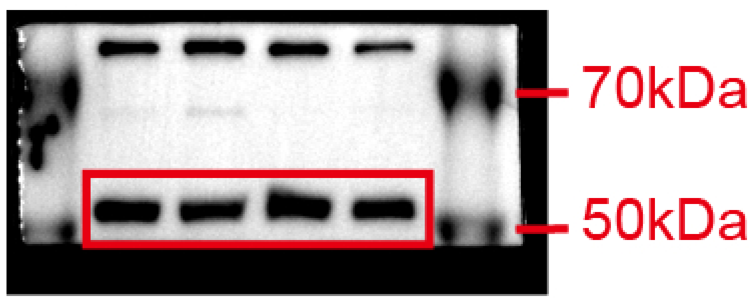

Figure 4D

CREB1 (12208-1-AP, Proteintech)

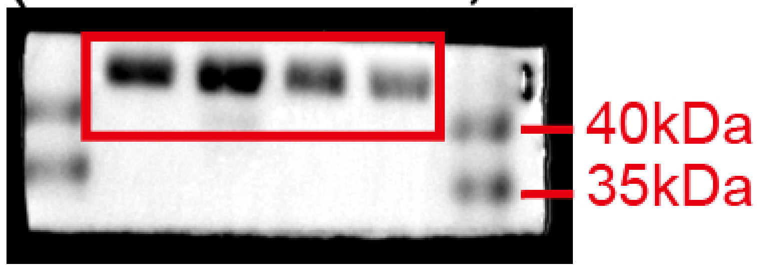

p-CREB1 (28792-1-AP, Proteintech)

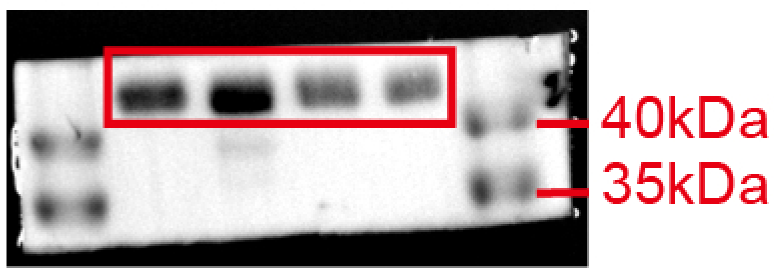

Tubulin (11224-1-AP, Proteintech)

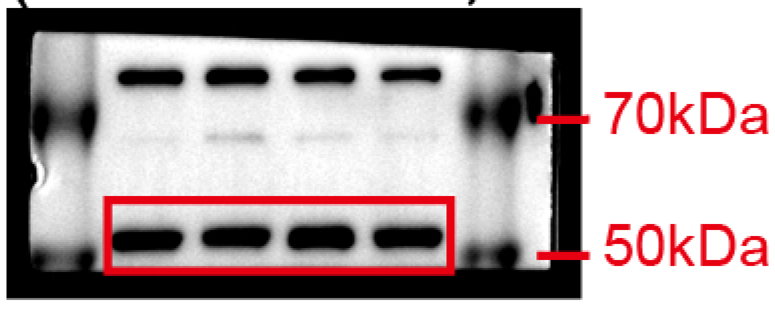

Figure 4G

CREB1 (12208-1-AP, Proteintech)

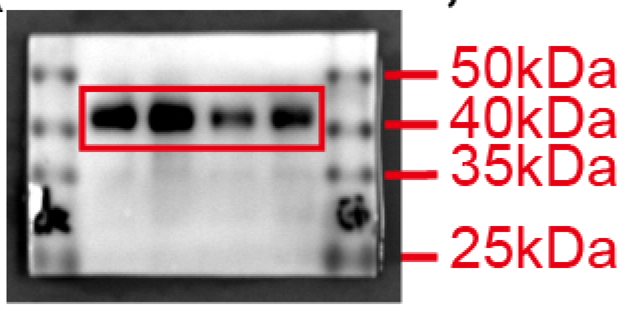

p-CREB1 (28792-1-AP, Proteintech)

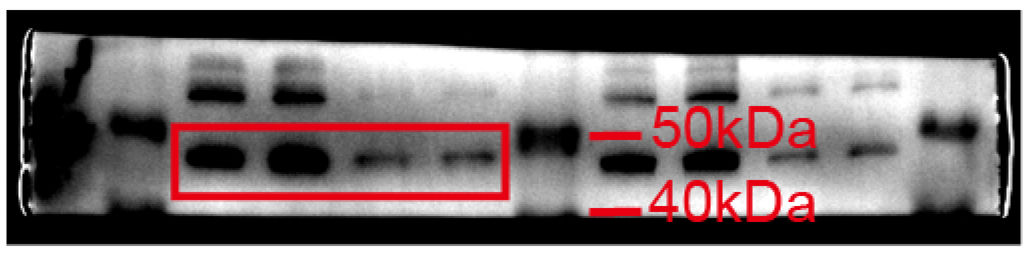

TGF-β1 (ab215715, Abcam)

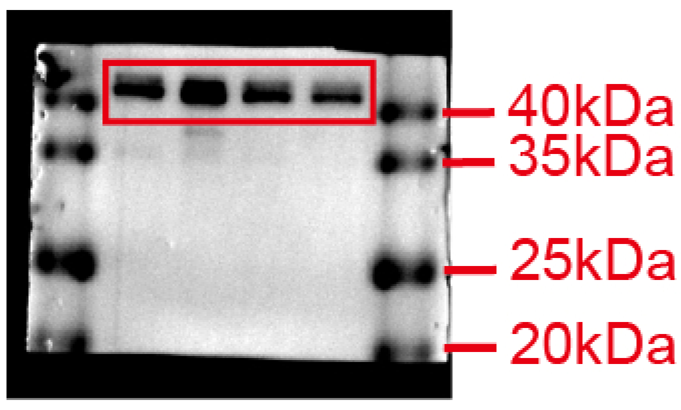

Tubulin (11224-1-AP, Proteintech)

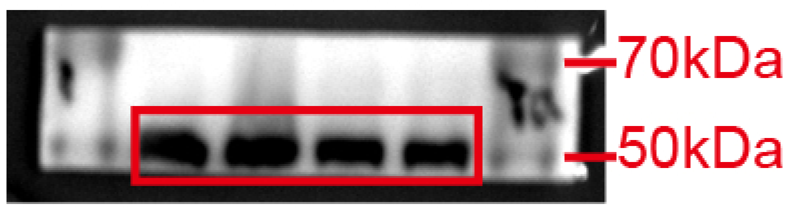

Figure 4H

CREB1 (12208-1-AP, Proteintech)

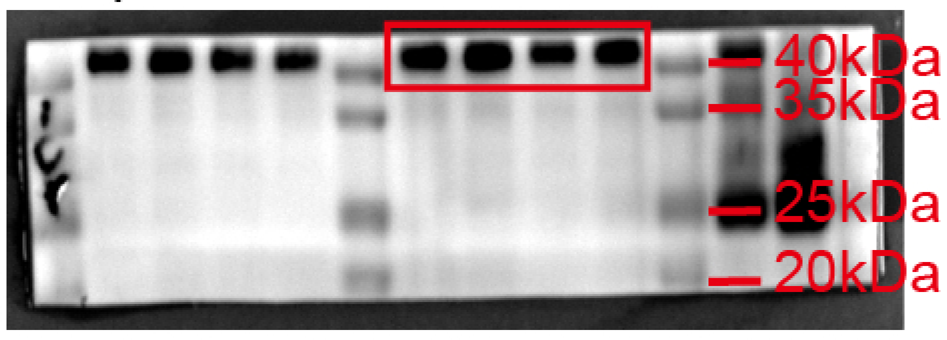

p-CREB1 (28792-1-AP, Proteintech)

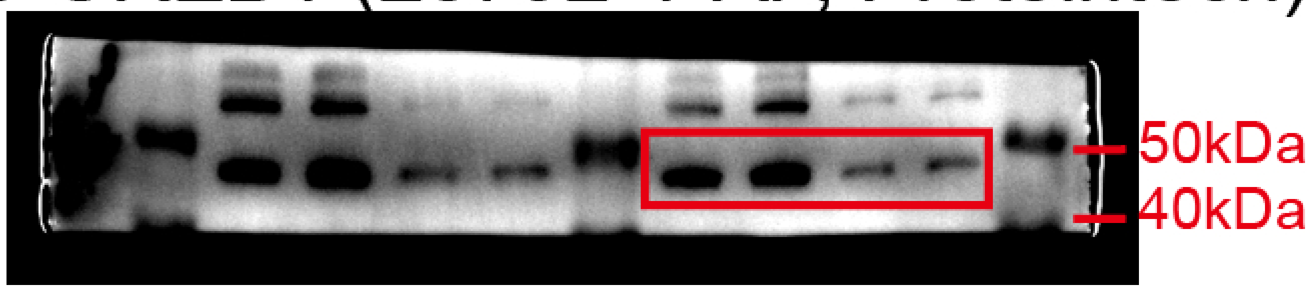

TGF-β1 (ab215715, Abcam)

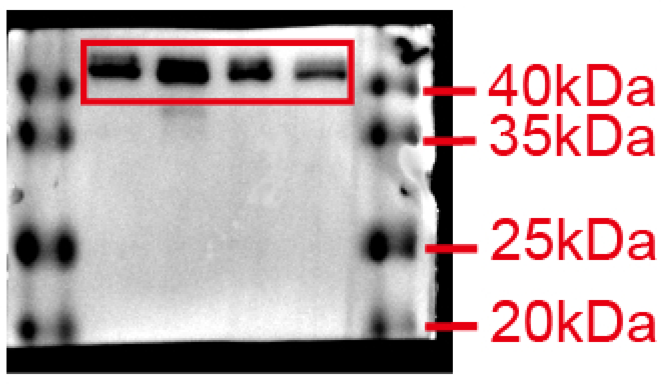

Tubulin (11224-1-AP, Proteintech)

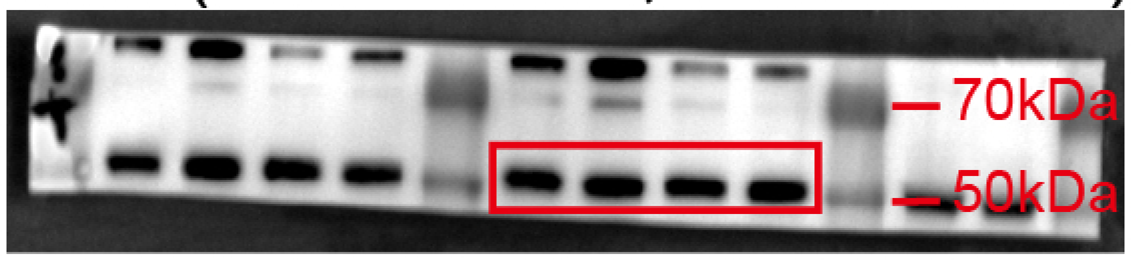

Figure 4I

MMP-9 (10375-2-AP, Proteintech)

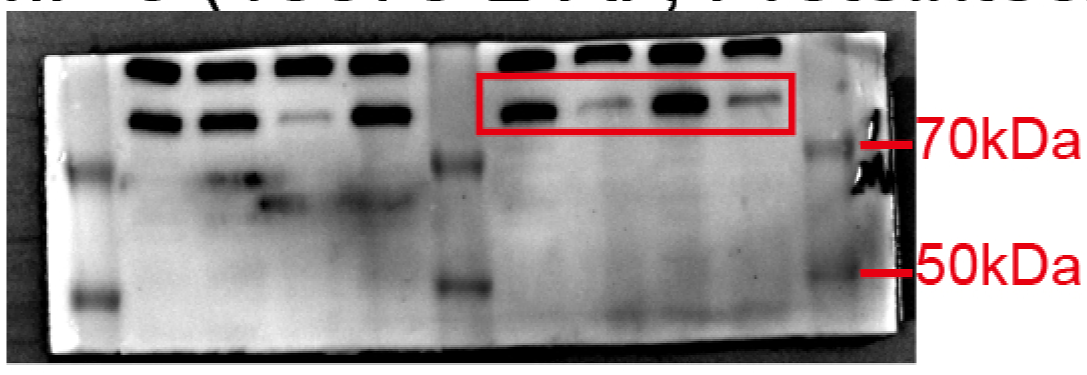

E-cadherin (GB11082, Servicebio)

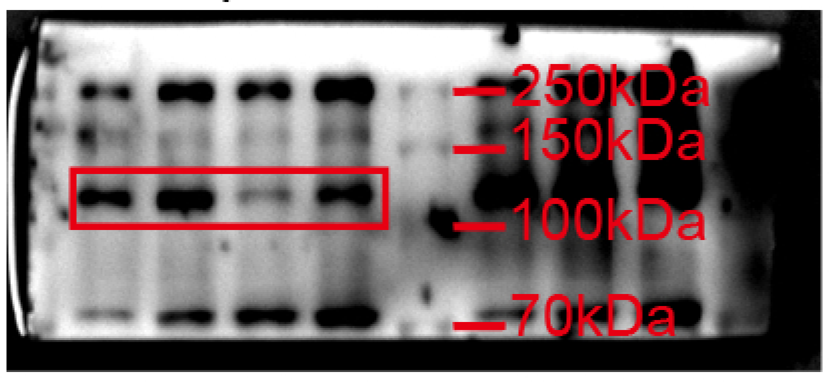

N-cadherin (GB12135, Servicebio)

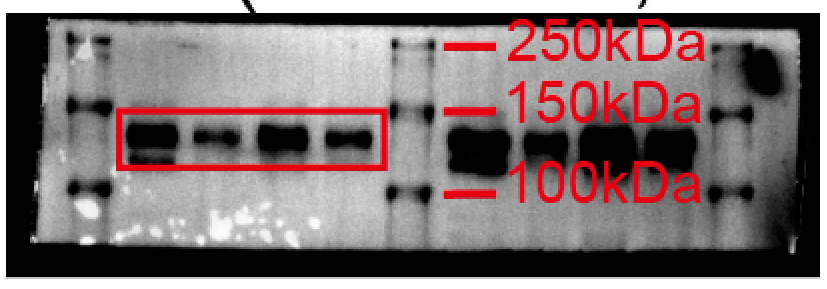

GAPDH (60004-1-Ig, Proteintech)

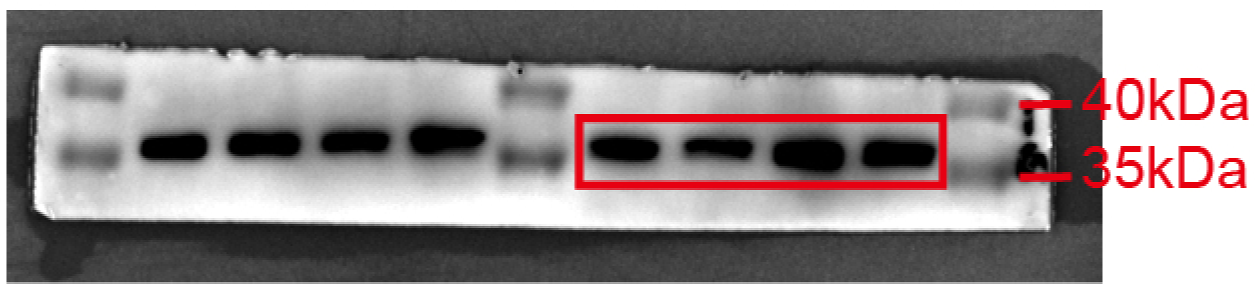

Figure 4F

CREB1 (12208-1-AP, Proteintech)

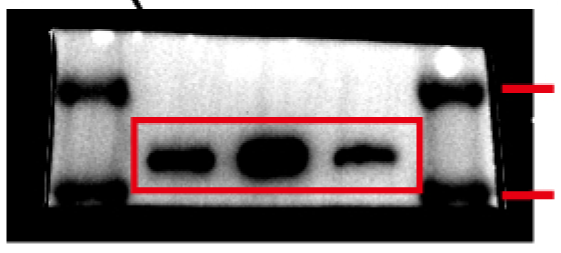

p-CREB1 (28792-1-AP, Proteintech)

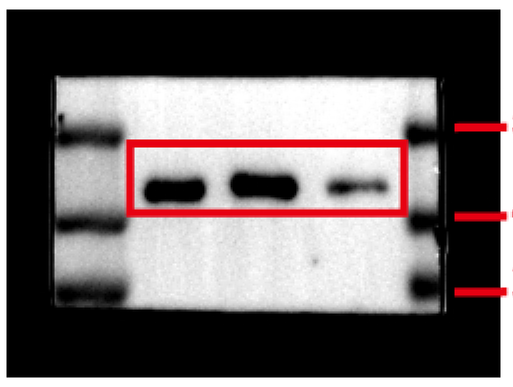

TGF-β1 (ab215715, Abcam)

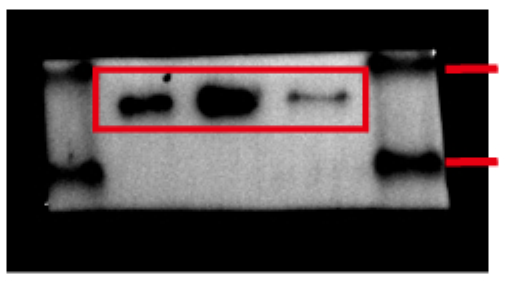

MMP-9 (10375-2-AP, Proteintech)

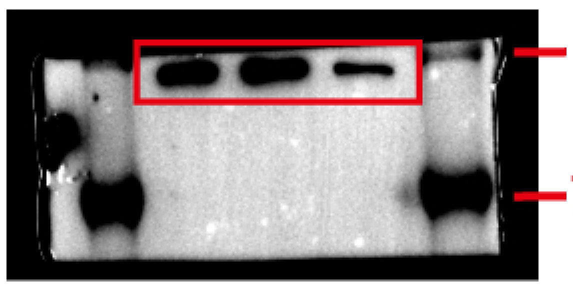

N-cadherin (GB12135, Servicebio)

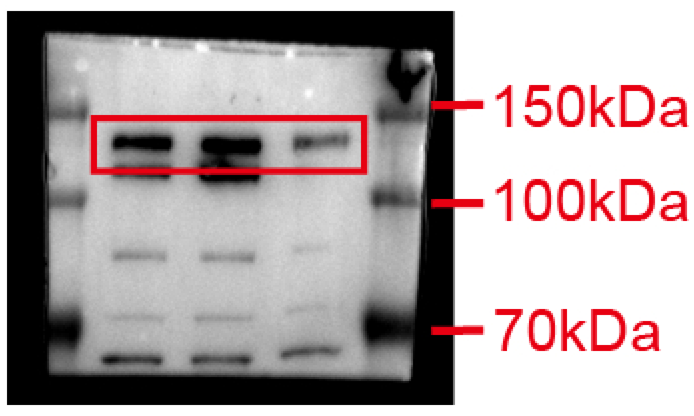

GAPDH (60004-1-Ig, Proteintech)

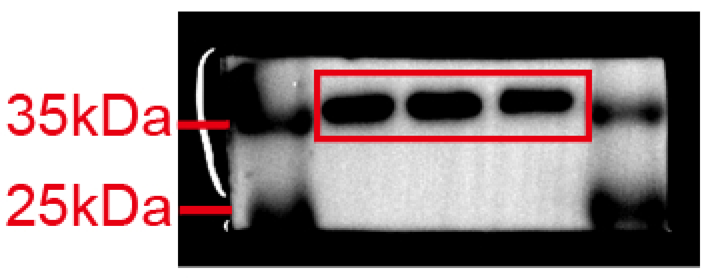

Figure 5A

COL1A1 (GB114197, Servicebio)

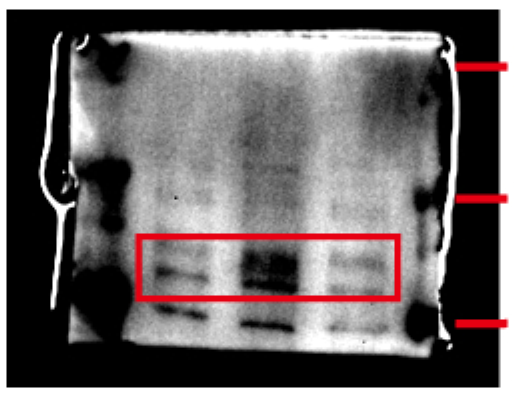

α-SMA (14395-1-AP, Proteintech)

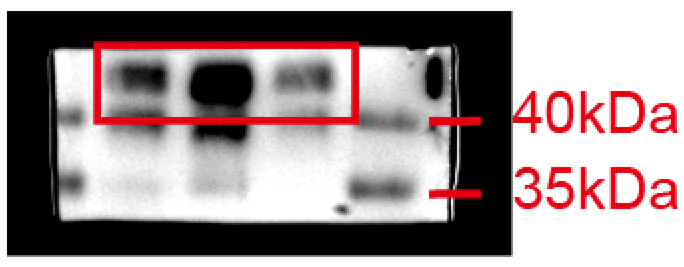

Tubulin (11224-1-AP, Proteintech)

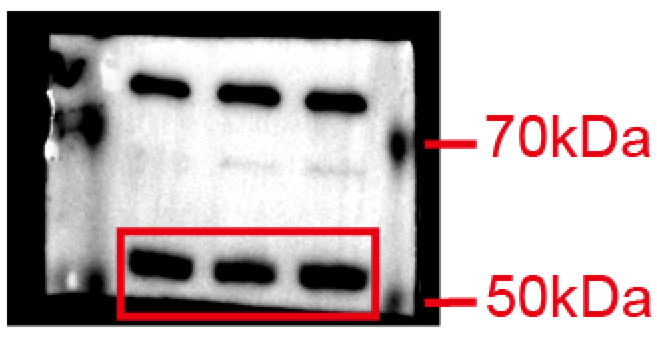

Figure 5G

FASN (66591-1-Ig, Proteintech)

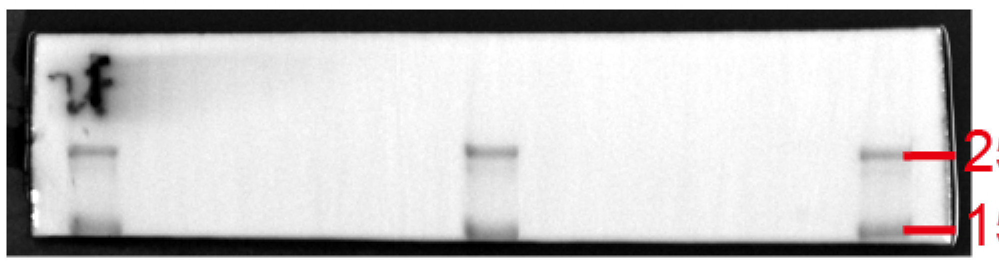

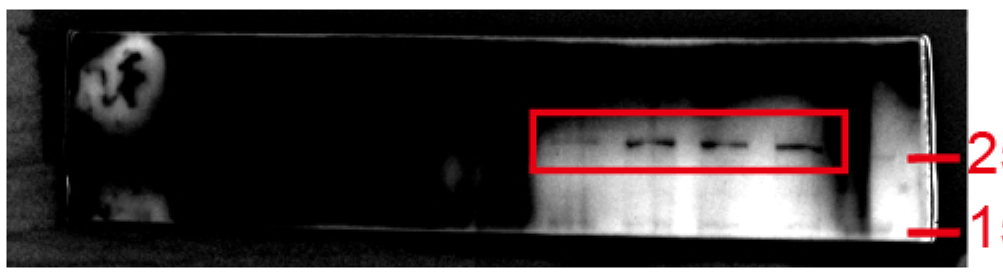

SREBP (A25305, abclonal)

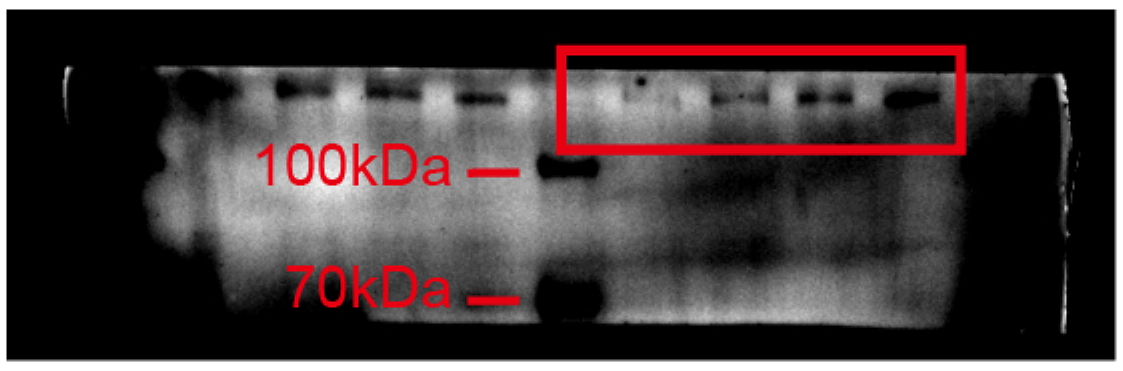

PPARγ (66936-1-Ig, Proteintech)

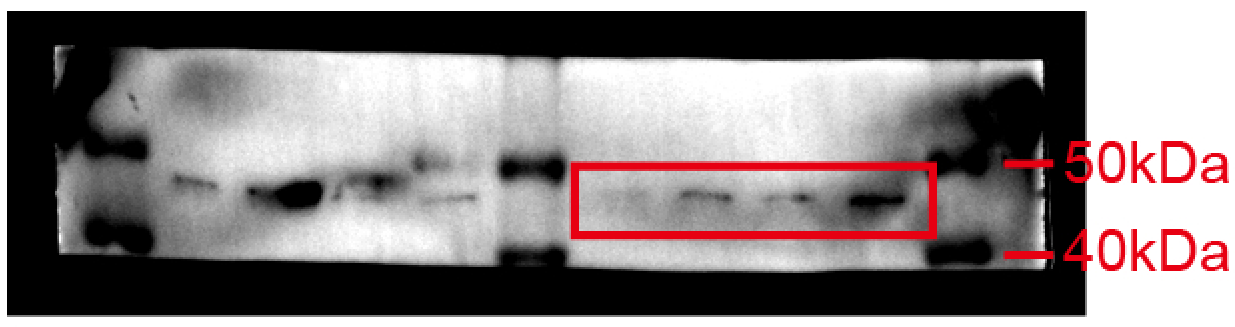

GAPDH (60004-1-Ig, Proteintech)

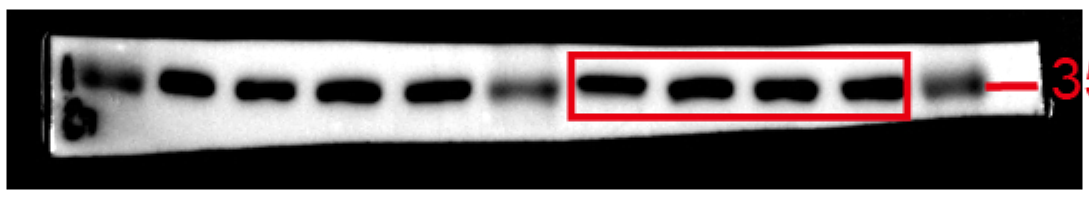

Figure S3J

FASN (66591-1-Ig, Proteintech)

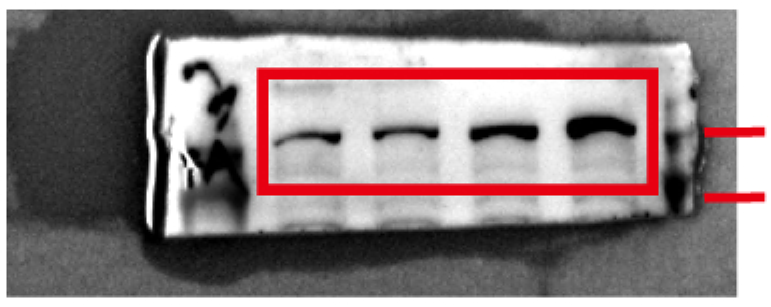

SREBP (A25305, abclonal)

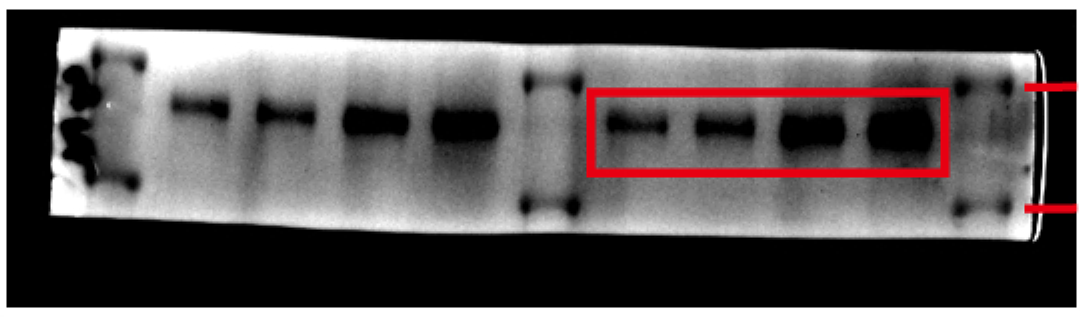

PPARγ (66936-1-Ig, Proteintech)

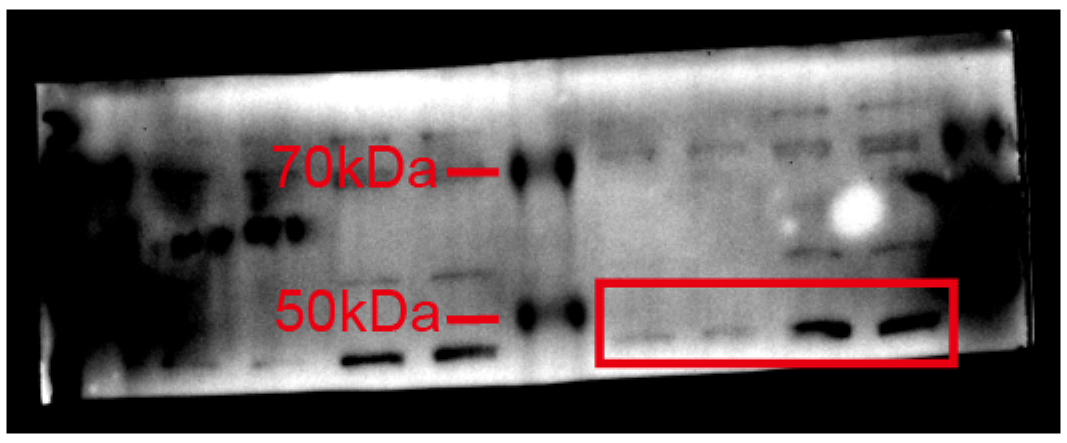

GAPDH (60004-1-Ig, Proteintech)

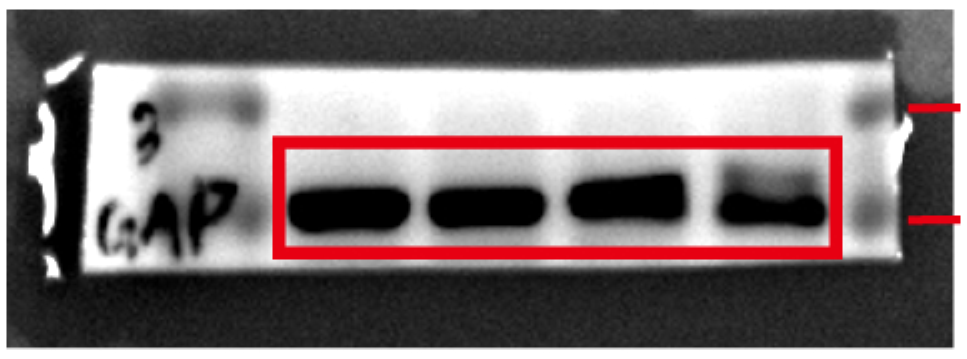

Figure S6E

CREB1 (12208-1-AP, Proteintech)

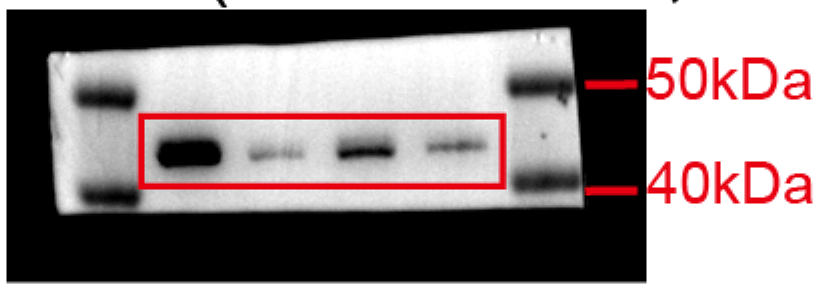

p-CREB1 (28792-1-AP, Proteintech)

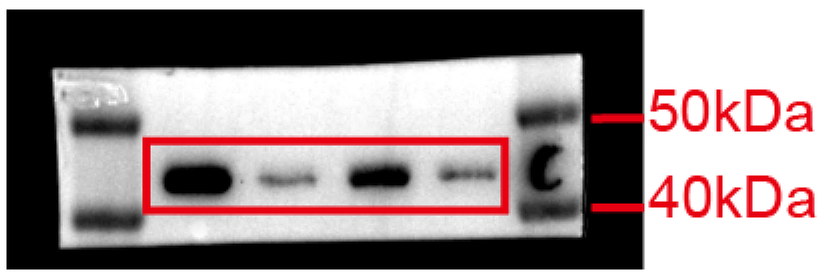

GAPDH (60004-1-Ig, Proteintech)

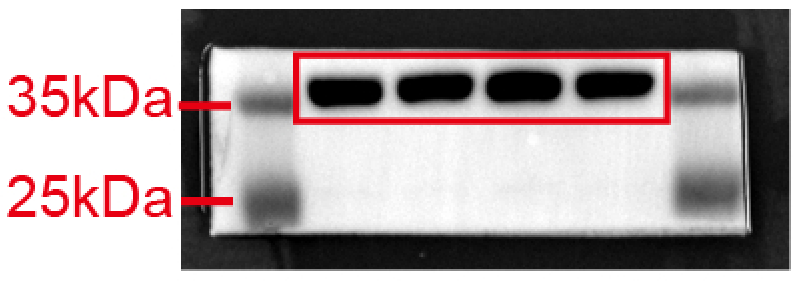

Figure S6F

MMP-9 (10375-2-AP, Proteintech)

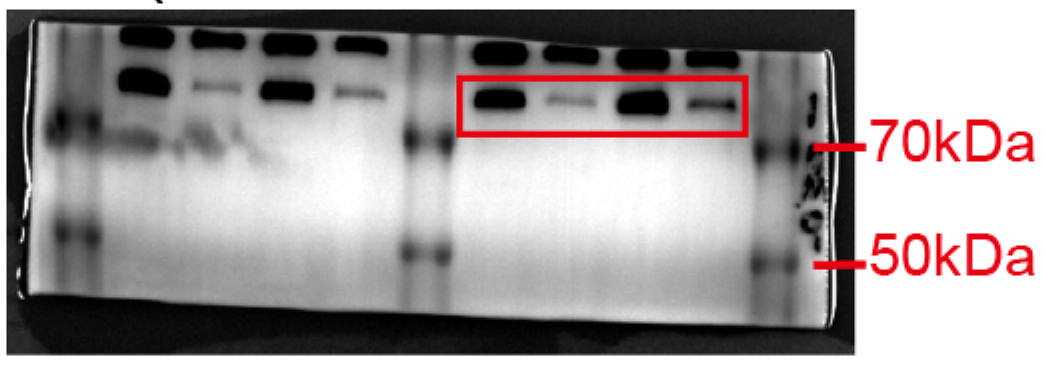

E-cadherin (GB11082, Servicebio)

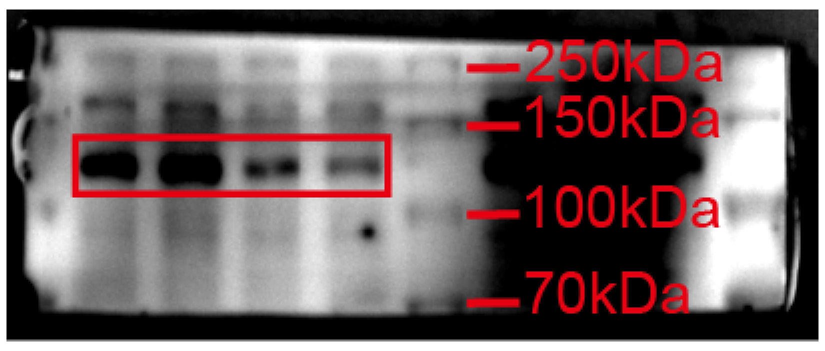

N-cadherin (GB12135, Servicebio)

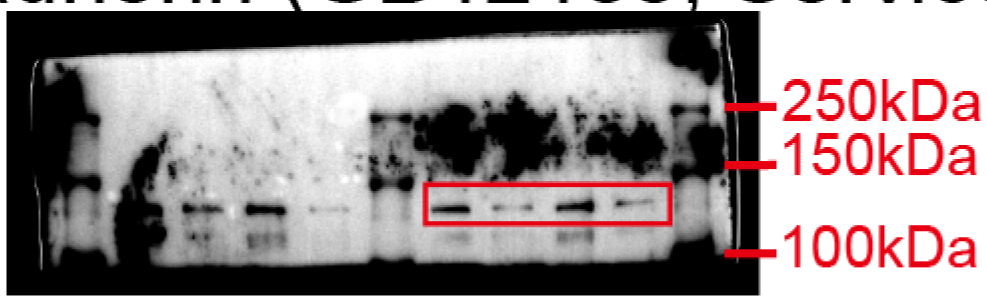

GAPDH (60004-1-Ig, Proteintech)

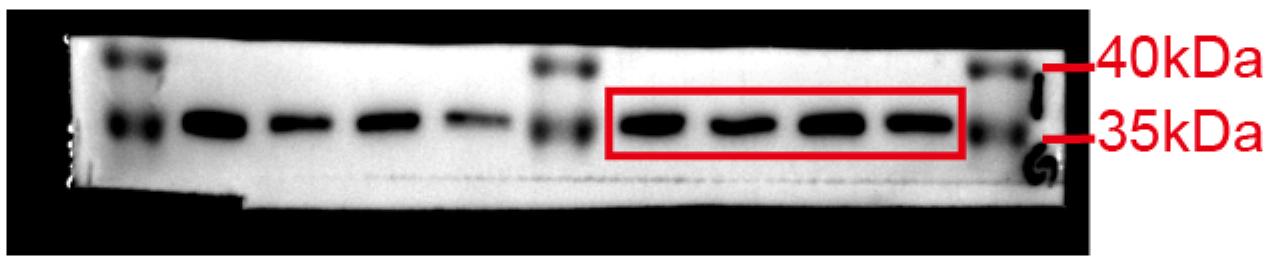

Figure S11A

CREB1 (12208-1-AP, Proteintech)

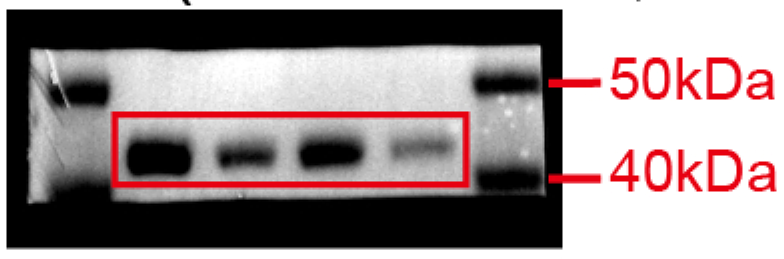

p-CREB1 (28792-1-AP, Proteintech)

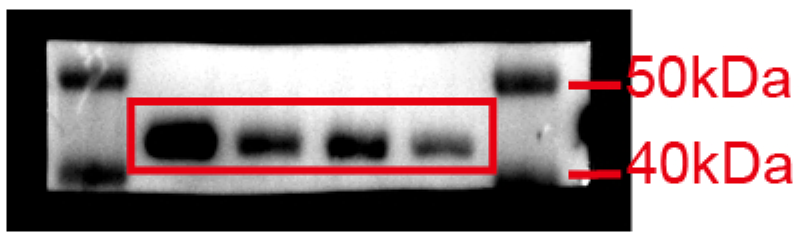

GAPDH (60004-1-Ig, Proteintech)

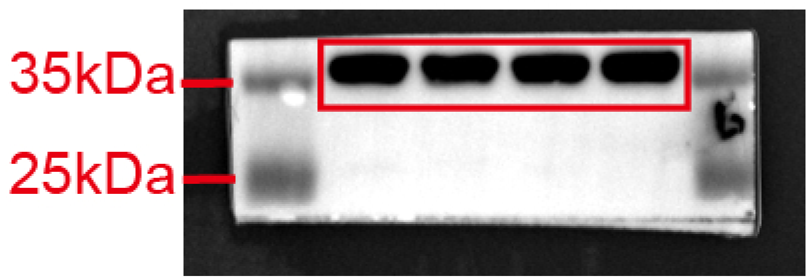

Figure S11E

CREB1 (12208-1-AP, Proteintech)

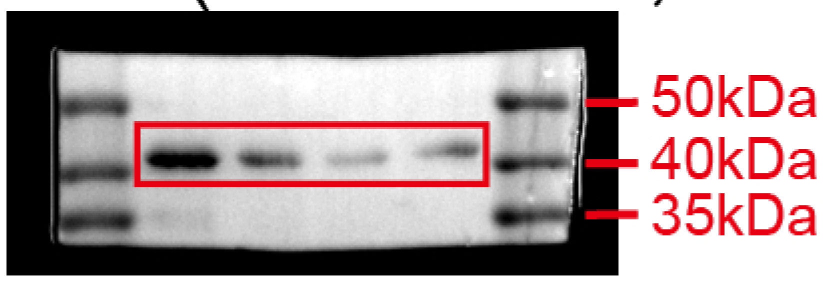

p-CREB1 (28792-1-AP, Proteintech)

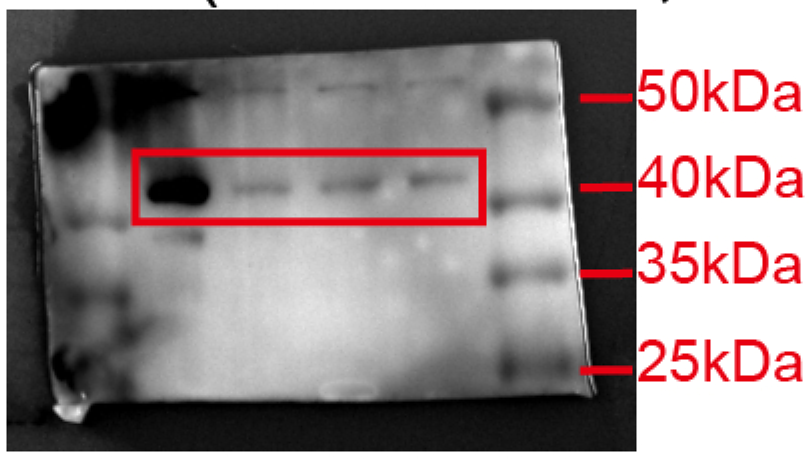

TGF-β1 (ab215715, Abcam)

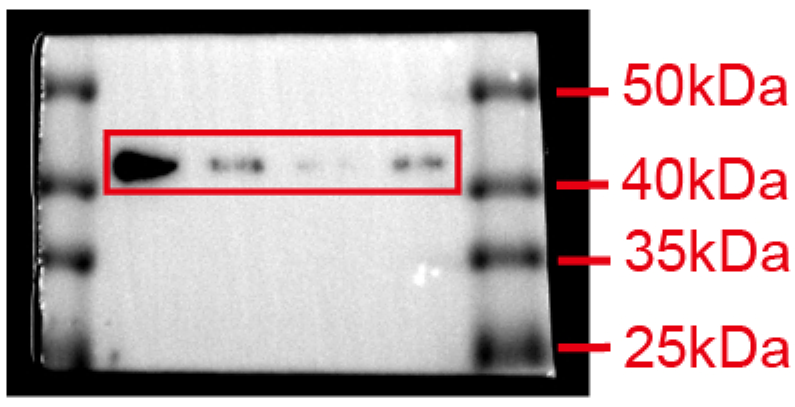

MMP-9 (10375-2-AP, Proteintech)

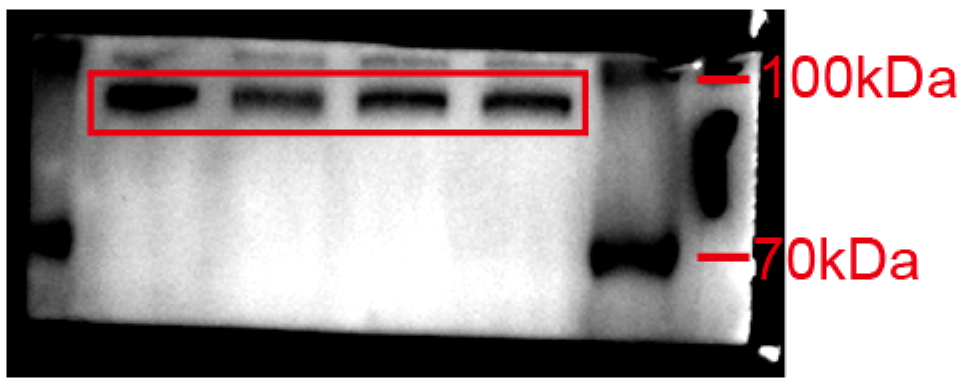

N-cadherin (GB12135, Servicebio)

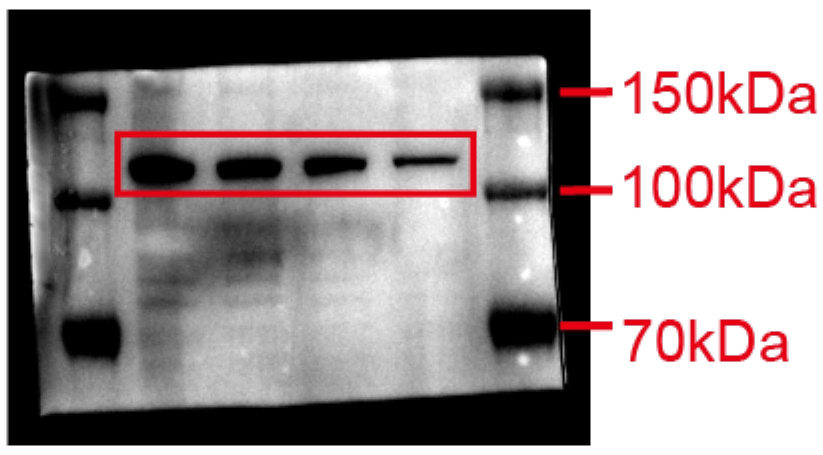

COL1A1(GB114197, Servicebio)

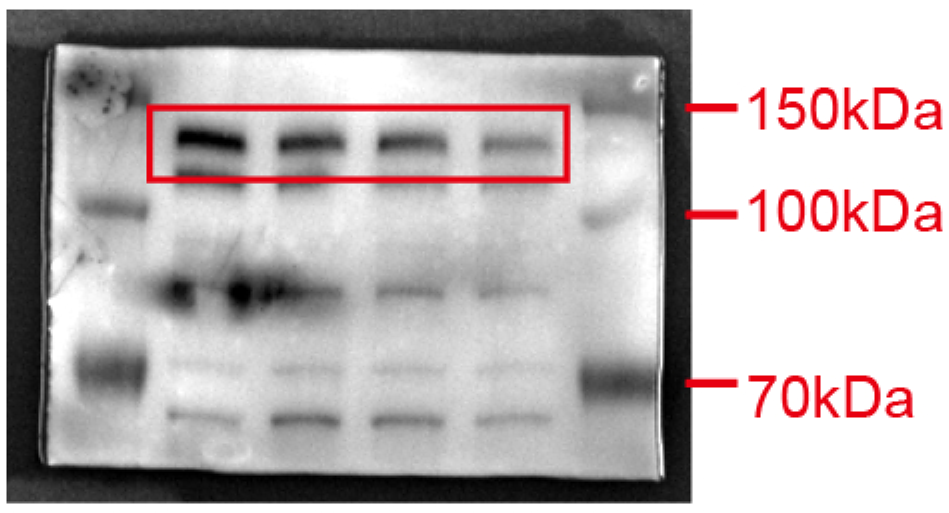

GAPDH (60004-1-Ig, Proteintech)

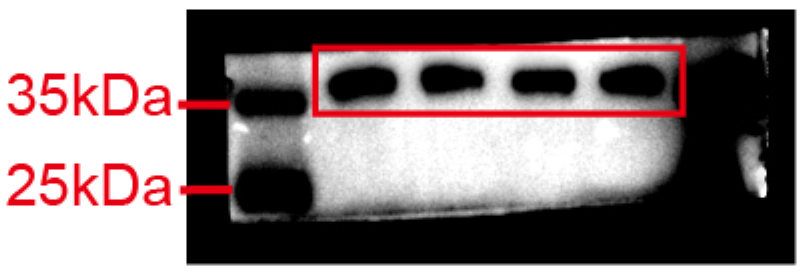

Supplement: Supplementary file 3 — Original Western blot images [file 41419_2026_8625_MOESM3_ESM.pdf]
