# Supplementary figures and images for: The DAG/PKC/CREB1/TGF-β1 axis drives shear-wave elastography stiffness and malignant progression in triple-negative breast cancer via lipid metabolic reprogramming
Source: Cell Death Dis. 2026 Mar 20;17(1):327. doi: 10.1038/s41419-026-08625-0 (PMC13039978; doi:10.1038/s41419-026-08625-0)

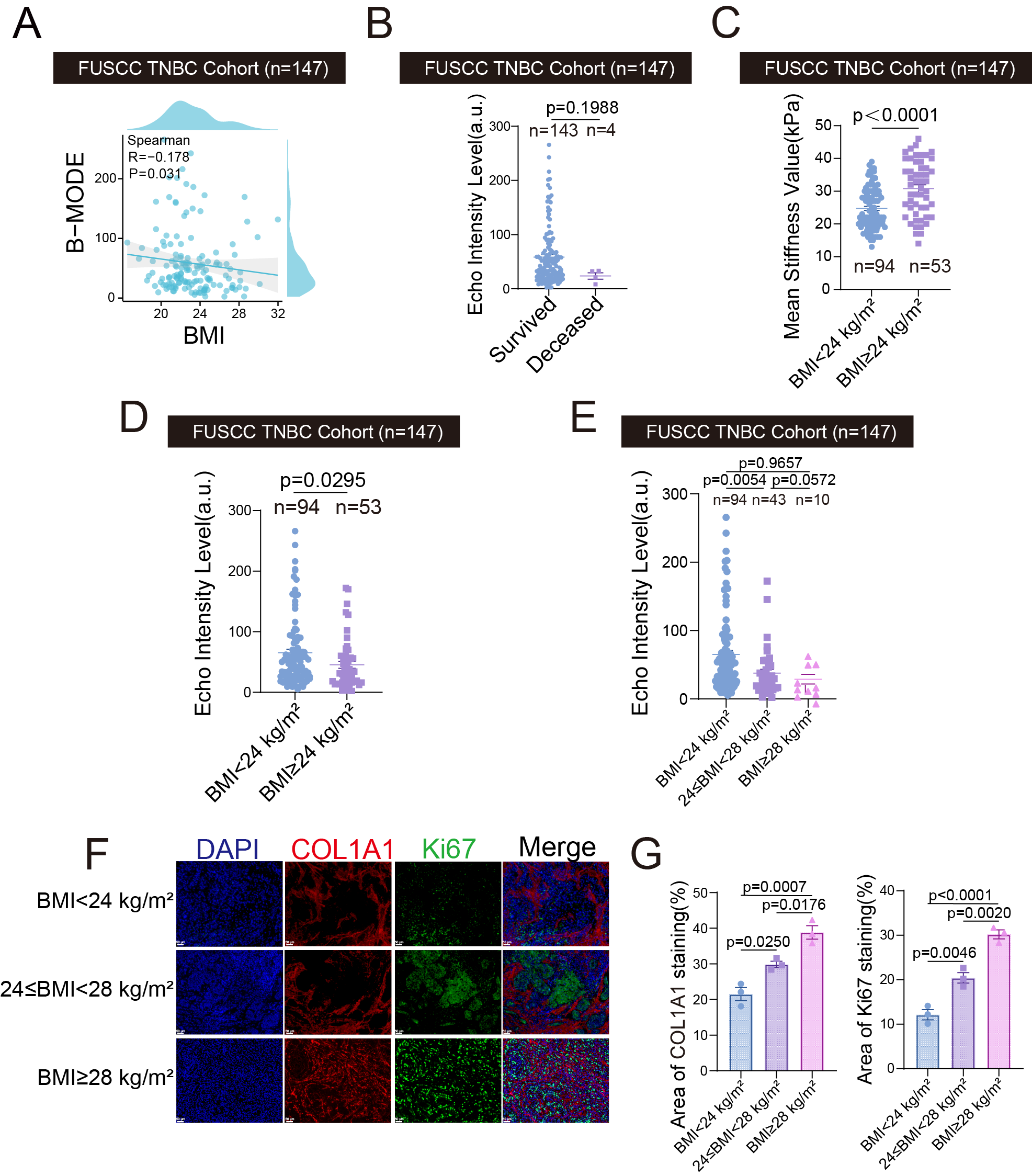

Supplement: Supplementary file 5 — Supplementary Figure S1 [file 41419_2026_8625_MOESM5_ESM.png]

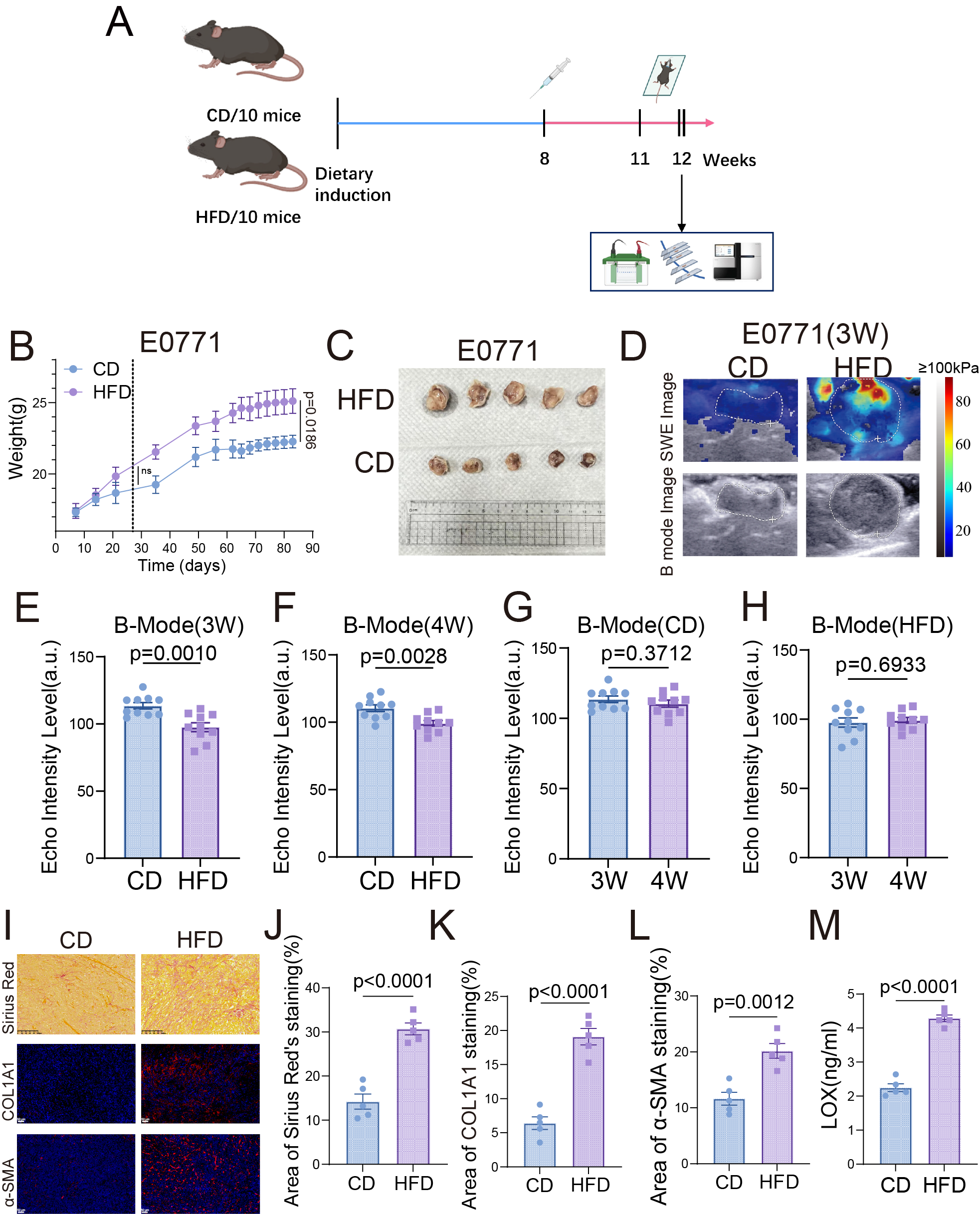

Supplement: Supplementary file 6 — Supplementary Figure S2 [file 41419_2026_8625_MOESM6_ESM.png]

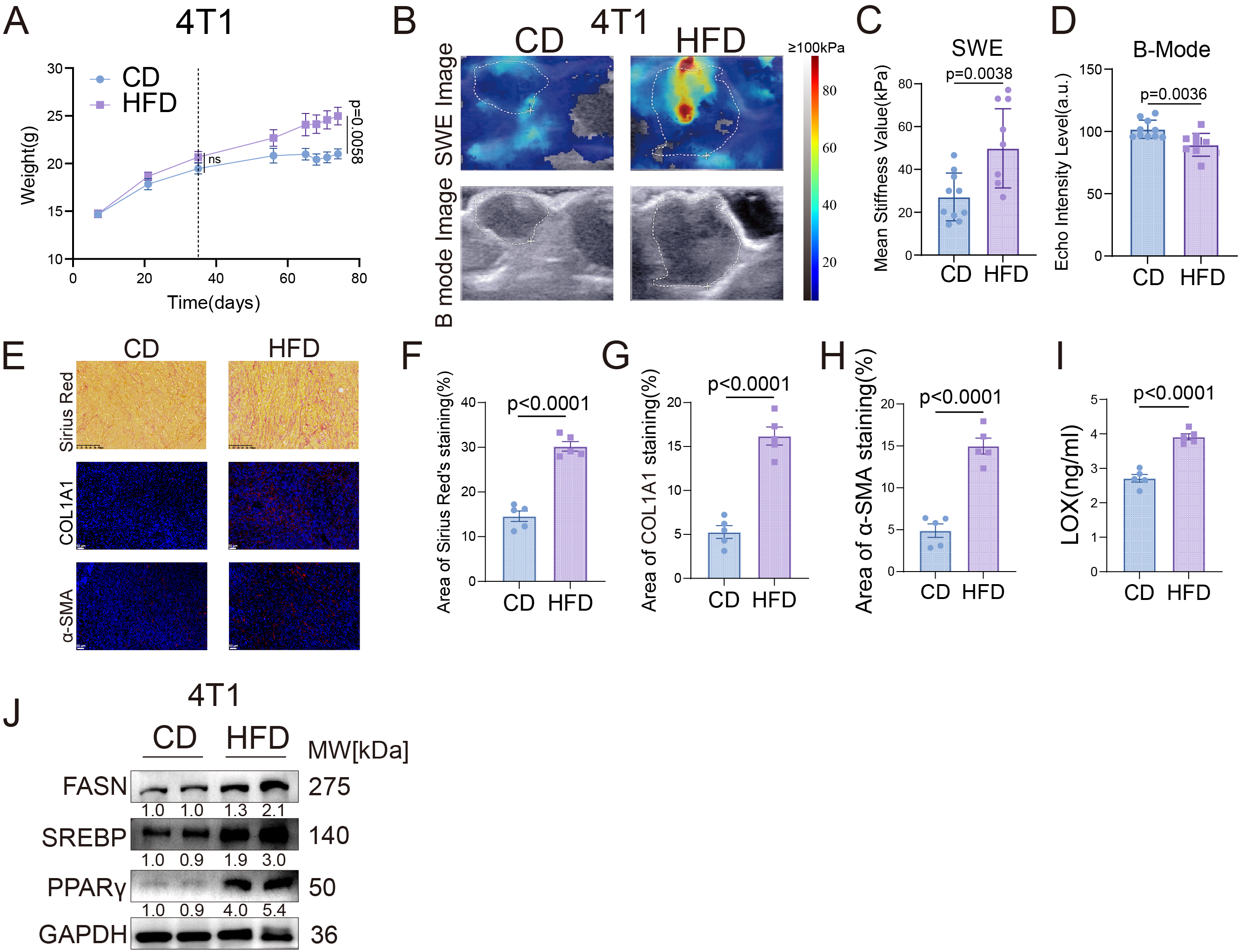

Supplement: Supplementary file 7 — Supplementary Figure S3 [file 41419_2026_8625_MOESM7_ESM.png]

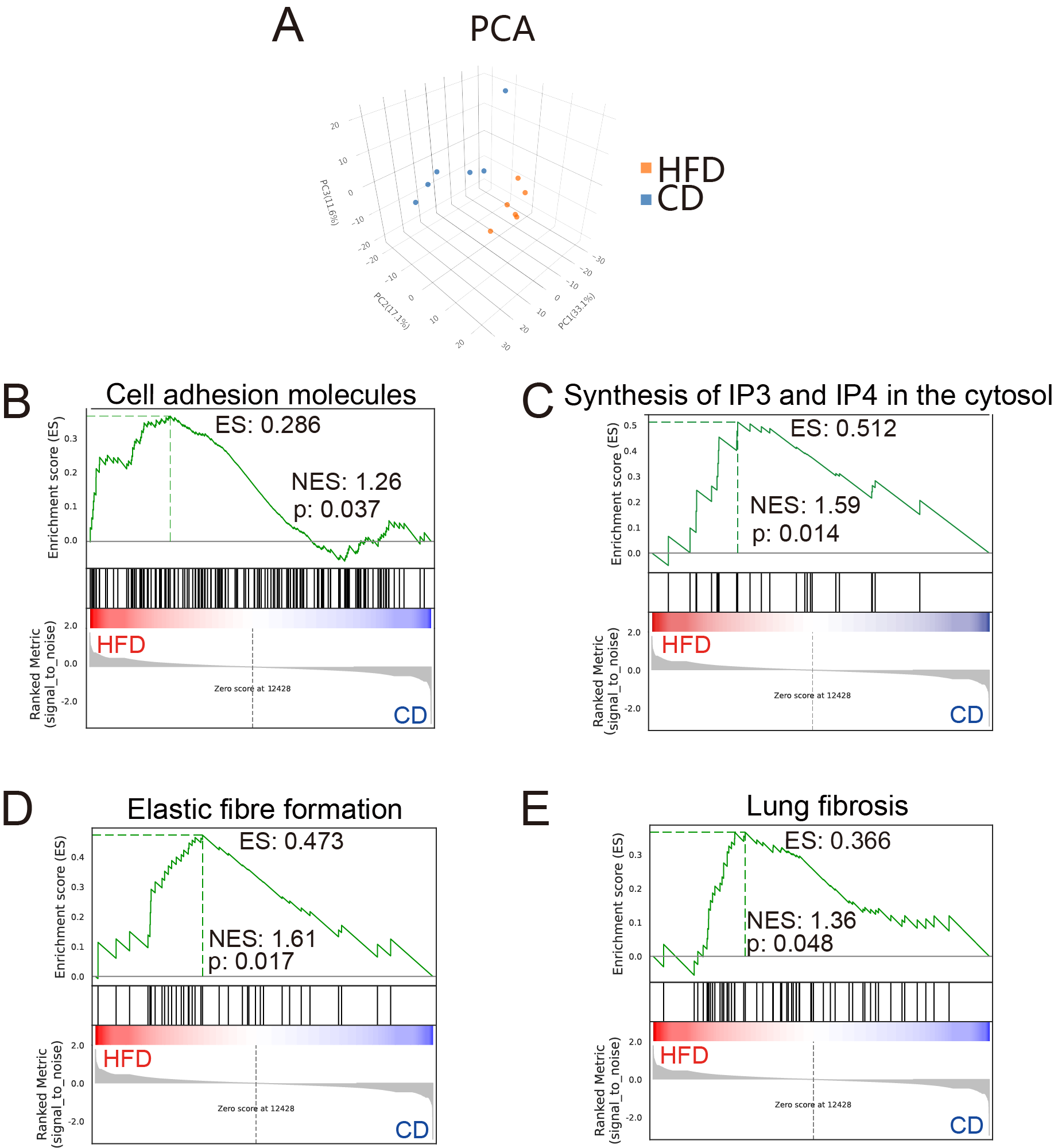

Supplement: Supplementary file 8 — Supplementary Figure S4 [file 41419_2026_8625_MOESM8_ESM.png]

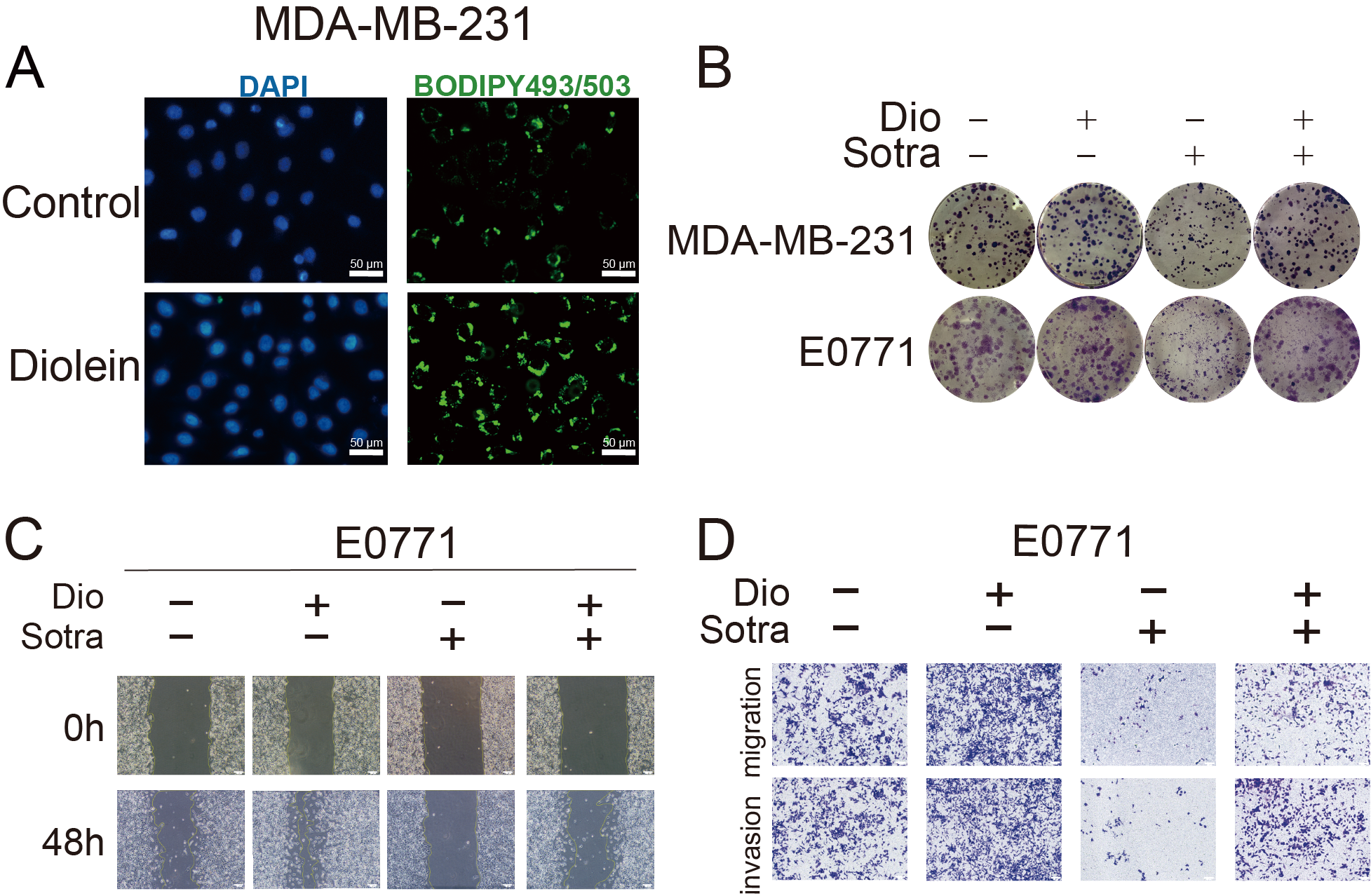

Supplement: Supplementary file 9 — Supplementary Figure S5 [file 41419_2026_8625_MOESM9_ESM.png]

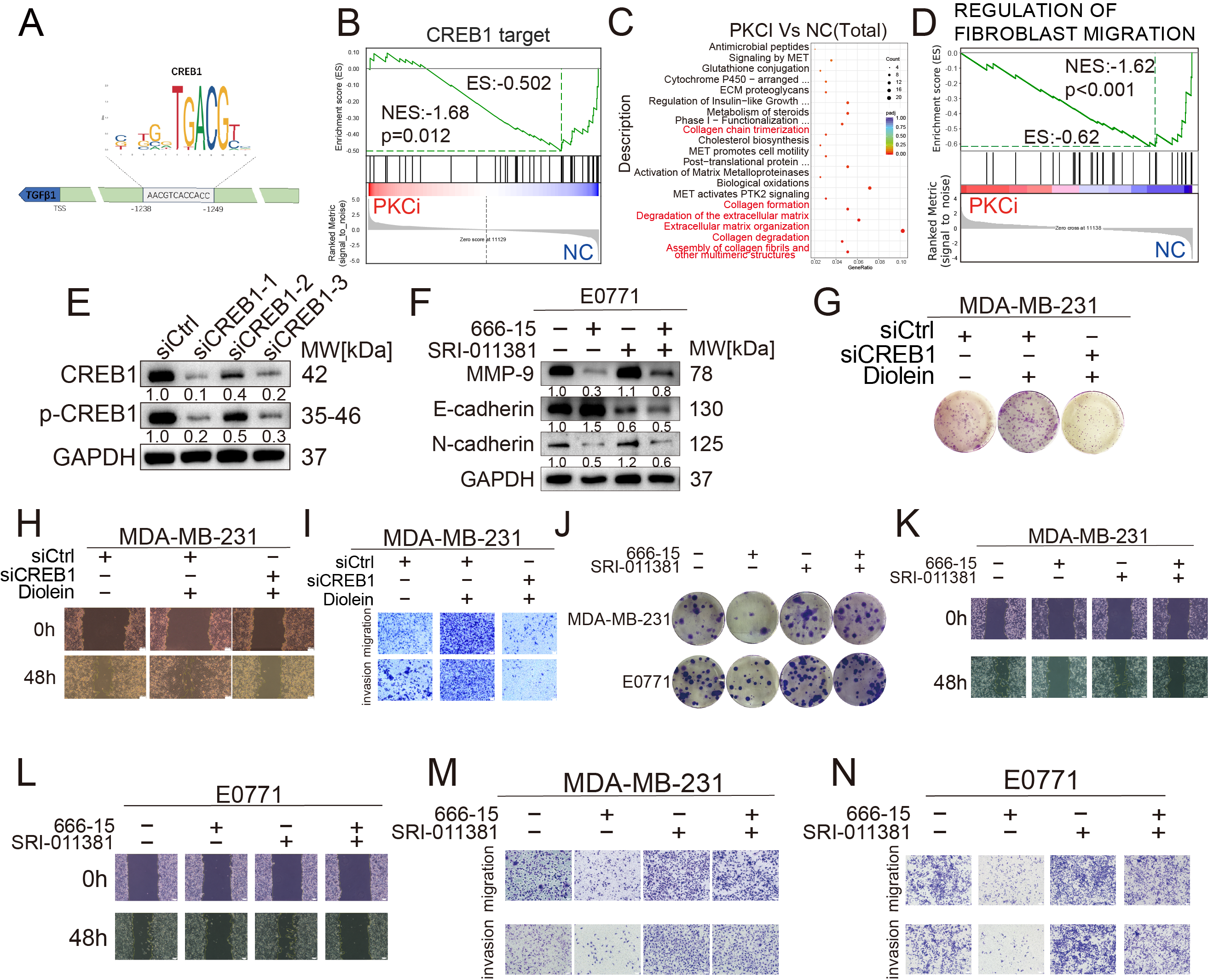

Supplement: Supplementary file 10 — Supplementary Figure S6 [file 41419_2026_8625_MOESM10_ESM.png]

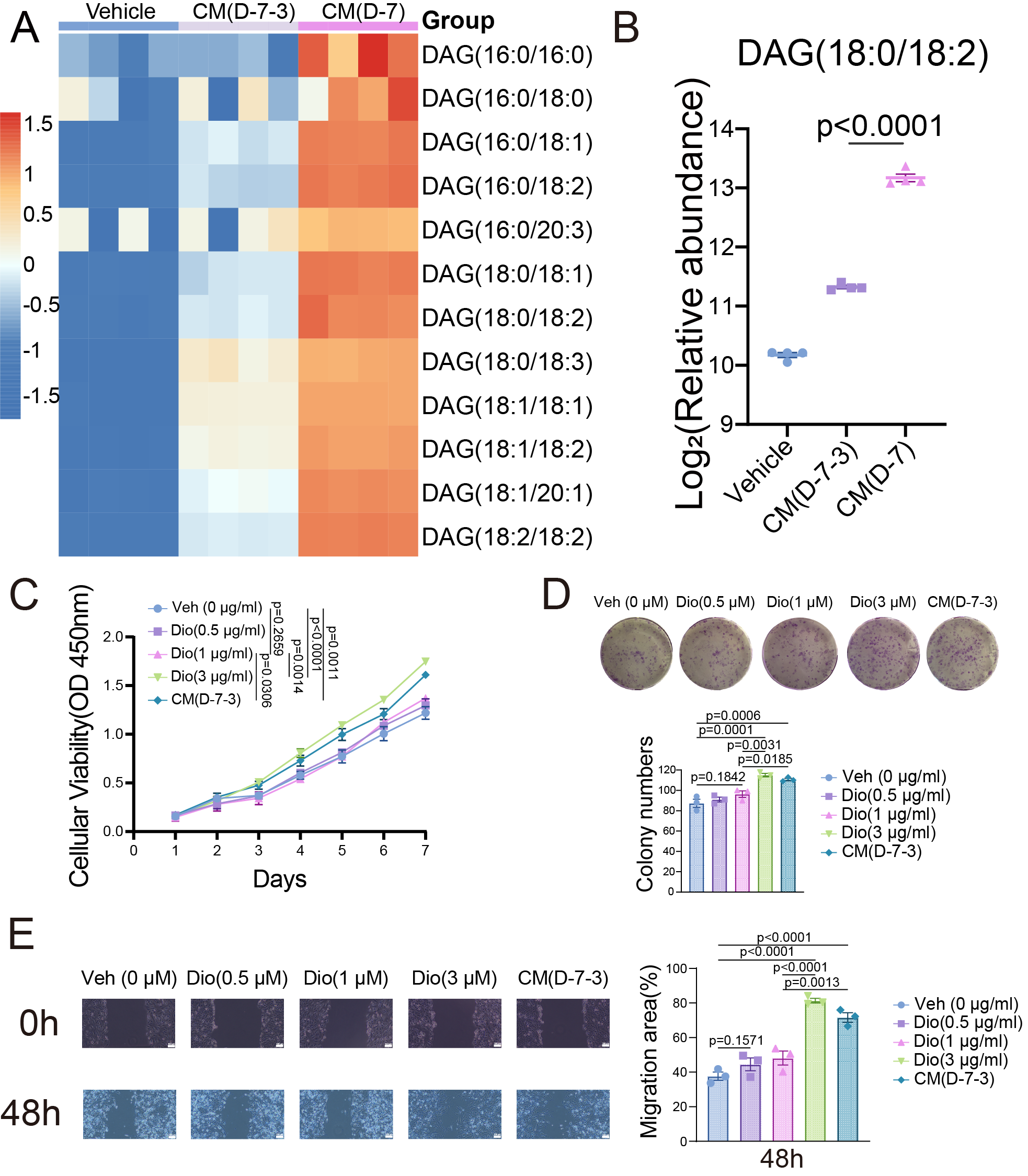

Supplement: Supplementary file 11 — Supplementary Figure S7 [file 41419_2026_8625_MOESM11_ESM.png]

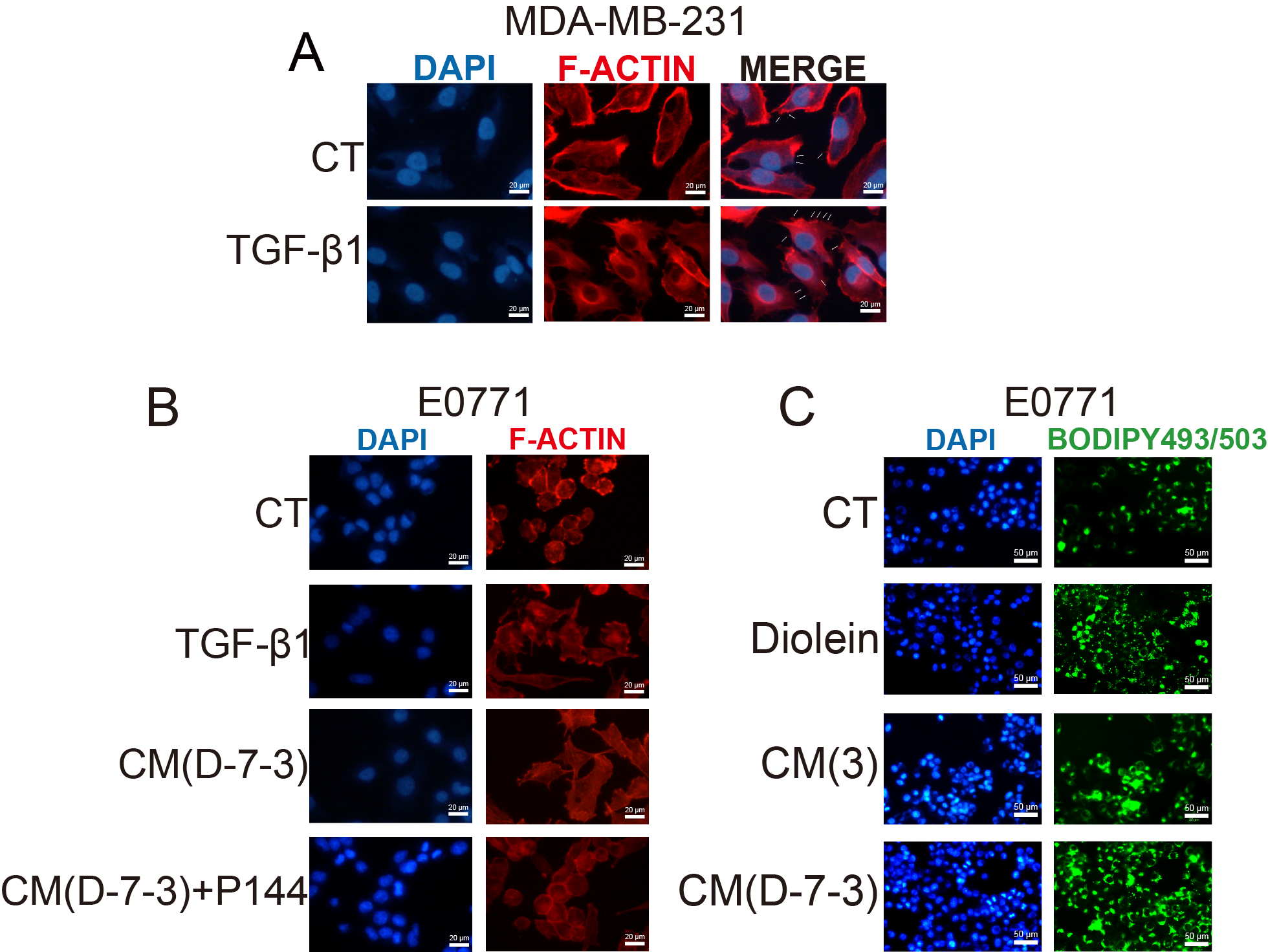

Supplement: Supplementary file 12 — Supplementary Figure S8 [file 41419_2026_8625_MOESM12_ESM.png]

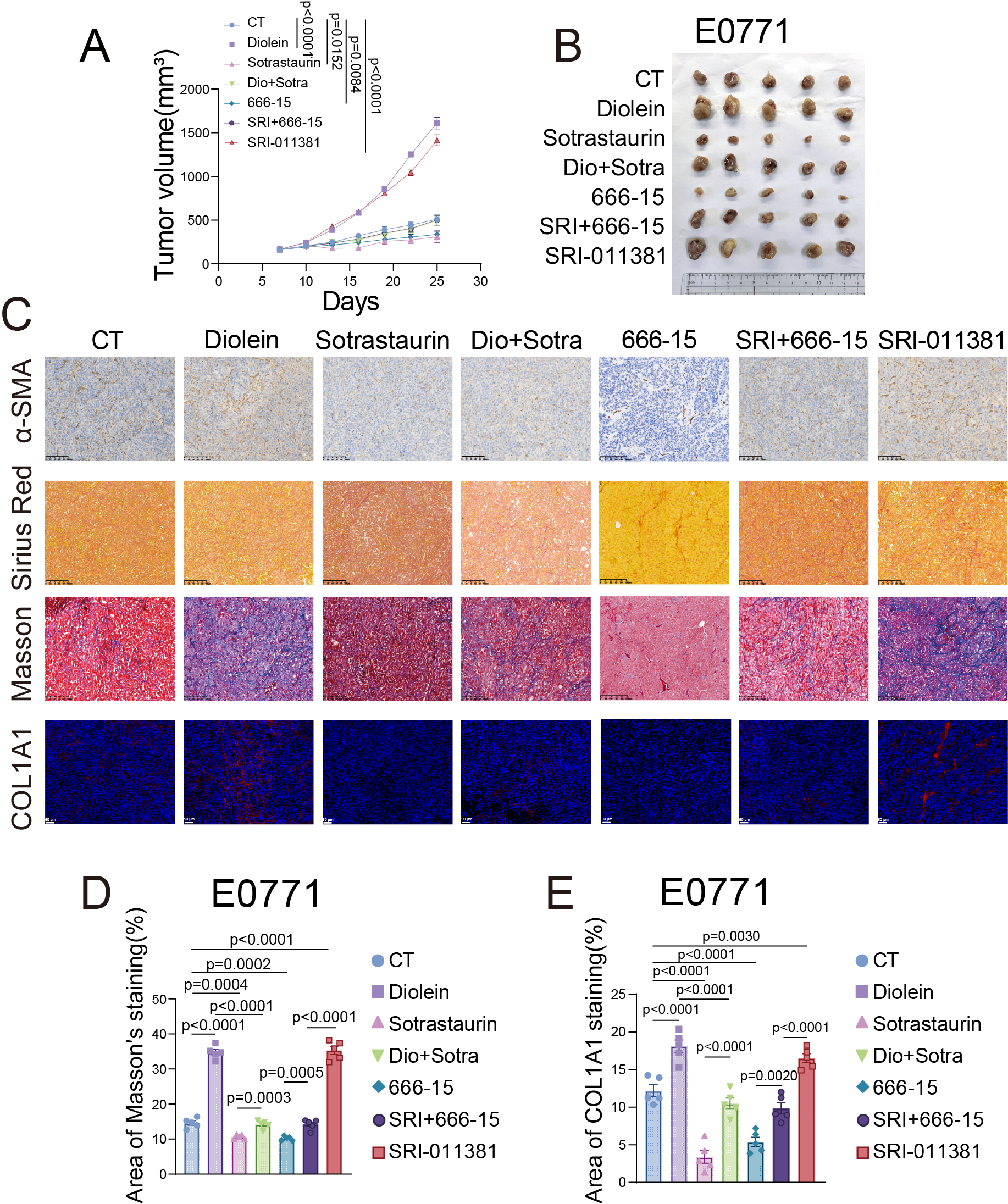

Supplement: Supplementary file 13 — Supplementary Figure S9 [file 41419_2026_8625_MOESM13_ESM.png]

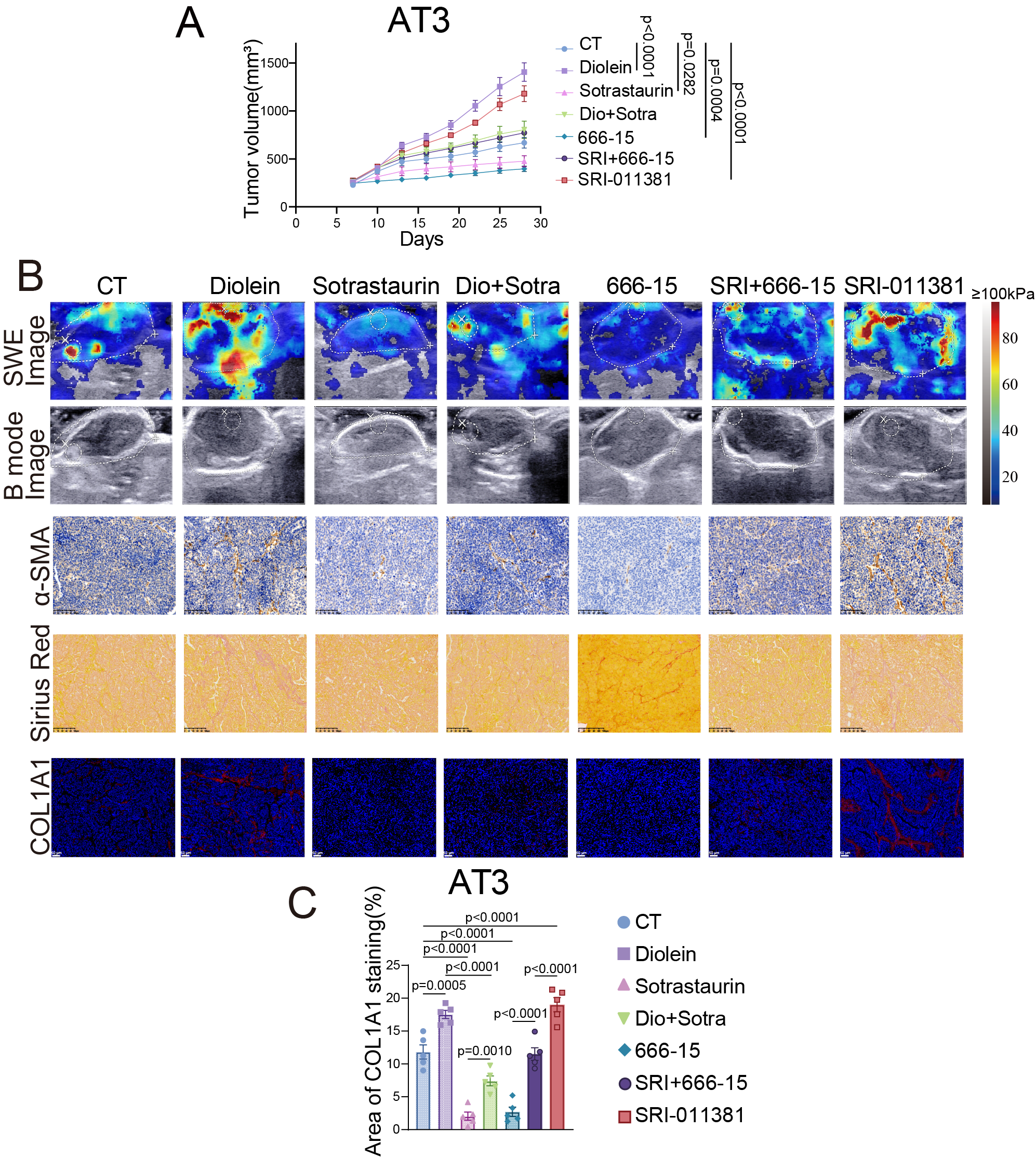

Supplement: Supplementary file 14 — Supplementary Figure S10 [file 41419_2026_8625_MOESM14_ESM.png]

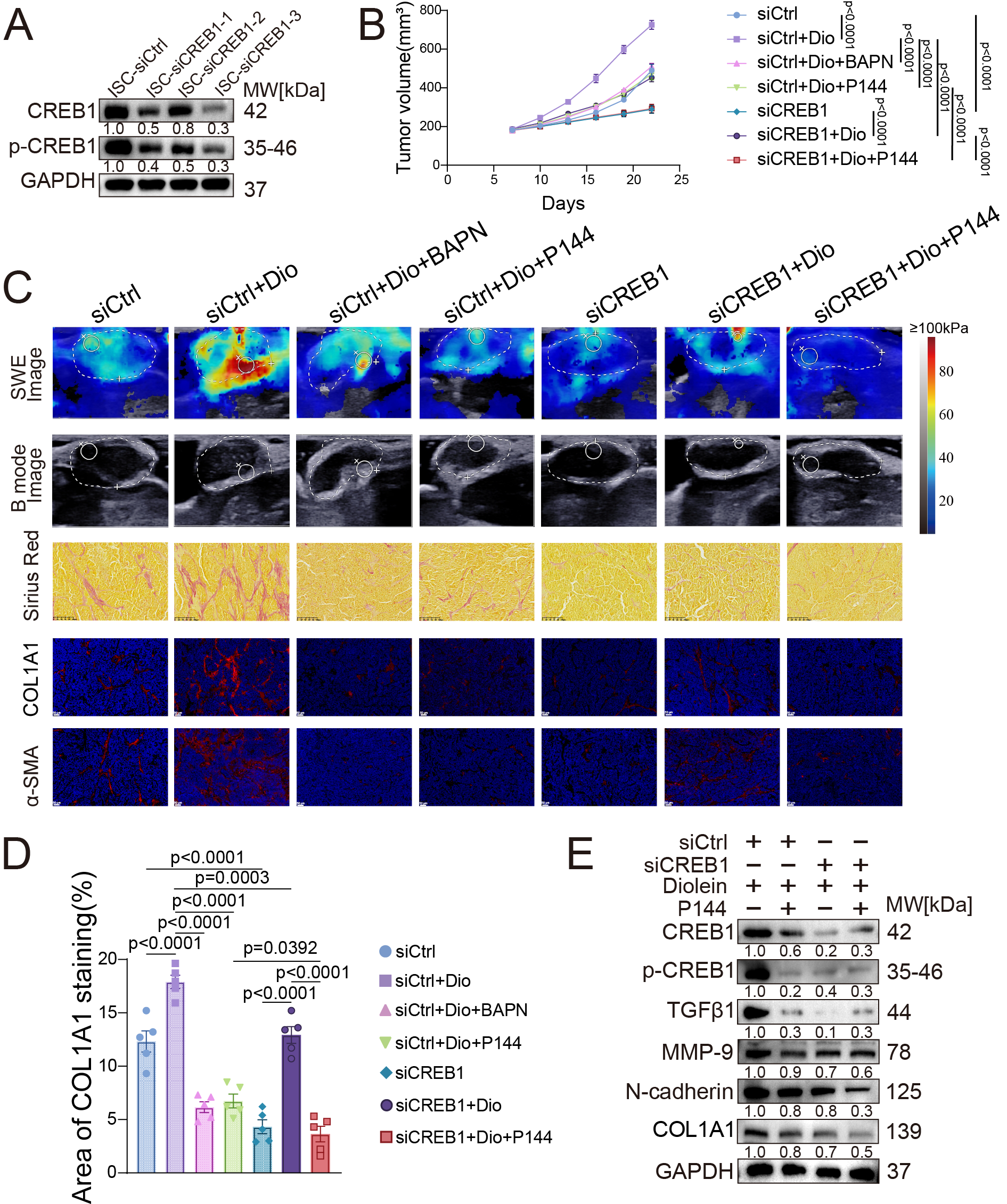

Supplement: Supplementary file 15 — Supplementary Figure S11 [file 41419_2026_8625_MOESM15_ESM.png]

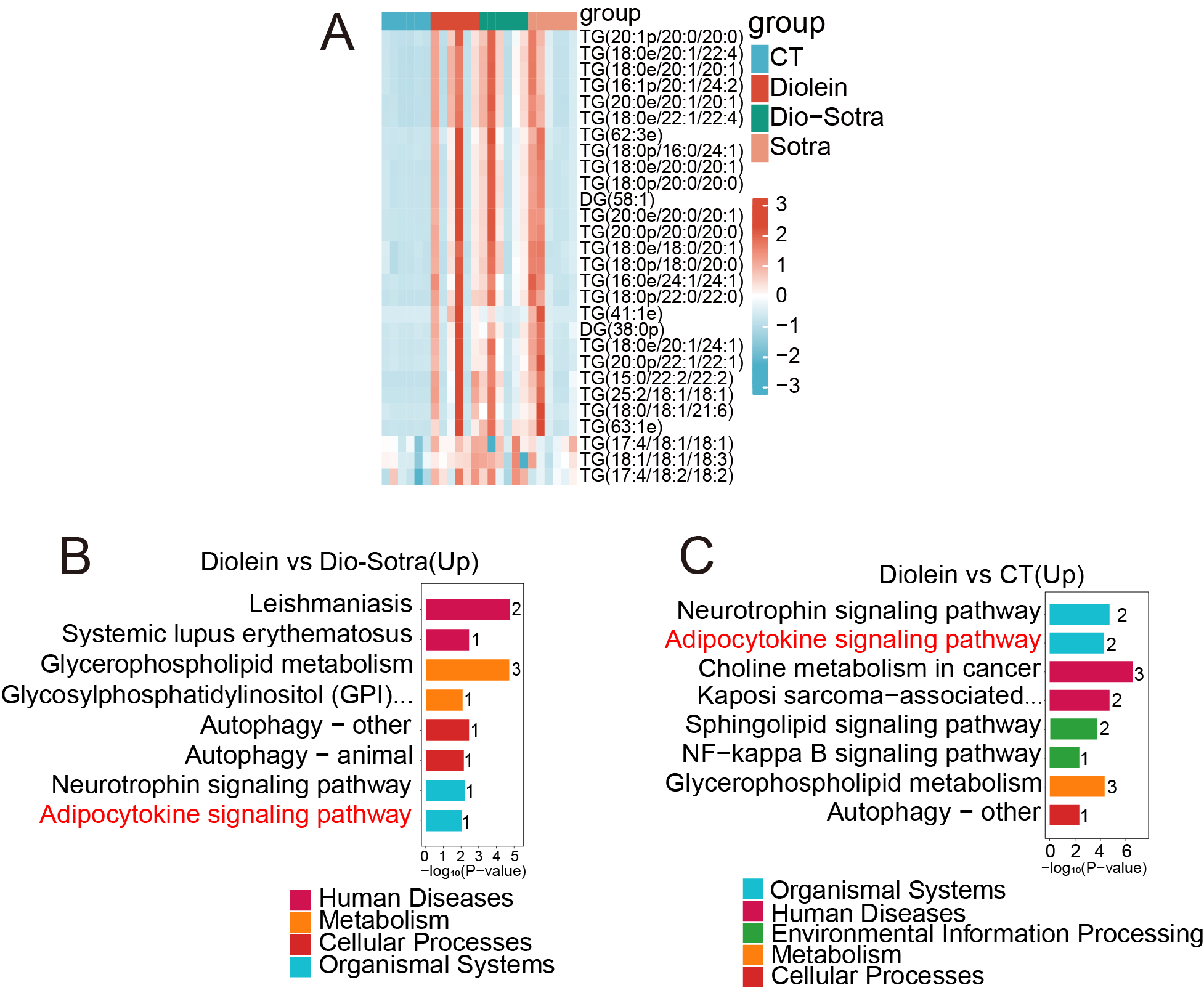

Supplement: Supplementary file 16 — Supplementary Figure S12 [file 41419_2026_8625_MOESM16_ESM.png]

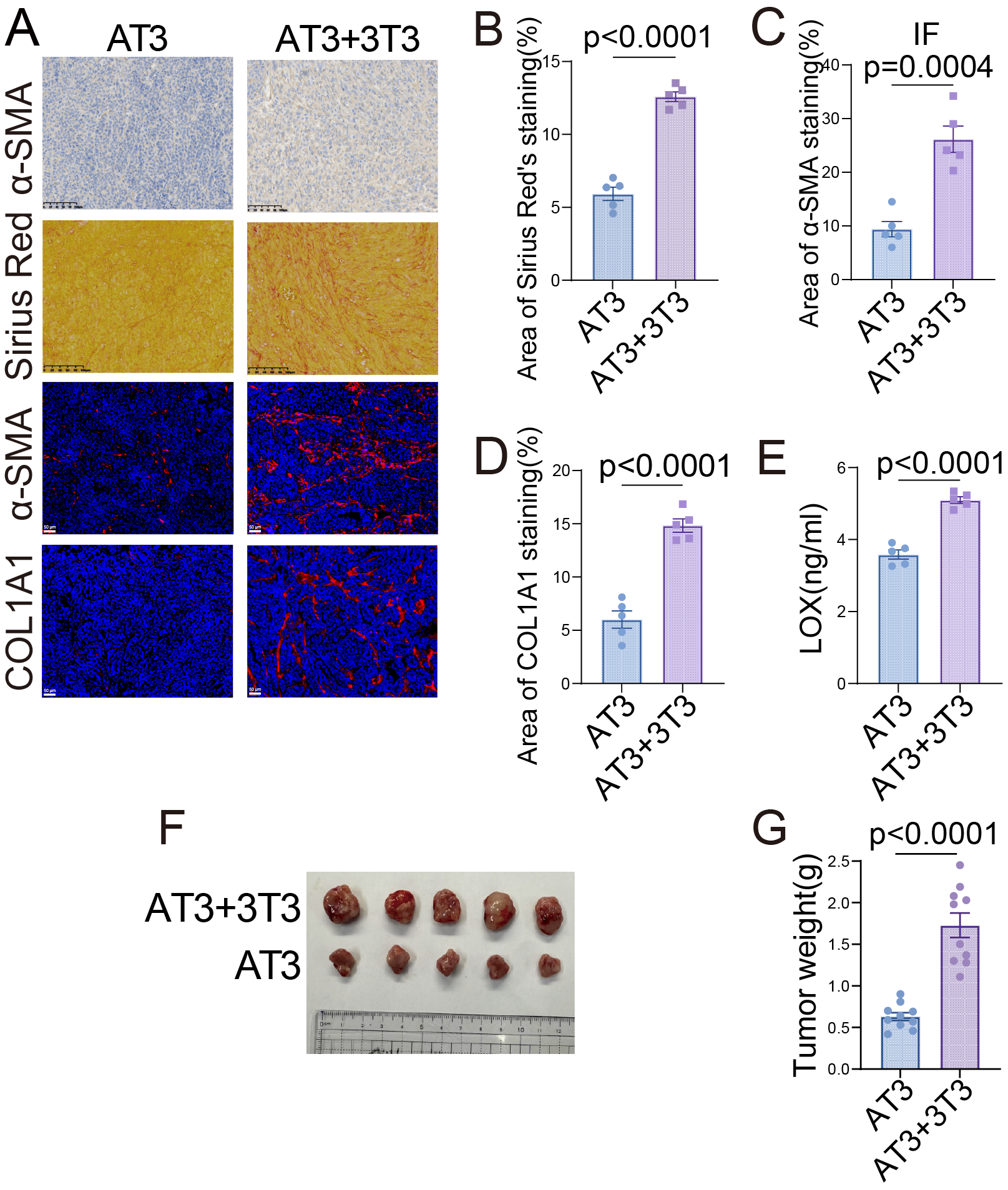

Supplement: Supplementary file 17 — Supplementary Figure S13 [file 41419_2026_8625_MOESM17_ESM.png]

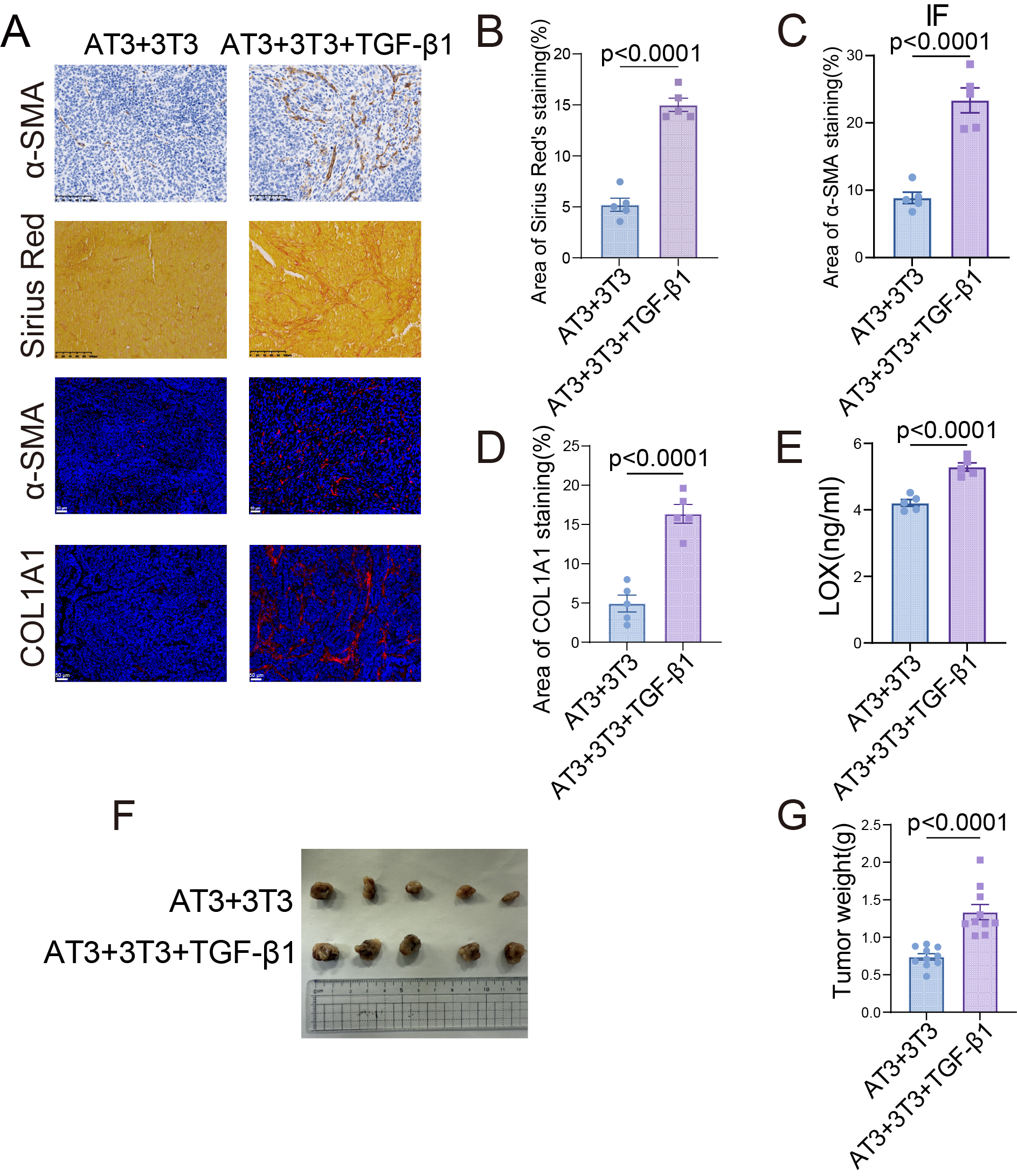

Supplement: Supplementary file 18 — Supplementary Figure S14 [file 41419_2026_8625_MOESM18_ESM.png]

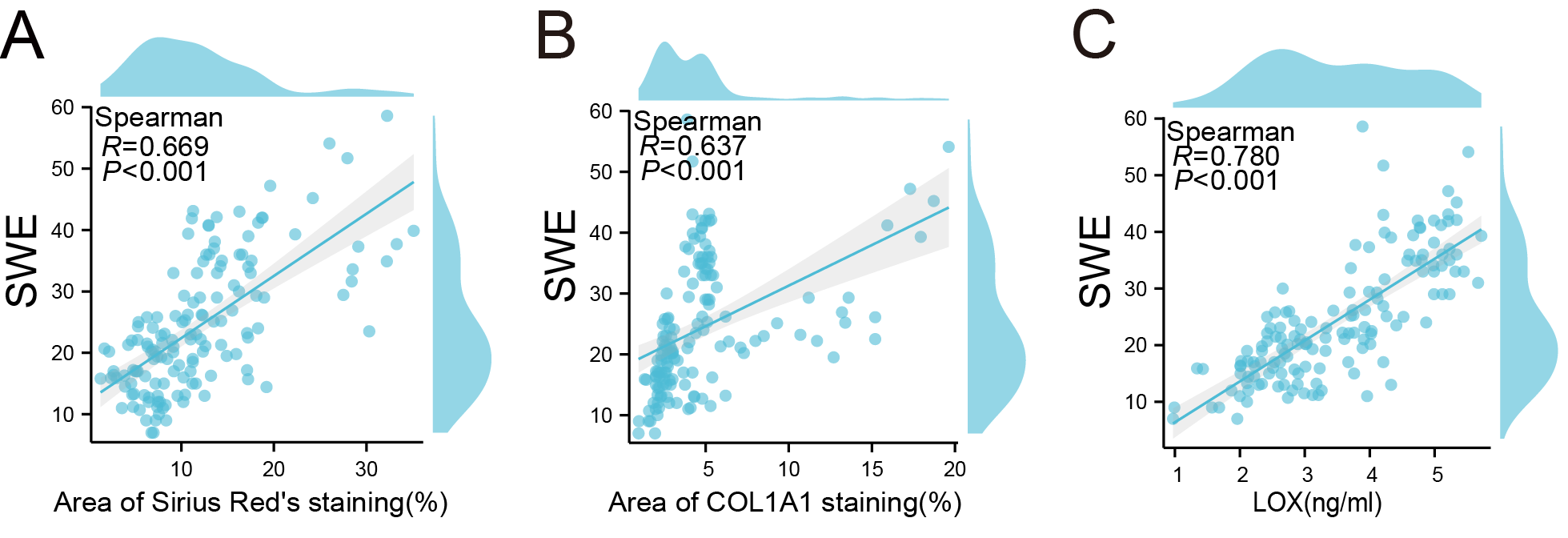

Supplement: Supplementary file 19 — Supplementary Figure S15 [file 41419_2026_8625_MOESM19_ESM.png]

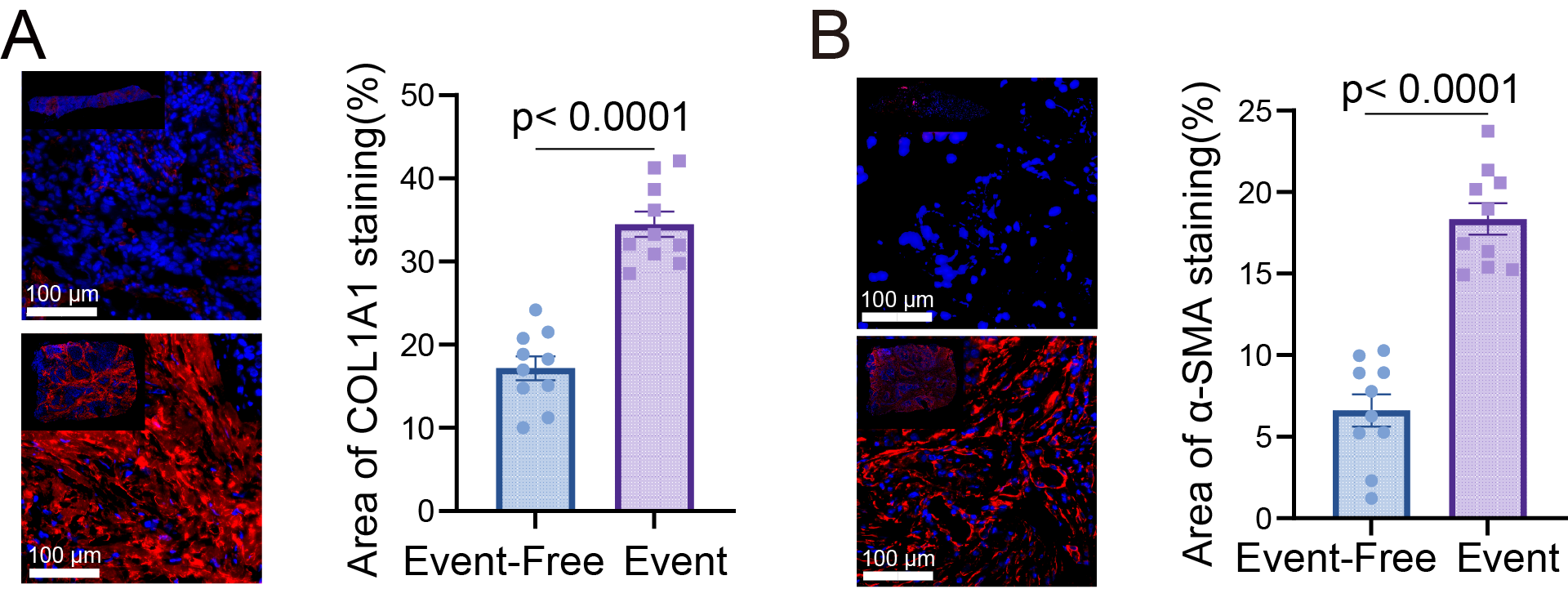

Supplement: Supplementary file 20 — Supplementary Figure S16 [file 41419_2026_8625_MOESM20_ESM.png]
