# Supplementary material for: The DAG/PKC/CREB1/TGF-β1 axis drives shear-wave elastography stiffness and malignant progression in triple-negative breast cancer via lipid metabolic reprogramming
Source: Cell Death Dis. 2026 Mar 20;17(1):327. doi: 10.1038/s41419-026-08625-0 (PMC13039978; doi:10.1038/s41419-026-08625-0)
Supplement: Supplementary file 21 — Supplementary Table S1 [file 41419_2026_8625_MOESM21_ESM.docx]

| Antibody | Vendor | Cat# | Hosts | Working concentration |
| --- | --- | --- | --- | --- |
| FASN | Proteintech | 66591-1-Ig | M | 1:10000 |
| GAPDH | Proteintech | 60004-1-Ig | M | 1:100000 |
| SREBP | abclonal | A25305 | R | 1:1000 |
| PPARG | Proteintech | 66936-1-Ig | M | 1:10000 |
| PKC | abclonal | A24003 | R | 1:5000 (WB) |
| PKC | Proteintech | 12919-1-AP | R | 1:200 (IHC) |
| p-PKC | abclonal | AP1414 | R | 1:1000 |
| MMP-9 | Proteintech | 10375-2-AP | R | 1:2000 |
| E-cadherin | Servicebio | GB11082 | R | 1:1000 |
| N-cadherin | Servicebio | GB12135 | M | 1:1000 |
| CREB1 | Proteintech | 12208-1-AP | M | 1:20000 |
| p-CREB1 | Proteintech | 28792-1-AP | R | 1:2000 |
| Tubulin | Proteintech | 11224-1-AP | R | 1:10000 |
| TGF-β1 | Abcam | ab215715 | R | 1:1000 |
| α-SMA | Proteintech | 14395-1-AP | R | 1:10000 (WB) |
| α-SMA | Servicebio | GB111364 | R | 1:1000 (IF、IHC) |
| Collagen I | Servicebio | GB114197 | R | 1:1000 (WB)、1:500 (IF) |
| Ki67 | Proteintech | 27309-1-AP | R | 1:100(IF) |
| HRP-conjugated Goat Anti-Rabbit IgG(H+L) | Proteintech | SA00001-2 |  | 1:10000(WB) |
| HRP-conjugated Goat Anti-Mouse IgG(H+L) | Proteintech | SA00001-1 |  | 1:10000(WB) |
| CoraLite594 – conjugated Goat Anti-Rabbit IgG(H+L) | Proteintech | SA00013-4 |  | 1:100(IF) |
| CoraLite488-conjugated Goat Anti-Rabbit IgG(H+L) | Proteintech | SA00013-2 |  | 1:100(IF) |
| Multi-rAb™ Polymer HRP-Goat Anti-Rabbit/Mouse Universal Recombinant Secondary Antibody (H+L) | Proteintech | RGAU011 |  | Ready to use (IHC) |

**Supplementary Table 1. Complete antibody information**
